# Greek Manuscripts at the Wellcome Library in London: A Descriptive Catalogue

**DOI:** 10.1017/mdh.2015.6

**Published:** 2015-04

**Authors:** Petros Bouras-Vallianatos

**Affiliations:** Centre for Hellenic Studies, King’s College London, Strand, WC2R 2LS, London, UK

**Keywords:** Greek/Byzantine medicine, Greek/Byzantine medical manuscripts, Greek palaeography/codicology, Wellcome Library, Medical Society of London

## Abstract

This article presents a new, detailed catalogue of the Greek manuscripts at the Wellcome Library in London. It consists of an introduction to the history of the collection and its scholarly importance, followed by separate entries for each manuscript. Each entry identifies the text(s) found in the respective manuscript – including reference to existing printed edition(s) of such texts – and gives a physical description of the codex, details on its provenance and bibliographical references.

## 1. Introduction

The Wellcome Library currently owns sixteen Greek manuscripts.^1^ These can be divided into two main groups according to their provenance and date of purchase. The first group consists of five volumes (MSS 289, 354, 413, 498 and 4103), all acquired separately between 1910 and 1936, while Sir Henry Wellcome (1853–1936) was still alive. The second group forms the core of the collection and is made up of eleven codices, previously owned by the Medical Society of London [MSS M(edical)S(ociety)L(ondon) 1, 14, 52, 60, 62, 109, 112, 114, 124, 126 and 135].^2^ These were part of the library’s largest acquisition since Sir Henry Wellcome’s death, viz., about 10 000 books and 200 manuscripts initially transferred on long-term loan to the Wellcome Library in 1967 thanks to the efforts of its director Noel Poynter (1906–79), and finally purchased in 1984.

Wellcome MSS MSL 1, 14, 52, 60, 62, 109 and 114 carry uniform bindings of gilt-tooled brown leather over wooden boards, with marbled tail edges and endpapers. (All their spines have been rebacked, evidently in the late nineteenth century.) Two tools used for their corner-piece ornament are identical to those on an unsigned mid-eighteenth-century binding^3^ and to some employed by the London bookbinder and publisher John Brindley (1692?–1758).^4^ MSS 52, 62, 109 and 114 are marked as having been the property of the physician and bibliophile Anthony Askew (1722–74),^5^ other manuscripts from whose collection have very similar bindings.^6^ In the 1785 sale catalogue of Askew’s library such bindings are referred to as ‘in Russian leather’ (*corio russico*).^7^ (For brevity’s sake, I have used the same designation in my catalogue.) Except for MS.MSL.135, these manuscripts can be identified in the 1785 sale catalogue. Askew is known to have bought from the physician, collector and philanthropist Richard Mead (1673–1754) all the Greek medical manuscripts that the latter had collected.^8^ Since no record of Mead’s acquisitions survives, it is impossible to ascertain how many – and which – of Askew’s numerous codices came from him. Those now owned by the Wellcome were purchased at the above-mentioned 1785 sale by the Anglo-Irish physician James Sims (1741–1820), who sold them to the Medical Society of London in 1802.^9^

The majority of the manuscripts, thirteen in number, contain medical texts. These range in date from treatises of the Hippocratic Corpus to post-Byzantine works, but the bulk is medieval. MSS MSL 14, 60 and MS.4103 preserve various collections of Byzantine and post-Byzantine *iatrosophia*, an (as yet poorly studied) type of physician’s handbook consisting of simple recipes for use in daily practice.^10^ MS.MSL.60, in particular, contains a variety of medical compilations with either a therapeutic or diagnostic focus, eg. uroscopy, and shows how medical texts were adapted in various versions, according to the needs of Byzantine practitioners. The author best represented in the Wellcome collection (MSS MSL 52, 112 and 124) is the late Byzantine physician John Zacharias Aktouarios (ca. 1275–ca. 1330), whose work *Medical Epitome* has not been fully edited in print yet. Three manuscripts (MSS MSL 62, 109 and 114) with works by Aretaeus (ca. first century AD) and Rufus of Ephesus (ca. AD 80–ca. AD 150), Aetios of Amida (ca. first half of the sixth century AD) and Paul of Aegina (late sixth century AD-d. post 642), respectively, have so far been overlooked by modern scholars. The same applies to MS.MSL.135, whose text of Theophanes Chrysobalantes’ (ca. tenth century AD) *Medical Epitome* was not considered by Joseph Sonderkamp in his detailed study of the textual tradition of that work.^11^ The same codex also contains a previously uncatalogued abridged version of Symeon Seth’s (ca. second half of the eleventh century AD) *On the Capacities of Foodstuffs*.

The non-medical manuscripts include a seventeenth-century *mathematarion* (MS.MSL.1), ie., an advanced textbook used by Greek students during the period of Ottoman rule (*Tourkokratia*).^12^ MS.413 is an illustrated collection of Byzantine oracles. Lastly, MS.498 presents late antique and Byzantine astronomical texts, together with some autograph religious poems from the late fifteenth century.

The Greek manuscripts of the London Medical Society have been described three times: by Charles Victor Daremberg (1817–72), by Joseph Baldwin Nias (1856–1919) and by Warren Royal Dawson (1888–1968). Those manuscripts acquired by the Wellcome Library separately between 1910 and 1936 were catalogued by its librarian Samuel Arthur Joseph Moorat (1892–1975). The majority are described below in full detail. MSS MSL 112, 124 and 126, however, have only been given summary descriptions, since they are relatively recent copies, made in England, of Greek texts found in earlier codices. I hope to assist future scholarly research by providing descriptions that are more detailed than the ones heretofore available.

## 2. Note on Contents and Bibliography

The **heading** of each catalogue entry is organised as follows: [current and former shelfmarks of the manuscript], [its summary contents], [place of origin], [date], [writing material], [number of folia, ie. leaves], [height and width of leaves], [number of lines per page], [justification, ie. height and width of the written area], [ruling type]. All **measurements** are given in millimetres. Types of **ruling** are identified according to Jacques-Hubert Sautel (ed.), *Répertoire de réglures dans les manuscrits grecs sur parchemin* (Turnhout: Brepols, 1995) [*Bibliologia*, 13]. The ruling is always, unless otherwise specified, in drypoint. The location of **quire signatures** is described as follows: 
(upra) – top of the page, 
(nfra) – bottom of the page;  – internal page corner, 
– mid-margin,  – external page corner; *1* – first page of the quire, *2* – first and last pages of the quire, *3* – last page of the quire. **Titles** are given in bold; inc(ipit) and des(init) refer respectively to the opening and ending phrase of a certain work or of a section of a certain work. As a large number of the medical texts do not have a critical edition, in addition to beginning and ending I give a detailed account of the table of contents (where available), because this may be useful for determining the place of a codex within the tradition of a given text. **Transcriptions** from Greek are diplomatic and retain the spelling and punctuation of the relevant codex. In those cases when a **printed edition** corresponds to a text found in one of the catalogued manuscript, the name of its editor is given in 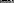, followed by the corresponding page and/or line numbers. (If the printed text differs somewhat from the one found in the MS, its editor’s name is preceded by the sign .) The full title of the edition can be found either in a footnote or in the bibliography at the end of the corresponding catalogue entry. If a text is included in the TLG database, I provide the relevant reference; the TLG version does not necessarily correspond to the version of the text as it stands in a certain manuscript. For each work, I provide the most common English title. Names of ancient and Byzantine authors follow *OCD* and *ODB*, respectively. I refer to texts in the Hippocratic Corpus as being by [Hippocrates]. I have employed the following **bibliographical abbreviations**:

*Bibl. Askev.*: *Bibliotheca Askeviana manu scripta, sive catalogus librorum manuscriptorum Antonii Askew* (London: Leigh & Sotheby, 1785)
*Cat. Med. Soc*. 1803: *A Catalogue of Books Contained in the Library of the Medical Society of London, Instituted AD 1773* (London: Medical Society of London, 1803)
*Cat. Med. Soc*. 1829: *A Catalogue of Books Contained in the Library of the Medical Society of London, Instituted AD 1773* (London: Medical Society of London, 1829)
*CMG*: *Corpus Medicorum Graecorum* (http://cmg.bbaw.de, accessed 18 December 2014)
Daremberg: Charles Victor Daremberg, *Notices et extraits des manuscrits médicaux grecs, latins et français des principales bibliothèques de l’Europe* (Paris: J.-B.Baillière, 1853)
Dawson: Warren Royal Dawson, *Manuscripta Medica: A Descriptive Catalogue of the Manuscripts in the Library of the Medical Society of London* (London: J. Bale, Sons & Danielsson, 1932)
Diels: Hermann Diels, *Die Handschriften der antiken Ärzte*, 3 vols (Berlin, 1905–8) [*Philosophische und historische Abhandlungen der Königlich Preussischen Akademie der Wissenschaften*, (1905) 3, (1906) 1, (1908)]
Moorat: Samuel Arthur Joseph Moorat, *Catalogue of Western Manuscripts on Medicine and Science in the Wellcome Historical Medical Library*, 3 vols (London: Wellcome Institute, 1962–73)
Nias: Joseph Baldwin Nias, ‘Special Report on the Greek MSS in the Society’s Library’, *Transactions of the Medical Society of London*, 27 (1905), lii–lvii
Nutton: Vivian Nutton, ‘The Legacy of Hippocrates: Greek Medicine in the Library of the Medical Society of London’, *Transactions of the Medical Society of London*, 103 (1986–7), 21–30
Nutton and Zipser: Vivian Nutton and Barbara Zipser, ‘A Wellcome Manuscript of a Medical Practitioner’, in Véronique Boudon-Millot *et al.* (eds), *Storia della tradizione e edizione dei medici greci: Atti del VI Colloquio Internazionale, Paris 12–14 Aprile 2008* (Naples: D’Auria, 2010), 259–70
*OCD*: Simon Hornblower, Anthony Spawforth and Esther Eidinow (eds), *The Oxford Classical Dictionary*, 4th edn (Oxford: Oxford University Press, 2012)
*ODB*: Alexander Kazhdan (ed.), *The Oxford Dictionary of Byzantium*, 3 vols (Oxford: Oxford University Press, 1991)
Piccard: *Wasserzeichenkartei Piccard* (http://www.piccard-online.de, accessed 18 December 2014)
*RGK*: Herbert Hunger (ed.), *Repertorium der griechischen Kopisten, 800–1600*, 3 vols in 9 pts. (Vienna: Verlag der Österreichischen Akademie der Wissenschaften, 1981–97) [cited with volume no. followed by catalogue no., eg. *RGK*I 213]
TLG: *Thesaurus Linguae Graecae* (http://www.tlg.uci.edu, accessed 18 December 2014)
Touwaide: Alain Touwaide, ‘Byzantine Medical Manuscripts: Towards a New Catalogue, with a Specimen for an Annotated Checklist of Manuscripts Based on an Index of Diels’ Catalogue’, *Byzantion*, 79 (2009), 453–595

## 3. MS.MSL.1 (*olim* HH i 16 / We 15)

Ottoman Empire, ca. 1620–1640 AD (from watermarks).

Paper, 207  147, iii  199  iv (foliated 1 [flyleaf iii]-200), linn. 22 [ca. 157  100], unruled.

*Mathematarion* **:** **[2r–163v]** Anonymous commentary on Aristotle’s *Categories*.^13^ **[166r–174r]** Anonymous collection of brief theological texts. **[175r–200v]** Anonymous commentary on Aristotle’s *Prior and Posterior Analytics.*

**Text: [2r–6r]** **῾Ερμηνεία εἰς τὰς Αριστοτέλ(ους) κατηγορί(ας).** **Προοίμιον**, inc. Μετὰ τ(ὴν) τοῦ Πορφυρίου εἰσαγωγὴν (καὶ) ἐπ᾿ αὐτὰς τὰς τοῦ ᾿Αριστοτέλους κατηγορί(ας), des. οφελούντ(ων) πρὸ(ς) τ(ὰς) τῶν κατηγοριῶν διδασκαλί(ας), ἤδη λοιπὸν (καὶ) ἐπ᾿ αὐτὰς ξὺν Θεῷ μεταβῶμ(εν). **[6v–56r]** **᾿Αρχὴ τοῦ κειμένου τ(ῶν) κατηγοριῶν**, inc. ῾Ομώνυμα λέγεται ὧν ὄνομα μόνον κοινόν, ὁ δὲ κατὰ τοὔνομα λόγος τ(ῆς) οὐσίας ἕτερος, des. εἰς ἀπόκρισιν (καὶ) λύσιν τῶν εἰρημένων ἀποριῶν. **[58r–69v]** **Περὶ τοῦ ποσοῦ**, inc. Τοῦ ποσοῦ τὸ μέν ἐστι διωρισμένον, τὸ (δὲ) συνεχὲς: Μετὰ τὴν τ(ῆς) οὐσί(ας) διδασκαλί(αν), des. συντομί(ας) ὢν ἐραστὴς ἕκαστον οὐκ ἀπηρηθμήσατο. **[70r–84v]** **Περὶ τῶν πρός τι**, inc. Πρός τι δὲ τὰ τοιαῦτα λέγεται, ὅσα αὐτὰ ἅπερ ἐστι ἑτέρ(ων) εἶναι λέγεται: οὐ μικρά τις διαμφισβήτη ἔρις[?] πρό(ς) τ(ῶν) ἑρμηνευτῶν (καὶ) περὶ ταύτην φέρεται τ(ὴν) κατηγορί(αν), des. κ(αὶ) ταῦτα ὅσον κ(αὶ) ην εἰς τ(ὴν) τοῦ κειμένου ἑρμηνείαν.**[86r–100v]** **Περὶ ποιοῦ (καὶ) ποιότητος**, inc. Ποιότητα δὲ λέγω, καθ᾿ ἢν ποιοί τινες ἐ(στι) λέγονται: τίνος ἕνεκεν τ(ὴν) ποιότηταν τ(ῶν) λοιπ(ῶν) κατηγοριῶν, des. ἐν τῶ ἐνηρημένῳ δύναντ(αι) ὥσπερ ἐν οἰαδήποτε εἴδει ληφθῆναι. **[101r–121r]** **Περὶ τοῦ ποιεῖν (καὶ) πάσχειν, (καὶ) τῶν λοιπῶν κατηγορι(ῶν)**, inc. ᾿Επιδέχετ(αι) (δὲ) (καὶ) τὸ ποιεῖν (καὶ) πάσχειν ἐναντιότητα, des. δῆλον (ἐστι) ἐν τῇ τοῦ προτέρου διδασκαλίᾳ συνέλαβε (καὶ) τ(ὴν) τοῦ ὑστέρου. **[122r–129r]** **Περὶ τοῦ ἅμα**, inc. ῞Αμα δὲ λέγεται, ἁπλῶς (καὶ) κυριώτατα, ὧν ἡ γένεσις ἐν τῷ αὐτῶ χρόνω· οὐδέτερον γὰρ τῶν τοιούτ(ων), des. ἐν αἷς κ(αὶ) τὸ τέλος ξὺν Θεῷ τὸ τοιοῦτον ἐπιτεθήκαμεν. **[130r–143r]** **Περὶ τ(ῆς) τοῦ γένους διαιρέσεως, ἀπορίαι τέτταραις**, inc. ᾿Επὶ τοῦ τηθέντος ὁρισμοῦ τοῦ γέν(ους), des. τὸ τρίτον οὐκ ἰδίως ἀθροιστικόν, ἀλλὰ καθόλου λύετ(αι). **[143v–149r]** **Ζητήματα ἢ ἀπορρίαι γνώσε(ως) ἄξια ἐν τῷ τ(ῆς) διαφορᾶς κ(ε)φ(αλαίῳ)· (καὶ) μάλιστ(α) περὶ τ(ὸν) αὐτοῦ ὁρισμὸν, μὴ εἶναι ὀρθῶς ἀποδεδομένον κ(α)τ(ὰ) πολλούς**, inc. ᾿Εν τῷ παρόντι κ(ε)φ(αλαίῳ) εἰσὶ τινες ἀπορίαι μετὰ τῶν αὐτ(ῶν) λύσεων, αἵτινες παρ᾿ ἡμῖν διὰ βραχέ(ων) διακρίνονται, des. ἃς μακρὰν ἀναγγέλειν εἶναι πεφύκασι. **[149v–150v]** **Περὶ τοῦ ἰδίου ᾿Απορίαι τινές, γνώσε(ως) ἄξιαι ἐν τῷ τοῦ ἰδίου κεφαλαίῳ**, inc. ᾿Εν τῷ παρόντι κ(ε)φ(αλαίῳ), ἐπορουέμένά τινα έστι, des. ὡς αὐτὸ τὸ εἶδος ὧ συνέρχετ(αι). **[150v–160r]** **Απορίαι τινὲς (καὶ) ζητήμ(α)τ(α) ἐν τῷ τοῦ συμβεβηκότος κ(ε)φ(αλαίῳ) (καὶ) τῶν τούτου ὁρισμῶν**, inc. Πάντα σχεδὸν τὰ ἐν τῷ τοῦ συμβεβηκότος, des. (καὶ) προμηθείᾳ ἐπισκεμμέν(ως) (δὲ) βραχὺ διεξέλθομεν. **[160r–163v]** **Απορίαι ἐν τῷ τοῦ Πορφυρίου προοιμίου**, inc. Τριχῶς ἦν ὁ διαλογισμός, des. ἆρα καθόλου ἐισὶ πράγματα, ἢ φωναὶ μόναι ἢ ἐπίνοιαι. **[166r]** no title, inc. Πολλὰ (καὶ) [μ]εγάλα κακὰ κάμνει ὁ ἕρως εἰς τοὺς ἀν(θρώπ)ους, (καὶ) τέτοι(ας) λογῆς, ὁποῦ σχεδὸν εἶναι ἀδιήγητα, des. ἐκαβαλίκευσε τὰ λεοντάρια· ἀπέχετε ἄν(θρωπ)οι ἀπὸ ἐτοῦτον. **[166r]** no title, inc. Οἷα δυνατοῖς ἀνθρώποις ὁ ἔρως ποιεῖ, des. τοῖς λέουσιν ἐπιβεβηκέναι· ἀπέχετε τούτου ὦ ἄν(θρωπ)οι. **[166v]** no title, inc. Γνωρίζοντας οἱ ἄν(θρωπ)οι τ(ὴν) ἀδυναμί(αν) τους, des. μὴ ἂν κολάζεται, τὸν ὑστερεῖ (καὶ) ἀπὸ ἐκεῖνα ὁποῦ ἔχει. **[166v]** no title, inc. Γινώσκοντες οἱ ἄνθρωποι τὸ ἀσθενὲς αὐτῶν, des. (καὶ) τὰ ὑπάρχοντα αὐτὸν ἁφερεῖ. **[166v]** no title, inc. ῍Αν σε διδάσκουν π(άτ)ερ, des. λύω, να συντεχένεις ὀλίγα. **[167r]** no title, inc. ῏Ω ἄνδρες, οἱ ὁποίοι βρύετε ψήρας (καὶ) ψύλας, des. (καὶ) δὲν γεννῶνται αἱ ψῆραι (καὶ) ψύλαι. **[167r]** no title, inc. ῏Ω ἄνδρες οἱ ψήραις (καὶ) ψύλαις, des. (καὶ) δὴ οὐ φύονται αὗται. **[167r]** no title, inc. Διά τι ὡς ἀντολογικὸς ὁ λόγος, des. ἀνάγκη εἶναι νὰ λαμβάνη ὕβριτ(ας) (καὶ) ξυλαῖς. **[167v]** no title, inc. Δὲν πρέπει ὁ ἀν(θρωπ)ος νὰ λυπεῖται, des. ἕως ὅπου νὰ τὰ θανατώσει διώκοντάς τα. **[167v]** no title, inc. Οὐ δεῖ λελυπεῖσθαι τ(ὸν) ἄνθρωπον ἐν οὐδενὶ δυστυχήματι, des. ἄχρις ἂν καταβάλη τὰς ταύτας ἐλαύνων. **[168r]** no title, inc. Εἰς κάθε τόπον οἱ ἄν(θρωπ)οι ἑορτάζουσι τὸν μέγαν Νικόλαον, des. ἀλλὰ ἐνταντίον (καὶ) ἐχθρὸν εἰς τ(ὴν) ψυχὴν τ(ούς). **[168r]** no title, inc. ᾿Εν παντὶ τόπῳ οἱ ἀν(θρωπ)οι τ(ὸν) μέγαν Νικόλαον ἑορτάζουσι, des. ἀλλ᾿ ἐναντίον κ(αὶ) ἐχθρὸν κατὰ τ(ῆς) ἑαυτῶν χῆς. **[168v]** no title, inc. Τὰ ξύλα ὁποῦ ἀνάπτονται εὔκολα, des. περισσότερον ἀπὸ κάθε λογῆς φωτίαν. **[168v]** no title, inc. Τὰ ὑπὸ τοῦ πυρὸς ῥαδίως ἁπτόμενα ξύλα, des. μᾶλλον ἁπάσης φλογός. **[168v]** no title, inc. ᾿Αφότις οἱ ἄν(θρωπ)οι ἄρχισ(αν) νὰ πονηρεύοντ(αι), des. (καὶ) πίπτει εἰς ἄλλον χειρότερον. **[169r]** no title, inc. Πολλάκις ἔγραψα ἀφθεντίας σας πῶς εὑρίσκομαι, des. ἂν κάμη (καὶ) ἄλλο τίποτες χρεία. **[169r]** no title, inc. Πολλάκις ἐπέστειλα πρὸς ἡμᾶς, des. χρεῶν εἴη ἕτερον, ἐπιστείλατε. **[169r]** no title, inc. ᾿Επειδήπερ ἡ ἁγία παρήχθη τεσσαρακοστή, des. δίκην χρυσοῦ καθαρωτ(ά)τ(ου) κ(αὶ) ἀκιβδήλου. **[169v]** no title, inc. ῎Ηθελα νὰ ἐγνωρίσ(ω) ἂν εἶμαι ἀγαπητός, des. ἂν δὲν κάμης τοιουτοτρόπως ἐσὺ ὄψει. **[169v]** no title, inc. ᾿Εβουλόμην μὲν οὐν εἰδέναι, des. μὴ οὕτως ποιήσης, αὐτὸς ὄψει. **[169v]** no title, inc. ᾿Εκεῖνος ὁ σπουδάζει εἰς τὴ φιλί(αν) τοῦ Θ(εο)ῦ, des. νὰ ἔχει ἐχθρὸν τὸν διάβολ(ον). **[169v]** no title, inc. ῾Ο τῇ φιλίᾳ σπουδάζων τοῦ Θ(εο)ῦ, des. ἐκείνον ἔχειν δεῖ ἐχθρὸν τ(ὸν) διάβολον. **[170r]** no title, inc. ῾Ας ἡμαστε καλὴ κ(αὶ) ἁς δίδομεν, des. διατὶ τὰ χρειάζεται. **[170r]** no title, inc. Καλῶς ἔχοιμεν (καὶ) διδῶμεν, des. (καὶ) γὰρ τούτων δεῖται. **[170r]** no title, inc. ᾿Επειδὴ ἔφθασεν ἡ ἁγία τεσσαρακοστή, des. τ(ὸν) ἀπρόσμενον καιρόν. **[170v]** no title, inc. ᾿Εκεῖ ὀποῦ τινὰς δύναται νὰ κάμη καλόν, des. (καὶ) ἱερὰ γράμματα. **[170v]** no title, inc. Οὗ μὲν τὶς εὖ ποιεῖν δύναται, des. τοῖς πολλοῖς γεγονέναι. **[170v]** no title, inc. Μὴν ἀμελοῦμεν λοιπὸν, des. τὸ καθαρὸν κ(αὶ) ἄδηλον. **[171r]** no title, inc. ῞Ενας τινὰς ἄν(θρωπ)ος ἐπιθύμυσε νὰ γένη ἀείδαρος, des. (καὶ) ἔγινε φαγιτὸν τῶν ὀρνέων. **[171r]** no title, inc. ῎Ονος ἐπεθύμησε τὶς γενέσθ(αι), des. κ(αὶ) βορὰ τοῖς ὀρνέοις γέγονε. **[171r]** no title, inc. ᾿Αφ᾿ οὖ ἤρξαντο πονηρεύεσθ(αι) οἱ ἄν(θρωπ)οι, des. εἰς ἕτερον τι ἐμπίπτει χαλεπώτερον. **[171v]** no title, inc. ῞Ολοι οἱ καλοὶ τ(ὸν) καιρὸν ἐτοῦτον, des. ἅπαντα ἐχουσι γένει ὅλοι καλοί. **[171v]** no title, inc. Οἱ χρηστοὶ τῶν ἀν(θρώπ)ων, ἐν τῷ παρόντι, des. εἶτα χρηστοὶ γενήσονται ἅπαντες. **[171v–172r]** no title, inc. Οἱ πτωχοὶ, ἑστοντας νὰ εἶναι ἐνδεδυμένοι, des. (καὶ) ὡσάννα[?] ἐπαραπονεῖτον εἰς τὸν Θεόν. **[172r]** no title, inc. Μέγα οἱ ἄνθρωποι ἄχθονται, des. ἐπὶ τῷ Θεῷ ἀγανακτῶν ἦν. **[172v]** no title, inc. ᾿Εκεῖνος ὁποῦ ὁμοιάζει τῶν ἀλόγων ζώ(ων), des. (καὶ) ἡ ἀλώπηξ εἶναι τέτοιας λογῆς. **[172v]** no title, inc. ῾Ο τ(ὴν) θέαν τοῖς ἀλόγοις ζώοις, des. ἡ ἀλώπηξ τοιαύτη ἐστί. **[172v]** no title, inc. Οἱ πένητες, ἀμπεχόμενοι τριβώνια, des. πάντως μετέχειν ἀνάγκη τῶν κακ(ῶν). **[173r]** no title, inc. ῞Οσοι γεννηθοῦσιν εἰς τὴν ἅραν τῆς ᾿Αφροδίτης, des. ὅτι ἐγὼ δὲν ἦμουν ἐκεῖ ὅταν ἐγίνονταν. **[173r]** no title, inc. Οἱ μ(ὲν) ἐπὶ τ(ὴν) τ(ῆς) ᾿Αφροδίτης ἅραν τεχθέντες, des. ἡνίκα τᾶυτα ἐγένετο. **[173r]** no title, inc. Εἰ καθεκάστην π(άτ)ερ ἅγιε παραινοῦσι, des. φύλαξαι, τὸ βραχέα λέγειν φημί. **[173v]** no title, inc. Τοὺς φιλονίκους (καὶ) ἐκεῖνους ὁποῦ λογιάζουσι, des. διωχθῆτε ἀπὸ κάθε λογῆς συντροφίαν. **[173v]** no title, inc. Τοὺς ἐρίζοντ(ας) (καὶ) ἐαυτοὺς, des. ἵνα μὴ πάσης ὁμιλί(ας) ἀποπεμθῆται. **[174r]** no title, inc. Τότε (δὲ) ἀνταίρει τό ἐπιχείρημα  τῆς αἰτίας· ἐπεὶ πλείω, des. ἡ φθορὰ (καὶ) στὰ ὅμοια. **[174r]** no title, inc. Τοῦτο (δὲ) ἀποκρίνεται ὁ ᾿Αλβέρτος, des. τῶν τοιούτ(ων) προθε ἀντιθέσεων. **[174r]** no title, inc. Οὗ ἕνεκεν οὕτως μοι δοκοῦ λύεσθ(αι), des. ὅμως ἡ θερμότητα αὐτοῦ προσιοῦσα οὐ φθέρει τ(ὸν) ἄνθρωπον.**[175r–177v]** **Περὶ τ(ῶν) τ(ῆς) οὐσί(ας) ἰδιωμάτ(ων) ἀπορίαι τινὲς (καὶ) ζητήματ(α)**, inc. ᾿Επὶ τὰ ἐξ τῆς οὐσίας ἰδιώμ(α)τ(α), ἤτοι ἴδια, des ὃς οὐδαμῶς ἔγνω τὸ τοιοῦτον. **[178r–179r]** **Περὶ** **εἰς τὸ πρῶτον τ(ῶν) προτέρ(ων)**, inc. Τὸ προοίμιον, σύντομον ἐνταῦθα κ(αὶ) σαφὲς ὁ ᾿Αριστοτέλης ἐξέθηκε λέγων, des. ἀνάγκη ἐπαναλαμβάνειν αὖθις. **[180r–183r]** **Κεφ(άλαιον) Β΄**, inc. Μετὰ τὸ ἐκθέσθ(αι) τ(ὸν) φιλόσοφον τὰς τῶν προτάσεων, des. ἡ φρόνησις ἄρα ἔστιν ἀγαθόν. **[184r–185v]** **Περὶ τοῦ γ΄ κεφαλέ^ου^**, inc. Τὸ παρὸν κεφάλεον, εἰς τρία ὁ ᾿Αρ(ιστοτέλης) διαιρεῖ μέρη, des. ἄρ᾿ οὐ πᾶν λευκὸν ἐστ(ι) ἄν(θρωπ)ος. **[186r–188v]** **Κ(ε)φ(άλαι)^ον^ δ΄**, inc. Μετὰ τ(ὸν) τῶν δύο τοῦ συλλογισμοῦ, des. ὅλως γενέσθ(αι) καθόλου συμπέρασμα. **[189r–191v]** **Κ(ε)φ(άλαι)^ον^ Ε΄**, inc. ᾿Εν τῷ παρόντι κεφαλαίῳ τρία τινὰ παραδίδωσι, des. ἄν(θρωπ)ος ἐργάζεται: οὐκ ἐργάζεται. **[192r]** **Περὶ τοῦ στ΄ κ(ε)φ(αλαί)^ου^**, inc. ᾿Επεὶ δ᾿ ἕτερον ἐστ(ι) ὑπάρχειν, des. οὐ γίνεται τέλειος συλλογισμός. **[193r–v]** **Κ(ε)φ(άλαιον) ζ΄**, inc. Συμβαίνει δὲ ποτε, (καὶ) τ(ῆς) ἑτέρ(ας) προτάσεως, des. τὸ λευκὸν ὑπὸ τὸ ζῶον ἐστὶν ἐξ᾿ ἀνάγκης. **[194r–195v]** **Περὶ εἰκότος (καὶ) σημείου**, inc. Εἰκὸς (δὲ) κ(αὶ) σημεῖον, des. πρὸ(ς) φωκεῖς ἔ(στιν) κακός: (καὶ) τοῦτο. **[196r–197r]** **Περὶ ἐπαγωγῆς**, inc. ᾿Επαγωγὴ ἐστὶν, ὅταν τὸ πρῶτον, des. ἄρα κ(αὶ) κακία ἐστι ψεκτόν· ὅτι αἰσχρά. **[197v–199v]** **Περὶ ἐνστάσεως**, inc. ῎Ενστασις δὲ ἐστὶ πρότασις, des. λευκὸν οὐκ ἔστιν ἄν(θρωπ)ος. **[200r–v]** **᾿Εν δὲ τῷ τέλευταίῳ σχήματι**, inc. ᾿Επομένως τὰς τοῦ τρίτου σχήματος παραδέδωσι μίξεις, des. ἄρα οὐ πᾶν ζῶ(ον) ἐστὶν ἀγαθόν.

**Note:** (194v, 197r) the text is written upside down.

**Blank pages:** 56v–57v, 85r–v, 121v, 129v, 164r–165v, 174v, 179v, 183v, 192v.

**Annotations** (scribal): (*passim*) text occasionally crossed out and sometimes corrected – (2r, 15r, 22r, 24r, 26r, 27v, 52r, 56r, 58r, 59r, 70r, 73v, 77v, 82v, 89v, 92r, 100r, 113r, 117v, 118r, 121r, 132v, 133v, 140r, 140v, 142v, 143v, 144v, 146r, 146v, 147r, 149r, 151v, 153r, 159r, 160r, 166r, 169r, 170r, 173v, 175r) text expanded or glossed with synonyms in the outer margin: eg. (58r) ‘μαΐω νν’, (59r) ‘ἰουνίω ν’, (70r) ‘ἰουνίω ιν’, (73v) ‘ἤτοι ἡ στάσις’, (108v) ‘ὅρος στερήσε(ως) / definitio privationis’ – (29r, 127r, 181r–200r) explanatory diagrams in the outer margin.

**Handwriting:** unidentified post-Byzantine hand.

**Annotations** (non-scribal): (83r) ‘cuium / πότερον’.

**Paper:** folded in 4°; chain distance 27 mm; watermarks: (ff. 2–165, 186–200) similar to Velkov *Arbalète* 20–21, attested, respectively, in 1630 and 1635 – (ff. 166–173) similar (excl. countermark) to Heawood 4 (anchor), attested in 1620 – (ff. 174–185) similar to Heawood 951 (cross), attested in 1618.^14^

**Quire signatures** (scribal): Greek numerals *Im3* α (185v), *Im2* β (186r, 193v); Arab numerals *Ie* in the middle of each quire, from *9* (5v–6r) through *25* (127v–128r).

**Quires:** 1 (flyleaf iii), 2 (f. 2), 4 (f. 6), 6  8 (f. 53), 4 (f. 57), 12  8 (f. 161), 4 (f. 165), 8 (f. 173), 4 (f. 177), 2  8 (f. 193), 7 (f. 200), 3 (back flyleaves i–iii).

**Binding:** *corio russico*, same tooling as Wellcome MS.MSL.60. – Flyleaves i–1 and 201–203: chain distance 25 mm, no watermarks.

**Marks of ownership:** (front flyleaf II) ‘H H i 16’ – (1r) ‘Varii in Aristotelem’ – (back pastedown) label inscribed ‘L.g.11’.

**Provenance:** Anthony Askew (1722–74), London; [his sale, G. Leigh and J. Sotheby, London, 16 March 1785, lot 604]; purchased by James Sims (1741–1820), London, in 1785; purchased by the London Medical Society in 1802; purchased by the Wellcome Library in 1984.

Bibliography: *Bibl. Askev.*, 39 (no. 604); *Cat. Med. Soc*. 1803, 210; *Cat. Med. Soc*. 1829, 323; Nias, liv; Dawson, 7.
Roxane D. Argyropoulos and Iannis Caras, *Inventaire des manuscrits grecs d’Aristote et de ses commentateurs: contribution à l’histoire du texte d’Aristote. Supplément* (Paris: Les Belles Lettres, 1980), 33 (no. 214).

## 4. MS.MSL.14 (*olim* AA a 1 / Xa 32)

Possibly Cyprus (according to Barbara Zipser and Agamemnon Tselikas), fourteenth century.

Paper, 143  103, iii  157  iii (paginated 1–71, 73–131, 131–189, 191–232, 234–257, 259–317).

**[1–12]** Ps.-Hippocrates, *Epistle to King Ptolemy on the Constitution of Man* (TLG 0751.002). **[12–14]** Alexander the Sophist or Ps.-Hippocrates, *On the Human Body and Birth*. **[14–16]** Anonymous opuscule on phlebotomy.^15^ **[17–18]** Anonymous opuscule on conception.^16^ **[18–34]** Anonymous collection of remedies.^17^ **[34–41]** Ps.-Hippocrates, *Sayings about Life and Death*. **[41–44]** Anonymous collection of remedies.^18^ **[44–46]** Ps.-Esdras, *On Illuminating Days.* ^19^ **[46–76]** Anonymous collection of recipes on composite drugs. **[76–81]** Excerpt from an abridged edition of the vernacular version of John Archiatros’ *iatrosophion*. **[84–107]** Anonymous collection of remedies.^20^ **[82–83, 108–271]** Incomplete vernacular version of John Archiatros’ *iatrosophion.* ^21^ **[272–317]** Anonymous medical compilation.^22^

**Note:** This MS consists of two distinct parts, described below separately as **A** and **B**.

**A.**

ca. 1350 (from watermarks).

Linn. 18 [ca. 113  72], unruled.

**Original order of leaves:** pp. 1–81, 84–119, 122, 123, 120, 121, 126, 127, 124, 125, 128–271, 82, 83.

**Text: [1–12]** **᾿Επιστολὴ ᾿Ιπποκράτους· πρὸς τ(ὸν) Πτολαιμέων βασιλαία περὶ καταστάσε(ως) ἀν(θρώπο)[υ]**, inc. Συνέστηκ(εν) ὁ κόσμος ἐκ τεσσάρων στοιχίων, des. ὦ βασιλεῦ, ζήσει ἀλύπως (καὶ) ἀπόν(ως) τὸν ἐπίλοιπον χρόνον· τῆς δὲ διαιρέσε(ως) ταύτης· τοῦτον ἔχουσης τὸν τρόπον, τότε συνάγεται ὁ ἐνιαυτὸς εἰς ἡμέρας τξε΄ 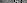. **[12–14–16]** no title, inc. ᾿Αλλὰ μηδεὶς ὑπολαμβανέτω, παντελὼς ἄψυχον εἶναι τὴν γονήν, des. τὸ δὲ θήλυ, μίας λειπούσης ἡμέρας ἐκ τῶν θ΄ μηνῶν, ἀποπνίσκεται, ἐπὶ μαρτίου· ἢ ἀπριλλίου· ἢ μαΐου· ἢ ἰουνίου· 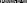. No title, inc. ῍Αν φλεβοτομήσει τις (καὶ) ῥεύσει αἷμα, des. ἔχων λωρία ὀστρακώδη, ἐξαμηνιαῖαν ζωὴν δηλοῖ 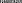. **[17–18]** no title, inc. Τὸ μ(ὲν) σπέρμα, ἐν τῇ μήτρᾳ προσερχόμενον, des. ὑπάρχωσι κομάτια καὶ μέρη, διακόσια εἴκοσι ἐννέα. **[18–34]** Περὶ φλεβοτομίας, inc. ῾Η χεὶρ ἡ δεξιὰ ἐὰν φλεβοτομεῖται εἰς τὸ καθολικόν, des. δὸς πιεῖν μετὰ οἴνου τῷ τετρωμένῳ (καὶ) θαυμάσεις. **[34–41–44]** **<Σ>ημείωσις ῾Ιπποκράτ(ους)· περὶ ζωῆς καὶ θανάτου**, inc. ῾Ο ἐμπειρικώτ(α)τος ἁπάντων τῶν ἀν(θρώπ)ων καὶ οἰκεῖα σοφία τῶν ἀν(θρωπ)ίνων σωμάτων ἐπιμελούμενος κεκελεύκει ῾Ιπποκράτης, des. ἢ πολὺ σύελος συνεχῶς ἐκχέει· εἰς κ΄· ἡμέρας ἀποθανεῖται 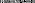. No title, inc. Θεραπεία εἰς βιασμ(ούς)· κώδιον (καὶ) ποτηροκλάστ(ας)· σὺν ταῖς ρίζες γλίχωνος, des. τοιαύτη γὰρ δύναμιν ἔχη τὸ ἐπίγγραμα τοῦτο. **[44–46]** **῎Εσδρα ἱερέ(ως) τοῦ προφήτ(ου) περὶ τῶν ἡμερῶν τῶν πεφωτισμένων καὶ ἀφωτίστων**, inc. Εἰσὶν αἱ ἡμέραι αὖται ἃς ἐφανέρωσ(εν), des. ἡ κδ΄· (καὶ) ἡ κε΄. **[46–47]** Table of Contents: no title, inc. Σκευασία α΄· ἡ ἱερὰ τοῦ Γαληνοῦ, des. Σκευασία κγ΄· τὸ διὰ τριῶν πεπέρεων· (blank space) σκύλης ὀπτῆς ἢγουν τὸ σκυλοκρόμμυον. **[47–54]** no title, inc. Σκευασία ἡ ἱερὰ τοῦ Γαληνοῦ α΄, des. μέλιτος τὸ ἄρκοῦν. **[54–55]** Table of contents: **Σκευασίαι αἱ διὰ τῶν ἐμπλάστρων**, inc. Σκευασία ἡ τοῦ εὐώδες τοῦ μεγάλου, des. Σκευασία ἡ τῆς ἴσιδος. **[55–76–81]** no title, inc. Σκευασία τῆς θείας, des. καστορίου στγ΄ β΄ · μέλιτος. No title, inc. Εἰς ζέσιν κεφαλῆς· το δέλαιον βάλε (καὶ) ὀξύδην, des. περὶ φλεγμονὴν ἥπατος τὸ λεγόμενον συκότην [οὕ]τος δὲ θέλεις νοησειν ὅτι 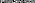. **[82–83]** no title, inc. ῞Οταν ὁ ἄν(θρωπ)ος ἔχη στρόφ(ους), des. ῥίζαν συκέα<ς> <κο>πάνισον· καὶ σμίξον με τὸ 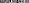. **[84–107]** no title, inc. [Με]θ᾿ ὕδατος ψυχροῦ κυάθ(ους) δύο, des. βάλον εἰς καλάμιν ἕ(ως) οὗ ἀποθάνουσιν τρίψας μετά. **[108–119]** Table of Contents: no title, inc. Νεφροὶ λίθ(ους), des. ὅταν θέλης να νοήσης παρθένον· πρὸς μὴ 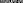. **[120–121]** no title, inc. ῎Αλειφε τὸ κεφάλην τοῦ ὅλον, des. τὰ τρίμματα (καὶ) ἔνωσέ τα· (καὶ) ἔπα[ρ(ον)] 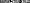. **[122–123]** no title, inc. Πρὸς ὀξὺν πὸνον κεφαλῆς, des. ῥοδέλαιον βάλλε· (καὶ) ὀξήδην· (καὶ) χλίανε τα· εἶ[τα] 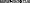. **[124–125]** no title, inc. [...]μα· καὶ δὸς τον ὀξύδην ὀλίγον, des. καὶ σακελλίσας αὐτά, βρέξε σπογγά[...] 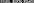. **[126–127]** no title, inc. Καὶ ἄλειψον τὸ μέτωπον, des. καὶ τότε ἔκβαλε τὸ κατάπλ[ασμα] 
Zipser, 179.3–180.15. **[128–271]** no title, inc. [Τ]ούτων, σμῖξον με τὸ μέλι (καὶ) στάξαι εἰς ὠτίον, des. δωλάκιν [καὶ μελάν]θιν τρίψα[ς] καὶ ὑψηλόν 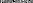.^23^

**Handwriting:** identified by Agamemnon Tselikas as a fourteenth-century Cypriot hand.

**Annotations** (non-scribal)**:** (36, 37, 38, 39) Latin marginal notes in Gothic script, indicating contents, eg. (38) ‘colia’, ‘dolor cor’ – (12) title added: **Τοῦ αὐτοῦ ῾Ιπποκράτους περὶ τοῦ σώματ(ος) (καὶ) διατυπώσε(ως) τοῦ ἀν(θρώπ)ου**.

**Paper:** folded in 8°; chain lines unclear; watermarks very similar to Mošin and Traljić 1900 (*cercle*), attested in 1352.^24^

**B.**

ca. 1320–1340 (based on the watermarks and the attribution of the handwriting).

Linn. 11 [ca. 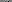], unruled.

**Original order of leaves:** pp. 272–279, gap, 282–283, 280–281, gap, 284–289, 292–293, 290–291, 294–297, gap, 298–305, gap, 306–317.

**Note:** pp. 284–317 have non-scribal Greek numbering in the lower margin; many leaves seem to be missing, so that, for example, λα (296) is followed by μβ (298).

**Text:** ^25^ **[272–274]** no title, inc. [Πίν]ε οἶνον εὔκρατον, des. ἀπὸ μελαίνας (καὶ) εχει φόβον 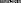. **[274–276]** no title, inc. Περὶ τοῦ γνωρίσης σφ[ι]γμὸν ἀρρώστου, des. οὔτε ζωὴν, οὔτε θάνατον 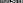. **[276–317]** no title, inc. ῎Αν ἐχει ἄν(θρωπ)ος κακὸν πυρετὸν, des. (καὶ) ἄλιφε τὴν κεφαλήν.^26^

**Handwriting:** very similar (according to Barbara Zipser) to that of Theodoulos Philagres, active in Cyprus in the early fourteenth century.^27^

**Text added** by later hands**:** (274–5) apotropaic formulae in lower margins, eg. ‘σοπι / πι / πι’^28^ – (275–86) remedies, including mostly Christians charms in invisible ink,^29^ eg. (279) ‘τοῦ υιοῦ καὶ τοῦ ἁγίου πν(εύματο)ς [...]ωγας [τ]ης παναγνης τζημπ[...] καρδίαν τὸν δοῦλον τοῦ Θ(εο)ῦ᾿ – (280) short cryptographic note – (278) fragmentary therapeutic advice: οστά τον φήνηκον υς κρατησην νερού οφελούσην.

**Paper:** folded in 8°; chain lines unclear; watermarks (1) possibly similar to Mošin and Traljić 4642–4727 (*hache*) and (2) very similar to Mošin and Traljić 2803 (*cloche*), attested in 1336–9.^30^

**A and B.**

**Quires:** 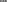 (p. 95), 4 (p. 105), 1 (p. 107), 6 (p. 119), 4 (p. 127), 6 (p. 139), 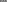 (p. 155), 6 (p. 167), 8 (p. 183), 7 (p. 213, no text lost), 8, 6, 8, 6; 6, 7, 4, 6.

**Binding:** *corio russico*, same tooling as the covers of London, British Library, Add. MS 5119. – Flyleaves: chain distance 22 mm; watermark (only upper half preserved) similar to Gravell and Miller 476 (horn), attested in 1779.^31^

**Marks of ownership:** (front pastedown) label inscribed ‘D.a.11’– (front flyleaf IV) ‘vid(e) Fabricii Bibl(iotheca) Graec(a) Vol. 12. p. 781 [ubi] mentionem facit primae Epistolae huius codicis’ – (front flyleaf IV) ‘a a / a / I’.

**Provenance**: Anthony Askew (1722–74), London; [his sale, G. Leigh and J. Sotheby, London, 15 March 1785, lot 578]; purchased by James Sims (1741–1820), London, in 1785; purchased by the London Medical Society in 1802; purchased by the Wellcome Library in 1984.

Bibliography: *Bibl. Askev.,* 36 (no. 578); *Cat. Med. Soc*. 1803, 94; *Med. Soc*. 1829, 158; Nias, lv; Diels, I.40–41, 47; Dawson, 24; Weinstock, *op. cit*. (note 48), 33 (no. 55); Nutton, 26; Jouanna, *op. cit*. (note 15), 41; Zipser, *op. cit*. (note 26), 129–34; Touwaide, 538–9; Zipser, *op. cit*. (note 21), 15–7; Nutton and Zipser, 259–70; Zipser, *op. cit*. (note 22), 251–68.

## 5. MS.MSL.52 A and B (*olim* HH i 21 and 22 / We 28 and 29)

Paper, 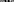, 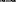 and 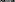 (foliated 1–202 and 203–282, 283, 284–292, 293–296, 297–306, 307–312, 312–321, 322–406).

**[1r–43v]** John Zacharias Aktouarios, *On the Activities and Illnesses of the Psychic Pneuma and the Corresponding Mode of Living* (TLG 3188.001). **[44r–143v]** *Idem*, *On Urines* (TLG 3188.002). **[145r–332v]** *Idem*, *Medical Epitome*,^32^ Books 1, 2 (TLG 3188.003), 3, first part of 6, 4, 5. **[333r–v]** Theophilos (Protospatharios) or Stephen, *On the Differences among the Fevers* (TLG 0746.001), excerpt on sweats. **[333v–341r]** Anonymus, *On Pulse*, in the form of questions and answers.^33^ **[341r–345v]** Paul of Aegina, *Medical Epitome* (TLG 0715.001), excerpt on pulse. **[345v–346r]** Short text on pulse. **[346r–v]** Aetios of Amida, *Tetrabiblon* (TLG 0718.005), excerpt on pulse. **[346v–354r]** Galen, *On the Pulse for Beginners* (TLG 0057.058), excerpt. **[354v–361v]** Theophilos (Protospatharios), *On Excrements* (TLG 0729.003). **[361v–363r]** Ps.-Hippocrates, *Sayings about Life and Death*. **[363v]** Anonymous collection of recipes for composite drugs. **[364r–366v]** Ps.-Galen, *On Procurable Remedies* (TLG 0530.029), excerpt. **[366v–403v]** Anonymus, *On Acute and Chronic Diseases* (TLG 0721.019).

**Note:** This MS consists of two volumes bound separately but foliated continuously. Volume 52B comprises two distinct parts, 52B1 and 52B2. 52A and 52B1 are the work of the same scribe and were originally bound together. These are described below under **I**, while 52B2 (ff. 333–403) is described under **II**.

**I.**

Constantinople, shortly before 1463 AD (from the note on f. 332v).

(Ff. 1–43) linn. 25 [ca. 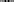], unclear ruling; (ff. 44–332) linn. 25 [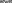], unclear ruling.

**Text: [1r–v]** Table of Contents: **Τοῦ σοφωτ(ά)τ(ου) (καὶ) λογιωτ(ά)τ(ου) κυρ(οῦ) ᾿Ιω(άνν)ου τοῦ Ζαχαρίου πρὸς τὸν φιλοσοφώτ(α)τ(ον) π(ατέ)ρα ὴμ(ῶν) κῦρ ᾿Ιωσήφ**, inc. Περὶ ἐνεργειῶν καὶ παθῶν τοῦ ψυχικοῦ πνέυματος καὶ τῆς κατ᾿ αὐτὸ διαίτης λόγος α^ος·^ ἐν ὧ εἰσὶ κε(φάλαι)α ταῦτα: ἀποδείξεις ἐκ τῶν κατὰ μέρος ἰδιοτήτων, des. περὶ αἰτί(ας) τῶν κατὰ τὸ δοξαστικ(ὸν) (καὶ) διανοητικ(ὸν) παθῶν 
Goupyl, iii–v.7. **[1v–22r]** no title, inc. ᾿Επειδὴ δέ σου ταῖς ἐνάγχος τε καὶ πρότριτα ἐπὶ φιλοσόφοις θεωρήμασι συνουσίαις, des. καὶ τ(ὸν) νοῦν ἡμῶν μετεωρισθήσεσθαι· καὶ ἐφέψεσθαι σοι χειραγωγούμενον 
Ideler, I.312–49. **[22r–v]** Table of Contents: **Τοῦ αὐτοῦ πρὸ(ς) τ(ὸν) αὐτὸν περὶ διαίτης ἐν ὧ εἰσὶ κε(φάλαι)α ταῦτα**, inc. Περὶ τῶν κατὰ τὴν πέψιν παθῶν: α^ον^ ΄, des. περὶ τοῦ πῶς ἂν ἕκαστον τῶν πνευμάτ(ων) ἐπιδιδοίη ἐκ τῆς προκειμένης μεθόδου· καὶ μάλιστα τὸ ψυχικ(ὸν) πνεῦμα 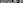. **[22v–43v]** no title, inc. ῾Οποῖόν τι ἐν ταῖς κατ(ὰ) τ(ὸν) βίον ἡμῶν χρήσεσι προδιανοούμενοι δρῶμ(εν), des. ἀποσβεννύμεν(ον) τὸ νοερὸν τὸ ἐν ἡμῖν (καὶ) καταπείσαις μεταδιώκειν τἀμείνωνα: τέλος 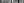. **[44r–v]** Table of Contents: **Τοῦ σοφωτ(ά)του (καὶ) λογιωτ(ά)τ(ου) πανσε(βάστ)ου σεβαστοῦ τοῦ ἀκτουαρίου κυροῦ ᾿Ιω(άννου) τοῦ Ζαχαρίου λόγος α**^**ος**^, inc. Τὰδε ἔνεισιν ἐν τῷ περὶ διαφορᾶς οὔρων λόγῳ· ὅτι χρήσιμος ἡ ἐκ τῶν οὔρων ἐπίσκεψις, des. περὶ τάξεως (καὶ) ἀταξί(ας) ἑκάστου τούτων 
Leone, xv–xvi; Georgiou, 398–400. **[44v–59v]** **Προοίμιον** **:** **α**^**ον**^ **΄**, inc. Πάλαι μ(ὲν) ἴσως φιλοτομί(ας) ἔργον τιθέμενος, des. ἀρχῆς τῶν μετὰ τοῦτον λόγων ἁψόμεθα 
Ideler, II.3–31.19; Georgiou, 401–56. **[59v–60r]** Table of Contents: **Τοῦ αὐτοῦ σοφωτ(ά)τ(ου) τοῦ ἀκτουαρίου περὶ οὔρ(ων) βιβλίον δεύτερον**, inc. Τὰδ᾿ ἔνεισιν ἐν τῷ πρώτῳ περὶ διαγνώσεως πὔρων λόγῳ· περὶ διαφορᾶς ἀμίδος, des. περὶ διαγνώσεως φαιῶν (καὶ) μελανῶν οὔρων 
Leone, 59.8–60; Mathys, I.161–162.4. **[60r–71r]** **Προοίμ(ιον)**, inc. ᾿Επειδὴ τὸ μανθάνειν ἐθέλειν τὰς ἐκ τ(ῶν) οὔρων σημειώσεως, des ἣν πάντα τρόπον ἀκριβῶς ἀσκεῖν προσήκει πειρᾶσθαι 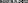. **[71v–72r]** Table of Contents: **Λόγος β^**ος**^**, inc. Τάδ᾿ ἔνεισιν ἐν τῷ δευτέρῳ περὶ διαγνώσεως οὔρων λόγῳ· περὶ τῆς ἐκ τῶν κατὰ τὰ οὖρα, des. ὅτι δεῖ τὸν ἀκριβῶς ἐπισκέπτεσθαι βουλόμενον (καὶ) τ(ὴν) περὶ σφυγμῶν πραγματεῖ(αν) ἀκριβ(ῶς) ἐπιστάσθαι 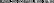.**[72r–86r]** **Περὶ διαγνώσεως οὔρων λόγῳ**, inc. Τὸ διαγινώσκειν τῶν παθῶν τὰ κατέχοντα, des. συμπεράνει λόγοις ἀληθεί(ας) πιστούμενα 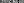. **[86r]** Table of Contents: **[Τ]άδε ἔνεισιν ἐν τῷ πρώτῳ αἰτί(ας) οὔρων λόγῳ**, inc. [Π]ερὶ αἰτί(ας) τῶν ζώων διαμονῆς, des. περὶ τῆς τῶν συμμεμιγμέν(ων), ἤτοι ἀνομοιομερῶν παρυφισταμένων αἰτίας 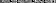. **[86r–103v]** no title, inc. [᾿Ε]πειδῆ τῷ περὶ τινος αἰρουμένω γράφειν ζητήματος, des. τοῖς δὲ σπουδάζουσιν, οὐκ ἀγεννὴς ἔσται μέθοδος 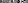. **[103v]** Table of Contents: **[Τ]άδε ἔν ἐστιν ἐν τῷ δευτέρῳ περὶ αἰτί(ας) οὔρων λόγῳ**, inc. [Π]ερὶ αἰτί(ας) λείων (καὶ) τραχεί(ων) (καὶ) γλισχρ(ῶν) παρυφισταμέν(ων), des. [π]ερὶ αἰτί(ας) οὔρων τῶν κατ(ὰ) ψυχρὰν διάθεσιν καιρί(ων) πεπονθότων μορί(ων) 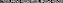. **[103v–119r]** no title, inc. [Ο]ιόν τι τοῖς φιλοθεάμοσι συμβαίνειν εἴωθεν, des. ἤδη καὶ τοῦ περὶ προγνώσεων οὔρων 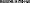. **[119v]** Table of Contents: **[Τ]άδε ἔνεισιν ἐν τῷ πρῶτῳ περὶ προγνώσεως οὔρων λόγῳ**, inc. [῞Ο]τι μετὰ τ(οὺς) σφυγμοὺς τὰ οὖρα συντείνει εἰς πρόγνωσιν, des. [π]ερὶ προγνώσεως τ(ῶν) ἐκ τῶν κατὰ τὴν στεφάνην ἑτεροειδῶν χρωμάτ(ων) 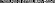.**[119v–132v]** no title, inc. ᾿Εδοκει τισὶ τῶν τὰς προγνώσεις διαβάλλειν προχείρ(ων), des. ὑγιαίνουσι τῆς πραγματεί(ας) σκοπήσας χρήσιμον 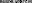. **[132v]** Table of Contents: **[Τ]άδε ἔνεστιν ἐν τῷ περὶ προγνώσεως οὔρων δευτέρῳ λόγῳ**, inc. [Π]ερὶ τῆς ἐκ τῶν ἀνυποστάτ(ων) οὔρ(ων) προγνώσε(ως), des. [ἐ]πίλογος ἐν ὥ (καὶ) τις ἀξίωσις πρὸ(ς) τ(οὺς) ἐντυγχάνοντας τῇ πραγματείᾳ ταύτῃ 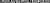. **[133r–143v]** no title, inc. [Τ]ὸ τὴν πρόγνωσιν ἀσκεῖν τε καὶ ἐπιτηδεύειν, des. αὐτῶν δὲ τῶν λόγων ἀκριβής τις ἐπίσκεψις: 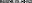. **[145r–178r]** **Περὶ διαγνώσεως καὶ αἰτιῶν κ(α)τ(ὰ) μέρος παθῶν· λόγος α**^**ος΄**^ **· τοῦ αὐτοῦ σοφωτ(ά)τ(ου) ἰατροῦ ἀκτουαρίου κυροῦ ᾿Ιωά(ννου) τοῦ Ζαχαρίου· θεραπευτικ(ῆς) μεθόδου, βιβλίον, πρῶτον**, inc. ᾿Επειδή σοι εἰς τὴν ὑπὲρ τοῦ γένους ἡμῶν στελλομένῳ πρεσβεί(αν) ἐπὶ τ(οὺς) ὑπερβορείους Σκύθας, des. τὸ δὲ νῦν ἔχον, ἐ᾿π ἄλλα τὴν σπουδὴν τρέψωμ(εν) 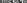. **[178r–202v]** **Τοῦ αὐτοῦ σοφωτ(ά)τ(ου) ἰατροῦ του ἀκτουαρίου θεραπευτικ(ῆς) μεθόδου, βιβλίον, β**^**ον**^ **΄**, inc. Οὐ κατ᾿ ἐκείν(ους) τῶν φίλων ἡμεῖς, οἳ παρόντες μ(ὲν) οἷς ἂν φιλίαν, des. εἴη δὲ σὲ μ(ὲν) ἀπόνασθαι τοῦ γράμματος· ἡμᾶς δὲ λέγειν τὰ δέοντα 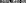. **[203r–224r]** **Περὶ θεραπευτικ(ῶν) μεθόδ(ων) βιβλίον πρῶτον**, inc. ᾿Επειδὴ πᾶσα διδασκαλία (καὶ) μέθοδος, des. τὸ πᾶν τοῦ λόγου μέτρον συμπεραντέον 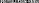. **[224r–257v]** **Τοῦ αὐτοῦ περὶ θεραπεί(ας) παθῶν (καὶ) τῶν ἔξωθ(εν) φαρμάκ(ων)**, inc. ᾿Εδόκει μοι διὰ βράχε(ων) πάντων ἐπιμνησθῆναι βεβουλημένῳ, des. ὡς ἂν ἐν πᾶσι Θ(εο)ῦ διδόντος, ἄρτιος ὁ λόγος τελοίη 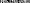. **[257v–295v]** **Τοῦ αὐτοῦ περὶ θεραπευτικ(ῆς) μεθόδου τῶν κατὰ μέρος παθ(ῶν) βιβλίον δεύτερον**, inc. ῾Η μ(ὲν) παροιμία φησί, χελώνης κρέα, ἢ φαγεῖν, ἢ μὴ φαγεῖν, des. προσῆκον ὠδὶ καὶ τοῦτον ἐνταῦθά πη συγκαταπαύειν 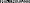. **[296r–332v]** **Τοῦ αὐτοῦ περὶ συνθέσε(ως) φαρμάκ(ων) λόγος α**^**ος**^ **΄**, inc. ῎Ηδη σοι καὶ τ(ὸν) ἐπὶ τοῖς τέσσαρσι βιβλίοις ἐπιτίθεμ(εν) λόγον, des. μετὰ τοῦτον δὲ προσθήσομ(εν) ὅσα δοκεῖ λείπειν πρὸ(ς) τὸ τῆς ὑποσχέσεως ἄρτιον 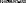. **[332v]** Historical note: inc. Κ(α)τ(ὰ) τὴν κς΄ τοῦ Μαρτ(ίου) μηνὸς, des. νυκτ(ὸς) ὥρ(ᾳ) δ΄ τοῦ ͵ςϡοβ΄ ἰν(δικτιῶνος) ια΄ 
Powell, 359–60; Gamillscheg, 297–8; cf. Gehin and Kouroupou, I.410–11 (no. 172).^34^

**Annotations** (possibly scribal)**:** (32v, 34r, 46v, 47r, 49v, 50v, 51v, 54r, 56v, 59r, 63r, 65v, 66v, 70r, 74v, 76v, 78v, 80v, 85r, 85v, 88v, 93r, 93v, 95v, 96r, 96v, 98r, 99v, 100r, 100v, 103r, 105r, 108r, 109v, 110v, 112v, 114r, 115v, 116r, 116v, 117v, 118r, 118v, 119r, 119v, 120r, 121r, 121v, 124v, 125r, 125v, 126r, 126v, 127r, 129r, 130r, 125r, 135r, 136r, 136v, 137r, 140r, 140v, 141v, 143r, 143v 145r, 146v, 147r, 150v, 151r, 152v, 154v, 155r, 155v, 157v, 158r, 158v, 159v, 160v, 161r, 162v, 164r, 165r, 169r, 169v, 174v, 176v, 177r, 177v, 181v, 182v, 186r, 187r, 192v, 193v, 195v, 196r, 198r, 199v, 201r, 202v, 203r, 204r, 205r, 206r, 206v, 209v, 207v, 220v, 256v, 269v, 273v, 282bis v, 307bis r, 313v, 321bis r) correction or additions to the main text, variant readings, short explanations or indications of contents, eg. (32v) ‘μαλακόστρακα ἤτοι οἱ ἀστακοὶ (καὶ) τὰ τοιαύτα’, (85r) ‘Γρ(άφεται) πεπονθότος’ – (145r) title added: **Πρὸς τὸν παρακοιμώμενον (τὸν) ᾿Απόκαυχον τῷ κ(αὶ) ὕστερον χρηματίσαντι μεγάλῳ δουκί**.

**Illustrations** (possibly scribal)**:** (54r) diagram of the urine vial divided into eleven areas, corresponding to John Aktouarios’ *On Urines* [Bk. I, ch. 13]: ‘διάγραμμα / νεφέλη / ἐναιώρημα / ὑπόστασις’ – (63r) X-shaped diagram showing the four qualities and labelled: ‘ὑγρότ(ερον) / ψυχρότ(ερον) / θερμότ(ερον) / ξηρότ(ερον)’ – (146r) X-shaped diagram showing the four qualities and labelled: ‘θερμ(όν) / ψυχρ(όν) / ὑγρ(όν) / ξηρ(όν)’ – (147v) four triangular diagrams showing the four qualities and labelled: ‘θερμότ(ερον) ὑγρότ(ερον) / ξηρότ(ερον)’ etc.

**Handwriting:** attributed by Brigitte Mondrain to Demetrios Angelos.

**Text added** by later hands**: [44r]** Partly erased recipe, inc. πιστάκια, des. εἰς πτύοντα. **[143v]** **Ζουλάπ(ιον) νοσοκόμ(ου) τοῦ Φωτ(ίου)**, inc. Λίθων θρηπτικ(ὸν) δεδωκιμασμέ(νον) ἀρτεμησί(αν)· βάτου ῥίζης· δάφνης ῥίζης· des. κοκυμήλ(ου). **[144r–v]** **᾿Επίθεμα καταρωικ(ὸν) τοῦ Ταρωνίτου**, inc. Προξυρίσας τὴν κε(φαλὴν) ἐπίθες κ(α)τ(ὰ) μέσον τῆς κορυφῆς, des. ἐρίκης στΓ α΄; **Εἰς πολυασθενεῖς κ(αὶ) πάνυ ἀδυνάτ(ους) ὠφελιμώτ(α)τ(ον) συντεθ(ὲν) παρὰ κ(υροῦ) ᾿Ιω(άννου) τοῦ ἀρ(ίστ)ου ἰατροῦ πν(ευμάτ)ων διαφορητικ(ὸν) καὶ εὕρεκτον**, inc. Ζιντζίβερι στΓ α΄, des. κυδωνί(ων).

**Annotations** (non-scribal)**:** (8r) ‘ζωτικ(όν) / ψυχικ(όν)’, (96v) ‘ση(μείωσαι) τοῦτο ὡς ἀναγκαῖον’ – (178r) title added: **Περὶ διαγνώσεως παθῶν.**

**Paper:** folder in 4°; chain distance 33 mm; watermark similar to Piccard 122469 (scissors), attested in 1455.

**Quire signatures:** (ff. 1–43) non-scribal, Greek numerals *Ie1* β (9r) through ζ (41r) – (ff. 44–211) scribal, Greek numerals *Ie1* (except for α΄ *Ii3* on f. 51v) α^ον^ (51v) through κα^(ον)^ (204r) – (ff. 212–332) possibly scribal, Greek numerals *Ie1* α (21r) through ιστ (326r).

**II.**

Eastern Mediterranean, ca. 1445 (from watermarks)

**Original order of leaves:** 333–379, 388–395, 380–387, 396–403.

**[333r–v]** **Πῶς γίνονται οἱ ἱδρῶτες [...]νι ἕπονται**, inc. ῞Επονται δὲ τοῖς πυρετοῖς κ(αὶ) ἱδρῶτες, τρόπω τοιῶδε, des. τελικὸν δὲ αἴτιον τῶν ἱδρώτων, ἡ ἐπομένη, εὐφορία κ(αὶ) δυσφορία· τῶν οὕτως εἰρημέν(ων) 
Sicurus, 23.31–24.35). **[333v–341r]** **Γαληνοῦ περὶ σφυγμῶν, ἐκ τοῦ βιβλίου αὐτοῦ**, inc. Αἴσθησιν ἐναρμονίῳ, τινί κινήσει· οἷον αἱμαντικὸν πληγῆ, διάθεσιν ὑπαγορεύον ἀοράτως, des. μέγας· μικρός· τραχύς· λεπτός· ταχύς· βραδύς· πυκνός· ἀραιός· καὶ ἀνώμαλος. **[341r–345v]** **Τοῦ αὐτοῦ Γαληνοῦ ἔτι περὶ σφυγμοῦ**, inc. ῾Ο μὲν σφυγμὸς, κίνησις εστι καρδίας καὶ ἀρτηρί(ας)· κατὰ διαστολ(ὴν) καὶ συστολήν, des. οἱ δὲ ἄλλοι, παρὰ φύσιν· ὅ τε ἀνώμαλος καὶ ὁ τεταγμένος 
Heiberg, I.81.16–88.18. **[345v–346r]** no title, inc. Πόσαι ποιότητες καθορῶνται ἐν τῇ διαστολῇ τῶν σφυγμῶν, des. ἀν(θρώπ)οις ἀνέλπιστος· ἐπεὶ πολλάκις καὶ παραυτίκα τελευτᾶ. **[346r–v]** **Περὶ τῆς ἐκ τῶν σφυγμῶν σημειώσεως.** [in margine] **᾿Αετίου**, inc. Προσδοκωμένης ἤδη τ(ῆς) κρίσε(ως), πρὸ πάντ(ων) ἅπτεσθαι, des. διὰ τῆς γαστρὸς τὴν κρίσιν δεῖ προσδοκᾶν ἔσεσθαι 
Olivieri, II.18.15–19.4. **[346v–354r]** **Περὶ τῆς ἐπὶ λουτροῦ τροπῆς**, inc. Λουτρὰ δὲ θερμὰ μέν, μεγ(ά)λ(ους) καὶ ταχεῖς κ(αὶ) πυκν(ού)ς, des. καὶ πλατύς· ἐνίοτέ ποτε κ(αὶ) τάσιν τῆς ἀρτηρί(ας) βραχεῖαν: τέλος. τέλος τοῦ περὶ σφυγμῶν Γαληνοῦ 
Kühn, VIII.468.11–492.4. **[354v–361v]** **Θεοφίλου περὶ διαχωρημάτων,** inc. ᾿Επειδὴ τὰς διαθέσεις τῶν ἐν βάθει κειμέν(ων) τοῦ σώματος μορί(ων), des. ἐπικαλούμενον Χ(ριστὸ)ν τ(ὸν) ἀληθιν(ὸν) Θ(εὸ)ν ἡμῶν εἰς τοὺς αἰώνας τῶν αἰώνων: ἀμήν 
Ideler, I.397–408. **[361v–363r]** no title, inc. ῾Ο ἐμπειρικώτατος πάντων (καὶ) οἰκεία σοφία τῶν ἀνθρωπίνων σωμ(ά)τ(ων) θεραπευτής, des. συνεχῶς ἐκχέη εἰς εἴκοσιν ἡμέρας ἀποθανεῖται 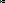
Sudhoff, 85–6, 106–8. **[364r–366v]** no title, inc. ᾿Αποφλεγματισμὸν τοῖς ὀδονταλγοῦσιν ἄριστον, σταφὶς ἀγρία διμασηθεῖσα, des. τῇ δήξει δὲ τὰ δριμέα βλάπτει 
Kühn, XIV.356.5–12, 355.11–356.4, 356.13–358.12, 359.2–364.2. **[366v–403v]** **Διαγνωστικὴ διάλεκτος τῶν μεγάλων ποιητ(ῶν) ἰατρῶν· περὶ τῶν ὀξέων νοσημάτ(ων), καὶ ὀξέων τὲ καὶ χρονίων: φρενίτιδος αἰτία**, inc. ᾿Ερασίστρατος μ(ὲν) ἐξ ἀκολουθῶν τῶν ἐαυτοῦ δογμάτων, des. καὶ εἰ μὲν παρηγοροῖντο ἐάσομ(εν)· εἰ δὲ παροξὺνοιντο 
Garofalo, 2–16.20, 22.9–136.6, 140.13–174.23.^35^

**Blank pages:** 404v.

**Text added** possibly by the original scribe**: [363v]** **᾿Επίθεμα ἡπατικ(ὸν) διὰ πείρας ἄριστον**, inc. ᾿Αμμωνιακ(όν) οὐγγ(ίαι) β΄, des. ἀμυγδαλέλαιον οὐγγ αs΄΄· κηρόν οὐγγ α΄. **῎Αλειμα**, inc. ᾿Αλόη· θείου ἀπύρου, des. ἐν ἡλίῳ καὶ τοῦτο εἰς τ(ὴν) ἔδραν θες. Inc. ῎Ασφαλτος ὅσον καρύου ποντ(ικοῦ) μέγεθος, des. ὠφελεῖ. Inc. Σημείωμα ὡς ὁ χυλός, des. πυρίκαυστα ὠφελεῖ. Inc. Καταρόνισμα ὑποστοιὸν εἰς παραφορὰν καὶ ἀϋπνι(αν), des. ἐλαίου ἕψησον χρῶ ὁμοί(ως). Inc. ᾿Αλειφή δραστήριος εἰς τὸ έκβάλαι σίδ(η)ρ(α)· ὀστᾶ· καὶ ἀκάνθας· ἐκ σαρκὸς, des. ἄλειμμα.

**Annotations** (scribal)**:** (334r, 334v, 335r, 337r, 339v, 348r, 348v, 351v, 354v, 355r, 356r, 357v, 357r, 357v, 358r, 359r, 363v, 365v, 367r, 368v, 370r, 373v, 377v, 378r, 380v, 381r, 384v, 385v, 387r, 392v, 396r, 396v, 397v, 401v) additions or corrections to the main text, variant readings, indications of contents, eg. (337r) ‘Γρ(άφεται) ῥυθμόν’ – (356v) list of the qualities/colours of urines: ‘λευκ(όν) / ξηρόν / λευκ(όν) / ὑγρ(όν)’.

**Handwriting:** similar to those of (according to Georgi Parpulov) Constantine Triboles (RGK II 318) and (according to Rudolf Stefec) Mark (RGK III 437).

**Recipes added** by later hands**: [333v]** Inc. ῾Η διὰ κρόκου σκευασία: χαλβάνης οὐγγ Γs΄΄, des. κρόκου οὐγγ α΄ στΓ δ΄. **[354r]** Εἰς τὴν κηδεί(αν)  ις: ιθ  α^ον^ εἰς τὸ μνημόσυνον  ια  (…) εἰς τὴν κηδεί(αν)  κβ: ιςs΄΄  α^ον^  εἰς τὸ μνημόσυνον  ΙΓ, inc. Εἰς ψόρ(αν) διὰ χυλοῦ· ῥοδελαίου· τετραφάρμακον, des. ἀγρυσταφίδ(ος). **[363r]** Inc. Μήλ(ων) αἱ σάρκης· μαστίχης, des. μέλιτος τὸ ἀρκοῦν. **[363r]** Erased text. **[364r]** Inc. Ζουλάπιον εἰς πτύων, des. γλυκάνισον. **[366v]** Inc. Εἰς τινὰ ἄν(θρωπ)ον ὅταν ἕξω ἔλθη τὸ κάθησμαν τοῦ, end lost.

**Annotations** (non-scribal)**:** (379v) Ζήτ(α) τ(οὺς) θεραπ(ευτικοὺς) ἔμπροσθ(εν) μετὰ τοῦτο τὸ τετράδ(ιον) τό ἑπόμ(ενον), inc. Ζήτ(α) τὸ σημείον τοῦτ(ο) ἐπειδ(ὴ) τετραδ(ίου) ἔσφ[αλται] – (333r) title added: **Περὶ ἱδρώτ(ων)** – (361v) title added: **Προγνωστικὰ τοῦ ῾Ιποκράτ(ους) διεξοδικῶς οὐ παρέδομ(εν) ἔτι ζῶν** – (380r) Rhaz(eus) lib(er) XIX. cap(itulus) XX.

**Illustration** (non-scribal)**:** (338v) partial x-shaped diagram of pulses.

**Paper:** folder in 4°; chain distance 38 mm; watermark very similar to Piccard 122 600 (anvil), attested in 1444.

**Quire signatures** (non-scribal)**:** Greek numerals *Im1* (except for α^ον^ *Im3* on f. 399v) β^ον^ (340r) through ζ (388r).

**A and B.**

**Quires:** 5  1 (v), 5  8 (f. 40), 3  1 (f. 43); 25  8 (f. 243), 10 (f. 253), 10  (f. 325), 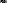 (f. 332; no text lost); 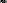 (f. 333), 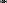 (f. 339), 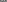 (f. 403), 1 (f. 404), 2 (f. 406), 1 (i).

**Binding:** *corio russico*. – Pastdowns and outermost flylaves: machine made-paper. – Flyleaves ii–iii in vol. 1 and 405–406: chain distance 25 mm, unclear watermark. – Flyleaves iv–v in vol. 1 and 404: chain distance 31 mm, watermark very similar to Piccard 150 772, 150 409, 150 636 (three hills), attested in 1461–77.

**Text on the flyleafs:** (IVv) two recipes: inc. ᾿Ιστέον ὅτι τὸ τούρπετε, καθάρει τὰ ἔντερα· τὰ κέπουλε τ(ὴν) κεφαλῆν (καὶ) τὸ φλέγμα· τὰ ξανθὰ, τ(ὴν) ξανθὴν χολ(ήν)· τὰ μέλανα, τ(ὸν) μελαγχολικ(ὸν) χυμ(όν), des. τὸ δακρύδιον, τ(ὴν) ξανθὴν χολήν. Inc. Τὰ δὲ τοῦ βοηθήμ(α)τος εἴδη εἰση ταῦτα· πικρὰ, des. ἀριστολοχία· πολυπόδιν – (V*r*) ἀκτουάριος – (V*r*) recipe: inc. Σκαμμωνίαν des. συκυόψηκτα ὀκαδ α΄ – (V*v*) ‘τοῦ σοφωτ(ά)του· καὶ λογιωτ(ά)του· καὶ ἄκρ(ου)· ἰατροῦ· πανσεβάστου· σεβαστοῦ κυροῦ ᾿Ιω(άνν)ου Ζαχαρίου τοῦ ἀκτουαρίου’.

**Marks of ownership:** (363r) ‘᾿Ι(ησο)ῦ Χ(ριστ)ὲ β[οή]θει τῷ σῷ δούλῳ [–]δικω τῶ ᾿Αρδυρομ^ττ^[?]’ [the following three lines of text are completely blotted out] – (404r) ‘Μανουῆλ Καντακουζινὸς ο Γεράκης’ – (flyleaf V*r*) ‘κ(αὶ) τόδε σὺν τῆς ἅλης μονῆς τοῦ Στ(αυ)ρονικήτα· τοῦ μεγάλου Νικολάου τ(ῆς) ἐν τῷ ῾Αγίῳ ῞Ορει’ – (flyleaf II*r*) ‘Ex Bibliotheca Askeviana / P. ii. Art 540 / J. Sims’.

**Provenance:** Manuel Kantakouzenos Gerakes (cf. Wellcome MS.MSL.114) – Stavronikita Monastery, Mount Athos – brought to England in 1749 – Anthony Askew (1722–74), London; [his sale, G. Leigh and J. Sotheby, London, 15 March 1785, lot 540]; purchased by James Sims (1741–1820), London, in 1785; purchased by the London Medical Society in 1802; purchased by the Wellcome Library in 1984.

Bibliography: *Bibl. Askev.*, 33 (no. 540); *Cat. Med. Soc*. 1803, 3; *Cat. Med. Soc*. 1829, 4; Daremberg, 158–9; Nias, liii–liv; Diels, I.41, 109, 131, II.7, 48, 102, 108–10, 131; Dawson, 59–60; Powell, *op. cit*. (note 34), 359–60; Gamillscheg, *op. cit*. (note 34), 287–300; Nutton, 24–6; Garofalo, *op. cit*. (note 35), xvii; Nutton and Zipser, 261; Touwaide, 538–9; Georgiou, *op. cit*. (note 34), 250–3, 364–7.
Georgios A. Costomiris, ‘Études sur les écrits inédits des anciens médecins grecs. Cinquième série, XII^e^–XIV^e^ siècles: Jean Tzetzès, Nicolas Myrepsus, Jean Actuarius’, *Revue des Études Grecques*, 10 (1897), 405–45: 441.
Donald Nicol, *The Byzantine Family of Kantakouzenos (Cantacuzenus) ca. 1100–1460: A Genealogical and Prosopographical Study* (Washington DC: Dumbarton Oaks, 1968) [*Dumbarton Oaks Studies*, 11], 128–129 with note 20.
Frank Stubbings, ‘Anthony Askew’s “Liber Amicorum”’, *Transactions of the Cambridge Bibliographical Society*, 6 (1976), 306–21: 317.
Brigitte Mondrain, ‘Jean Argyropoulos professeur à Constantinople et ses auditeurs médecins, d’Andronic Eparque à Démétrios Angelos’, in Georgios Makris and Cordula Scholz (eds), *Πολύπλευρος νοῦς:* *Miscellanea für Peter Schreiner zu seinem 60*. *Geburtstag* (Munich: Saur, 2000), 223–50: 236–7, 250.
*eadem*, ‘Comment était lu Galien à Byzance dans la première moitié du XVe siècle? Contribution à quelques aspects de l’histoire des textes’, in Antonio Garzya and Jacques Jouanna (eds), *Trasmissione e ecdotica dei testi medici greci: Atti del IV Convegno Internazionale, Parigi 17–19 maggio 2001* (Naples: D’Auria, 2003), 361–84: 366.
*eadem*, ‘Démétrios Angelos et la médecine: contribution nouvelle au dossier’, in Véronique Boudon-Millot *et al.* (eds), *Storia della tradizione e edizione dei medici greci: Atti del VI Colloquio internazionale, Paris 12–14 aprile 2008* (Naples: D’Auria, 2010), 293–322: 295, 299, 301, 305.
*Petros Bouras-Vallianatos*, ‘Medical Theory and Practice in Late Byzantium: the Case of John Zacharias Aktouarios (ca. 1275-ca. 1330)’ (unpublished PhD thesis: King’s College London, 2015), 125, 346–8, 382.

## 6. MS.MSL.60 (*olim* HH i 17 / We 30)

Eastern Mediterranean, ca. 1450–1500 AD (from watermarks).

Paper, 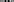, 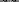 (foliated 1–221), linn. 34 [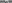], unclear ruling.

**[1r–11v]** [Hippocrates], *Aphorisms* (TLG 0627.012). **[12r–19r]** [Hippocrates], *Prognosticon* (TLG 0627.003). **[20r–45v]** Epitome of Nicholas Myrepsos’ *Dynameron*.^36^ **[46r]** Anonymous collection of questions and answers on the nature of man.^37^ **[46r–v]** Anonymus, *On the Creation of the World and Man* (TLG 0721.006). **[46v–48r]** Anonymus, *On Offspring* (TLG 0627.024).^38^ **[48r–50v]** Anonymus, Lexicon of medical synonyms.^39^ **[50v–53r]** Paul of Aegina, *Medical Epitome* (TLG 0715.001), excerpt on the substitution of drugs.^40^ **[53r–56r]** Anonymous, collection of opuscules on various functions of the human body. **[56r–57r]** Ps.-Galen, *On Weights and Measures* (TLG 0530.022), excerpt. **[57r–58r]** Anonymus, *On Stones*. **[58r]** Ps.-Avicenna, Short prognostic remedy. **[60r–62r]** Anonymous excerpts on astrology followed by Easter tables.^41^ **[62v]** Anonymous short excerpt on astrology. **[62v]** [Hippocrates], *On the Seven Divisions of a Man’s Life* (TLG 0627.044), excerpt. **[62v]** Recipe for a composite drug attributed to Saint Gregory the Theologian. **[62v]** Anonymous short excerpt on the four elements. **[63r]** Anonymus, recipe on dysuria. **[63r–71r]** Anonymous collection of astrological and geographical excerpts. **[71r]** Anonymous opuscule on human conception. **[71v]** Anonymous bilingual [Greek and Arabic] lexicon of plant names. **[73r–124v]** Anonymous medical compilation on diagnosis and therapy of diseases following, generally, an *a capite ad calcem* structure.^42^ **[125r–138r]** Collection of recipes attributed to Demetrios Pepagomenos. **[138r–v]** Anonymous opuscule *On Temperaments*. **[138v–142v]** Theophilos (Protospatharios) or Stephen, *On differences between the fevers* (TLG 0746.001), excerpt.^43^ **[142v–162v]** Symeon Seth, *On the Capacities of Foodstuffs* (TLG 3113.002). **[163r–169v]** Anonymous collection of opuscules on various medical matters.^44^ **[170r–171r]** Ps.-Galen, *On the Distinction of Urines*. **[171r–177r]** Theophilos (Protospatharios), *On Urines* (TLG 0729.002). **[177r–184r]** Anonymus, *On Urines*. **[184r–185v]** Anonymus, *On Excrements*. **[185v–187v]** Table of contents of John Zacharias Aktouarios’ *On Urines* (TLG 3188.002).^45^ **[187v–189r]** Nikephoros Blemmydes, *Canon on Urine Vials.* ^46^ **[189r]** Nikephoros Blemmydes, *Canon on Phlebotomy*. **[189v–191r]** Ps.-Hippocrates, *On Urines Vials*. **[191r]** Ps.-Galen, *On the Urine Vial*. **[191r–v]** *On Urines* attributed to the Persians (TLG 0721.013). **[191v]** Anonymous opuscule *On the Sanguine Complexion* (TLG 0721.003). **[192r–v]** Anonymous opuscule *On the Four Elements of the Human Body*. **[192v]** Anonymous opuscule *On the Five Senses*. **[193r–198r]** Ps.-Galen, *On Pulse*. **[198r–v]** Anonymous opuscule *On the Qualities of Pulse*. **[198v–199r]** Ps.-Galen, *Medical Definitions* (TLG 0530.041), excerpt on pulse. **[199r–205v]** Theophilos (Protospatharios), *On Pulse* (TLG 0729.004).^47^ **[206r–220r]** Ps.-Galen, *On Procurable Remedies* (TLG 0530.029), Book 1. **[220r–222v]** Anonymous collection of recipes for compound drugs.

**Text: [1r–11v]** **῾Ιπποκράτους ἀφορισμοί**: **βιβλίον α΄**, inc. ῾Ο βίος [βραχύς] ἡ δὲ τ[έ]χνη. [μακρ]ή· ὁ δ[ὲ καιρὸς ὀ]ξύς, des. ταῦτα χρὴ νομίζειν, ἀνίατα: τέλος τῶν ἀφορισμῶν ῾Ιπποκράτους 
Littré, IV.458–608; Jones, 98–216.15. **[12r–19r]** **Προγνωστικὸν ῾Ιπποκράτους**, inc. Τὸν ἰητρὸν δοκέει μοι ἄριστον εἶναι, πρόνοιαν ἐπιτηδεύειν, des. γνώση δέ, τοῖσιν αὐτέοισι σημείοισιν: τέλος τοῦ προγνωστικοῦ ῾Ιπποκράτους 
Littré, II.110–90; Alexanderson, 193–231; Jouanna, 1–80. **[20r–21v]** Table of Contents: **Πίναξ σὺν Θ(ε)ῷ τῶν σκευασιῶν τοῦ δυναμεροῦ**, inc. σκευασία το῀ν ναρδίνου μύρου α΄ / σκευασία τῆς θηριακῆς β΄, des. ἀντίδοτος διουρητικὴ Ταρωνίτου. **[21v–35r]** **Σκευασία τοῦ ναρδίνου μύρου**, inc. ᾿Ελαίου πρωτίου, des. ὀποβαλσάμου στΓ αs · μέλιτος τὸ ἀρκοῦν. **[35r–v]** no title, inc. Κρόκου· στΓ β΄, des. μέλιτος τὸ ἀρκοῦν. **[36r]** Table of Contents: **῞Ετερος πίναξ τῶν σκευασιῶν, τῶν ἐλαίων**, inc. Σκευασία τὸ γλεύκινον ἔλαιον α΄, des. σκευασία ἡ παναλοιφή ιε΄. **[36r–37v]** **Σκευασία τὸ γλεύκινον ἔλαιον**, inc. ᾿Ελαίου βενεφάρτου, des. ὄξους τὸ ἀρκοῦν. **[38r–39r]** Table of Contents: **Πίναξ τῶν σκευασιῶν τῶν ἐμπλάστρων**, inc. Σκευασία α΄ τοῦ πολυαρχίου, des. σκευασία πε΄ τῆς δι᾿ οἰνελαίου. **[39r–45v]** **Σκευασία τὸ πολυάρχιον,** des. ᾿Αμώμου οὐγγ΄ α΄, des. ἱκανῶς κ(αὶ) ἀνελόμενος χρῶ: τέλος. **[46r]** no title, inc. Τί ἔστιν ἄν(θρωπ)ος, ἄν(θρωπ)ος ἐστὶ ζῶον λογικὸν θνητόν, des. κ(αὶ) μειοῦται τὸ ζῶον. **[46r–v]** **Περὶ τῆς κατασκευῆς τοῦ κόσμου καὶ τοῦ ἀν(θρώπ)ου**, inc. ῾Ο κόσμος οὗτος ὁ μέγας συνέστηκ(εν) ἐκ τεσσάρων στοιχείων, des. ἕως ἐτῶν ὀγδοήκοντα καὶ ἕως γήρους 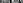. **[46v–48r]** **Περὶ γόνης,** inc. Νόμος μὲν παντων κρατύνη· ἡ δὲ γόνη τοῦ ἀνδρὸς ἄρχει πάντ(ων), des. ἔνδοθ(εν) σαρκώδης 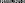. **[48r–50v]** **Λέξεις ᾿Ελλήνων, ἰατρῶν ἁπάντων κ(α)τ(ὰ) ἀ(λφά)β(ητον): ἀρχὴ τοῦ α΄,** inc. ῎Ακανθα Αἰγυπτία, ἀγριοκάρδαμος des. ὠταλγία, ὠτῶν πόνος: τέλος τῶν λέξεων 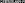. **[50v–53r]** **Περὶ ἀντεμβαλλομέν(ων): Παύλου Αἰγηνίτου ἰατροσοφιστοῦ κεφάλαιον εἰκοστὸν πέμπτον, ἐκ τοῦ ἑβδόμου βιβλίου· τὸ ἐπιγραφόμενον ἐκ τῶν Γαληνοῦ περὶ ἀντεμβαλλομέν(ων)**, inc. ᾿Εν ᾿Αλεξάνδρεια φησὶν ἐπὶ τινος γυναικὸς, des. ἀντὶ ὠοῦ τὸ λευκόν, γάλα γυναικίῳ: τέλος περὶ τῶν ἀντιμβαλλομέν(ων) Παύλου τοῦ Αἰγηνίτου 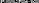. **[53r]** **Περὶ ἱδρώτων**, inc. Πόσα αἴτια ἱδρώτων, des. κ(αὶ) ὅταν εὐφόρως κενοῦντ(αι). **[53r–v]** **Περὶ τοῦ πόθεν πνεῖ τὸ καθ(ὲν) στοιχεῖον**, inc. Τὸ αἷμα διὰ ρινὸς πνεῖ, des. ἐμπεσόντα τῆς μήτρας. **[53v]** **Περὶ συκιάσεως**, inc. Τὸ συκίασμα ἐπὶ παιδί(ων) καὶ γερόντ(ων), des. εἰς δὲ τὴν χάσιν τῆς (σελήν)ης τοναντίον συμβαίνει. **[53v–54r]** **Περὶ πέψεως**, inc. Πέψις ἐστὶν ἐρήμασις τῆς ὕλης, des. καὶ ἀγανάκτησις τῆς δυνάμεως. **[54r]** **Περὶ ὀφελείας τῆς πέψεως τῆς νόσου**, inc. ῾Ο φόβος ἐστὶν τοῦ νοσήματος, des. θανάτους εἰς ἔνα τόπον γίνετ(αι). **[54r–v]** **Περὶ πέψεως ἀτελοὺς καὶ τελείας διάγνωσις**, inc. ῞Οτε ἐν τοῖς νοσήμασι, des. καὶ τὸ μόριον φθείρεται. **[54v–55r]** **Περὶ διαγνώσεως ἑκάστης πέψε(ως) ἀπὸ ποίου μέρους μέλλη γενέσθ(αι)**, inc. Χρὴ σκοπεῖν τὸν ἰατρόν, des. τὰ μόρια ξηρὰ εἰσίν. **[55r–v]** **Περὶ πέψεως**, inc. Τριττὴ ἐστὶν ἡ πέψις, des. μαγνίτης τὸν σίδηρον. **[55v–56r]** **Περὶ ζωτικῆς δυνάμεως**, inc. ῾Η ζωτικὴ δύναμις ἐκ τῆς ὑπάρξεως, des. τὰ σώματα δυσχερῶς δὲ ἀπὸ ψύξεως. **[56r–57r]** **Περὶ σταθμῶν Γαληνοῦ**, inc. ῾Ο σταθμὸς βάρει μετρούμενος κρίνετ(αι), des. ὅτι τῶν ξηρῶν οὐσιῶν ἄποιον ἐστὶ κ(α)τ(ὰ) τὴν διαφοράν: τέλος τῶν σταθμῶν 
Kühn, XIX.748.2–756.1. **[57r–58r]** **Περὶ τῶν ιβ΄ λίθων τῶν ἐν τῷ λογίῳ τοῦ ἱερέως λίθου σαρδίου τοῦ Βαβυλωνίου καλουμένου**, inc. Λίθος σάρδιος· ὁ τοιοῦτος, πυρωπός, des. εἰσιέναι τοῦ προσεῦξαι ὑπὲρ τοῦ λαοῦ, εἰς τὰ τῶν ἁγίων ἅγια 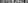. **[58r]** **Τοῦ ᾿Αβιτζιανοῦ προγνωστικὸν εἰς ἄρρωστον**, inc. ᾿Ιτέας φύλλ(α) κοπανίσας, des. εἰ δὲ χλωρόν, θνήσκει. **[60r]** **῾Ερμηνεία περὶ τοῦ θεμελίου τῆς (σελήν)ης**, inc. Εἰ θέλ(ης) εὐρεῖν τὸ θεμέλιον τῆς (σελήνη)ς, des. ἔστιν ὁ θέμελιος τῆς (σελήν)ης. **[60r]** **Περὶ τοῦ πόσον ἡμερ(ῶν) ἐστὶν ἡ (σελήν)η**, inc. Εἰ θέλ(ης) εὐρ(εῖν) καὶ πόσων ἡμερ(ῶν), des. κ(αὶ) τοῦ ἀπολειφθέντα ἔστιν ὁ ἀριθμὸς τῆς σελήν(ης). **[60r]** **Περὶ τοῦ νομικοῦ Φάσκα**, inc. Εἰ θέλ(ης) εὐρ(εῖν) καὶ το Πάσχα τὸ νομικόν, des. ἑπτὰ ὀφείλ(ει) προστιθέναι. **[60r]** **Πε(ρὶ) ἐ(ως) οὐ αναβένη κ(αὶ) κ(α)τ(ὰ)βένη τὸ Πάσχα κ(αὶ) τὸ Φάσκα**, inc. Χρὴ εἰδέναι ὅτι τὸ Πάσχ(α) τῶν χριστιανῶν ἀναβαί (νει), des. κ(αὶ) εὐρήσεις τὴν ἀπόκρεαν. **[60r–v]** **Πε(ρὶ) τ(ῆς) νηστεί(ας) τῶν ῾Αγίων ᾿Αποστόλων**, inc. Εἰ θέλ(ης) εὐρ(εῖν) τ(ὴν) τ(ῶν) ῾Αγ(ίων) ᾿Απο(στόλων) νηστεί(αν), des. ἡ νηστεία τῶν ῾Αγί(ων) ᾿Απο(στόλων). **[60v]** **Πε(ρὶ) τοῦ εὐρ(εῖν) ἀρχ(ὴν) ἢ ἑτ(έ)ρ(αν) ἡμέρ(αν) μι(νὸς) ἐν ποίᾳ ἡμέρ(ᾳ) τ(ῆς) ἐβδομάδ(ος) ἐστίν**, inc. Εἰ θέλ(ης) οὖν εὐρ(εῖν) ἡμέρ(αν) τοῦ μηνό(ς) des. ἕως ἂν ἔλθης, ἕως τοῦ Σα(ββά)τ(ου). **[60v]** **Περὶ τοῦ θεμελίου τῆς σελήν(ης)**, inc. Βάναι ἐπάνω εἰς τὸ θεμέλιον τὸ παλε αιόν, des. οὖτος ἐστὶν ὁ θεμέλιος τῆς σελήν(ης). **[60v]** **Πε(ρὶ) τοῦ κύκλου τ(ῆς) σελήν(ης)**, inc. ῞Οταν θέλης εὐρεῖν τὸν κύκλον τῆς σελήν(ης), des. οὗτο(ς) (ἔστιν) ὁ κύκλος τῆς σελήν(ης). **[60v]** **Πε(ρὶ) τοῦ πότε ἄρχοντ(αι)· ἥ τε (σελήν)η· ὁ ἥλιος κ(αὶ) ἡ (ἰνδικτιών)**, inc. Χρὴ εἰδέναι ὅτι ἡ σελήν(η)· ἄρχεται ἀπὸ τὸν ᾿Ιανουάριον, des. ἀπὸ τὸν Σεπτέμβριον. **[60v]** **῾Η σελήνη μ(ε)τ(ὰ) τῶν κύκλων καὶ θεμελίων**, inc. Α΄· ιδ΄, des. κα΄· ιθ΄· β΄. **[60v]** **Τα θεμέλια τοῦ (ἡλίου)· καὶ τῶν μην(ῶν)· πε(ρὶ) θεμελίων τοῦ (ἡλίου)**, inc. ῾Ο ἐνεστὼς κύκλος τοῦ ἡλίου· κ(αὶ) τὰ τούτου τέταρτα, des. ἔχουσι θεμέλιον· τρεῖς· οἱ δὲ λ΄· β΄. **[60v–61r]** **Πε(ρὶ) ἡλίου**, inc. ᾿Απὸ ἄκρη τῶν αἰώνων, des. ἔστιν ὁ κύκλο(ς) τοῦ ἡλίου. **[61r]** **Καὶ σελήν(ης)**, inc. ῾Ομοί(ως) ἀπὸ τὰ ἄκρη τῶν αἰώνων, des. ἔστιν ὁ κύκλος τ(ῆς) σελήν(ης). **[61r]** **Πε(ρὶ) ἕως οὐ ἀναβαίνη καὶ κ(α)τ(ὰ)βαίνει τὸ Πάσχ(α)**, inc. Χρὴ (δὲ) εἰδέναι ὅτι ἀπο τὴν κ΄ κ(αὶ) ἄνω ἡ ἀπόκρεω ἐστὶν ᾿Ιαννουαρίου, des. καὶ Πάσχ(α) ἐστὶν ᾿Απριλλίου. **[61r]** Paschal Table: inc. ᾿Ιστέον ὅτι ὁ μ(ὲν) πρώτ(ος) κύκλο(ς) δηλοῖ ἐν ποίᾳ ἡμέρ(ᾳ) ἔσται ἡ ἀπόκρεως, des. ὁ δὲ Δ^ος^ κύκλο(ς) τ(ὴν) τῶν ἁγί(ων) ἀποστόλ(ων) νειστεί(αν) ἀποδείνυσιν. A circular table entitled ‘Τοῦ Ξαναθόπουλου’ and glossed ‘ὀφείλεις μετρεῖν ἀπὸ κάτωθ(εν)’. **[61v]** Paschal Table with three columns and a hand accompanied by explanation; **῏Ηχος δ΄· ἔδοκας σημίοσιν**, inc. ῎Εχει πέντε Μάρτιος, des. τρεῖς καὶ τρεῖς Φευρουάρ(ιος). **[62r]** Paschal Table with ten columns entitled ‘Οἱ κύκλοι τοῦ ἡλίου. Πασχάλιον τετράγονον του Δαμασκηνοῦ’. **[62v]** Inc. Εἰς τὸ φέροντες, φέρε, φέρου, des. Δημοκρίτου. **[62v]** Inc. Εἰς μ(ὲν) γὰρ ἢν μαθεῖν, des. τὶ δὴ μαθ(εῖν). **[62v]** **Σέλα· ὄλα· νόλ· δέλα· ἰλά· φεκήθ· μαλά· ἄλ· μηλά· ἥλ· ἰλά· αὐλά** 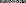. **[62v]** no title, inc. Χ(ριστ)ὲ Θ(ε)ὲ παντέλειε κτίστα κτίσεως πάσης, des. τὸν τριπαθέστατον ἐμὲ τάλαν 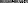. **[62v]** **῾Ηλικίαι εἰσὶ ἑπτά· ὡς φησὶν ῾Ιπποκράτ(ης) ἐπτὰ εἰσὶν ὥραι ἃς ἡλικί(ας) καλέομ(εν)**, inc. Παιδίον· παῖς· μειράκιον, des. ὁ δοῦλος κἂν ἢ γέρων 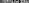. **[62v]** **᾿Αλάτιον σκευασθὲν παρὰ τοῦ ἐν ἁγίοις Γρηγορίου τοῦ Θεολόγου· ποιηθ(ὲν) διὰ στίχ(ων) παρὰ Ρωμανοῦ χαρτοφύλακος** **καὶ πρωτασηκρήτη**, inc. ῝Ως θεῖα ῥητὰ Γρηγόριος καὶ ξένα· λέξας πρὸς ἡμᾶς, des. ἔσθιε βρώμασιν οἷσπερ ἂν θέλης 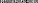. **[62v]** **Πρὸ(ς) τὸ ὅλην ἀποθέμενοι**, inc. Πῦρ θερμὸν ξηρὸν ἐστίν· ἀὴρ θερμὸς καὶ ὑγρός, des. τὸ δὲ φθεινόπορον, τῆ γῆ κρᾶσις ἄριστος. **[63r]** **Πρὸς δυσουρίαν**, inc. Σκευασία ἐνεργοῦσα εἰς δυσουρί(αν), des. μετὰ ζωμοῦ τερεβίνθου μαῦρου δραχμῆς α΄· καὶ ἰσχάδας πέντε. **[63r–v]** **᾿Αρχὴ σὺν Θ(ε)ῶ τῆς περὶ οὐ(ρα)νοῦ καὶ γῆς ἑρμηνείας**, inc. Τὸ τῆς γῆς σχῆμα οὔτε τρίγονον ἐστὶν οὔτε τετράγωνον, des. ἑρμηνεύετ(αι) δὲ ἡ γῆ γένους μέσων τῶν τεσσάρων στοιχεί(ων). **[63v]** **᾿Ερώτ(ησις)**, inc. Ποῦ ἴσταται ἡ γῆ, des. ἡ γῆ βαστάζεται. **[63v]** **Τῶν σοφῶν γνῶμαι**, inc. ῎Αλλοι πάλιν μέλουσι λέγειν ἡμᾶς, des. ὅτι ἐνδέχετ(αι) νὰ ἰσομετροῦνται συνίστανται ὁ κόσμος ὠς μέλλ(ων) νὰ ἐξισάζονται. **[63v–64r]** **Περὶ Δ^ων΄^ στοιχείων**, inc. Τέσσαρα στοιχεῖα κ(α)τ(ὰ) τὸ μέτρον ὅπως ἱσάζονται, des. τινὸς οἴκον μὴ δυνάμεθα ἐγγίζων ποθ(ὲν) καὶ οὕτως νόει. **[64r]** **Περὶ διαστήματος γῆς**, inc ῎Ενι δὲ τὸ διάστημα τ(ῆς) γῆς, des. τὸ πλάτος τοῦτο ὡς οἱ ἀκριβ(ῶς) γεωμέτραι ἀπεφήναντο. **[64r]** **Περὶ μιλίων καὶ σταδίων**, inc. Τὸ στάδιον ἔνι ὀργίαις ρ΄, des. ἡ πιθαμὴ ἔχει δακτύλους ιβ΄. **[64r]** **Περὶ τῶν ἀντιπόδων τῶν ὑπὸ κάτωθ(εν) τῆς γῆς**, inc. Καὶ ἀντιπόδων λέγουσ(ιν) ἀστροσκοπούντ(ων) παῖδες ὅτι εἰσὶν ὑπὸ κάτω τ(ῆς) γῆς ἄν(θρωπ)οι, des. καὶ οὕτ(ως) ἀντιστομοῦντ(αι) οἱ συλαλοῦντ(ες) 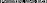. **[64r–v]** **Περὶ τοῦ ποταμοῦ**, inc. ῎Εστι ποταμὸς λεγόμενος ὠκεανός, des. διὰ τὸ πλησιάζοντα τὴν μακαρί(αν) γῆν τοῦ παραδείσου 
Delatte 1929–30, 515.13–22. **[64v]** **Περὶ παραδείσου**, inc. ῾Ο παράδεσος οὐκ ἔσω εἰς τὴν καθ᾿ ἡμᾶς γῆν, des. ἐκ ψυχῆς νοερὰς καὶ σώματο(ς) φαινομ(έν)ου, οὕτως νόει τὸν παράδεισον 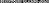. **[64v]** **Περὶ παντοί(ων) φυτῶν**, inc. Φυτῶν μ(ὲν) παντοί(ων) τὰ εἴδη κέκτηται, des. καὶ οὕτ(ως) ἐπιγίνωσκε τ(ὸν) παράδεισον. **[64v–65r]** **Περὶ Τῶν τεσσάρ(ων) θαλασσῶν τ(ῆς) γῆς**, inc. Θάλασσαι (δὲ) εἰσὶ τέσσαρ(αι) ἐπὶ τῆς ὅλης γῆς, des. τὸ μαργαριτάριν τὸ ὑπέρτιμον καὶ ὁ λίθος ὁ ἐρυθρό(ς) 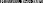. **[65r]** **Περὶ δευτὲρας θαλάσσης**, inc. Δευτέρα θάλασσα ἡ ἀρχὴ ἀπὸ ᾿Αλεξανδρί(ας), des. γίνετ(αι) ὁ λίθος ὁ πράσινος. **[65r]** **Περὶ τῆς τρίτ(ης) θαλάσσης**, inc. ῾Η δὲ τρίτη θάλασσα ἄρχετ(αι) ἀπὸ Βυζαντίου καὶ γυρίζει τὴν Καλαβρί(αν), des. ἐστὶν ἐκ τῶν Γαδήρων τὴν ἀλμυρότητα δεχόμ(εθα). **[65r]** no title, inc. ῾Η τετάρτη θάλασσα ἡ μεγάλη καὶ μαύρη, des. λίμναι κληθήσονται καὶ οὐ θάλασσαι. **[65r–v]** **Περὶ τῶν θερμῶν ὑδάτων**, inc. Περὶ τῶν θερμῶν ὑδάτων φασί τιν(ες) ὅτι ἐκ στενοπῶν φλεβῶν ἐξέρχομαι, des. θερμὰ μᾶλλον καὶ ψυχρά 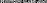. **[65v]** **Περὶ σεισμοῦ**, inc. ῾Ο σεισμὸς οὗτος γίνετ(αι) ἐγκαστρωθεῖσα ἡ γῆ παρὰ τῶν ἀνέμων, des. οὐ πᾶσαν τὴν γῆν ἀλλὰ διὸ πλησιάζει καὶ μὴ ἀπιστήσης 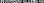. **[65v]** **Περὶ ἀνέμων**, inc. ᾿Ιδοὺ γὰρ ἡμεῖς πολλάκις ἀνέμων πληρωθεὶς τῶν ἡμετέρων φλεβῶν, des. ὅτι ὁ ἐπιβλεψάμενος ἐπὶ στ(αυ)ροῦ προ(ς) τὴν γῆν διέσεισεν 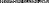. **[65v–66r]** **Περὶ νεφελῶν**, inc. ᾿Αναγκαίον (δὲ) ἐστὶ περὶ νεφελῶν· διαλαβεῖν καὶ λέγομεν, des. κ(αὶ) οὕτως γίνετ(αι) ἡ χάλαζα. **[66r]** **Περὶ χείονος**, inc. ῾Η δὲ χείονος γίνετ(αι) οὕτως· καὶ παχυνθείσης τ(ῆς) νεφέλ(ης), des. πῶς ἑκάστου γίνετ(αι) πλήρωσις. **[66r–v]** **Περὶ νεφελῶν πληθυνόντων**, inc. Τῶν νεφελῶν ἐπάνω πληθυνθ(έν)των κ(αὶ) τῶν μ(ὲν) ἔνθα, des. κ(αὶ) ἀυτὸς λέγ(ε)τ(αι) κεραυνός. **[66v]** **Περὶ ἀστραπῆς**, inc. ῾Η ἀστραπὴ π(ῶς) γίνετ(αι) ὑποδείξω ἡμῖν, des. ια΄ λιβόνοτος· ιβ΄ θρασκί(ας). **[66v]** **Περὶ τῶν τ(ῶν) πιπτόντ(ων) ἐκ τοῦ οὐ(ρα)νοῦ κ(αὶ) λέγουσιν τινὲς ὅτι εἰσὶν ἀστέρες**, inc. Οἱ κομ(ή)ται οὐκ εἰσὶν ἀστέρες ὥς τινες λέγουσ(ιν), des. καὶ τούτῳ ψευδὲς οὐ γὰρ ἐν τῷ οὐ(ρα)νῷ παρόντων αἰών(ων) πν(εύματ)α πονηρά 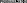. **[66v–67v]** **Περὶ τοῦ οὐ(ρα)νίου σώμ(α)τος**, inc. ῾Ο οὐ(ρα)νὸς λέγει ὁ μέγας Βασίλειος ὅτι ἐστὶ κονοειδὲς ἄλλοι (δὲ) λέγουσιν ὅτι ἐστὶ κρυσταλοειδὲς, des. φανερὰν ποιούμ(εν) ὑμῖν πότε εἰσέρχετ(αι) ὁ ἥλιος ἐν αὐτοῖς. **[67v–68r]** **Περὶ ἐμβολ(ῆς) καὶ ἐκβολ(ῆς) κ(αὶ) ἡσοχῆς τοῦ ἡλίου ἐν τ(οῖς) ζωδί(οις) π(ῶς) εἰσέρχ(ε)τ(αι)**, inc. ᾿Επειδὴ τὰ ιβ΄ ζώδια ὥσπερ χορ(ὸν) κυκληκ(ὸν) κρατοῦσ(ιν), des. γινώσκετ(αι) τὸν τοῦ ἡλίου δρόμον ἐν τοῖς ζωδίοις. **[68r]** **Περὶ ἐμβολ(ῆς) κ(αὶ) ἐκβολῆς τῆς σελήνης ἐν τοῖς ζωδίοις**, inc. ᾿Ιδοὺ διεχαράξωμ(εν) κ(αὶ) ἐφανερώσαμ(εν) τὸν τοῦ ἡλίου δρόμον, des. κ(α)τ᾿ ἄλλον τρόπον καθεκάστην χαράτομεν αὐτήν. **[68r–v]** **Περὶ τὴν ποσότητα τ(ῆς) σελήν(ης)**, inc. Γίνωσκε ὅτι τὴν ποσότητα τ(ῆς) σελήνης ἐκ τῶν θεμελί(ων) αὐτῶν εὐρίσκομεν, des. ἅπαντας τοὺς μῆνας εὐρίσης πόσων ἡμερῶν ἔνι ἡ σελήνη. **[68v–69r]** **Περὶ τὸ πῶς λαμβάνει ἡ σελήν(η) ἐκ τοῦ ἡλίου τὸ φ(ῶς) κ(αὶ) αὔξετ(αι) κ(αὶ) λήγει**, inc. ῾Ο μ(ὲν) ἥλιος ἐν τῷ τετάρτῳ οὐ(ρα)νῷ ἐστίν, des. καὶ στοχάζων αὐτὸν κ(αὶ) βλέπ(ων) αὐτόν. **[69r]** **Περὶ τοῦ π(ῶς) πιάνετ(αι) κ(αὶ) οὐ φωτ(ίζεται)**, inc. ᾿Επειδὴ ὡς προείπομ(εν) ἐκ τοῦ ἡλίου λαμβάνει ἡ (σελήνη) τὸ φῶς, des. ἐκτὸς οὖν τῶν αὐτῶν οὐ πιάνεται ἡ (σελήνη). **[69r–v]** **Περὶ τοῦ π(ῶς) πιάνετ(αι) ἐν τῷ οὐ(ρα)νῷ ὁ ἥλιος**, inc. ῾Ο ἥλιος ἐστὶν ἐν τῷ Δ^ῳ΄^ οὐ(ρα)νῷ ἡ (δὲ) (σελήνη) ἐν τῷ φαινομ(έν)ῳ παρ᾿ ἡμῶν, des. τῆς σελήν(ης) ἦν δὲ θαῦμα καὶ οὐ κ(α)τ(α) φύσιν τάξεως. **[69v]** **Περὶ ἀγαθῶν ζωδίων καὶ πονηρῶν καὶ μέσων**, inc. Κριός· Δίδυμος· Παρθένος, des. πλὴν δὲ τὰ τέλη τῶν πραγμάτ(ων) εἰς καλὸν ἀποβαίνουσιν. **[69v]** **Πε(ρὶ) τῶν Δ^(ων)^**΄** τροπ(ῶν) τοῦ ἡλίου κ(αὶ) π(ῶς) ποιεῖ τὰς τεσσάρας ὥρας τοῦ χρό(ν)ου· τὸ ἔαρ· τὸ θέρος· τὸ μετώπορ(ον) κ(αὶ) ὁ χειμ(ῶν)**, inc. ῎Εστι (δὲ) καμάραι τρεῖς καὶ ἡ μ(ὲν) μία καμάρα ἐστὶ πρὸ(ς) τὸ βόρειον μέρος, des. καὶ γίνεται ἐν ταῖς τρισὶν ταύταις ἡμέραις τέσσαρες. **[69v]** **Περὶ τοῦ πῶς εὐρίσκει ἑνὸς ἑκάστου ἀν(θρώπ)ου ζώδιον**, inc. Τούτου (δὲ) λάμβανε τὸ ὄνομα τοῦ ἀνδρὸ(ς) τὰ στοιχεία, des. καὶ εὐρίσης περὶ αὐτῶν τὸ εὔτυχον κ(αὶ) ἄτυχον 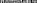. **[70r]** **Πε(ρὶ) τοῦ πότε στρατεύει κατεχρῶν**, inc. Τῆς σελήν(ης) οὔσ(ης) ἐν τῷ Λέοντι, des. κ(αὶ) οὕτως ἐστὶν τῶν ζωδίων βασιλεί(αν) 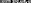. **[70r]** no title, inc. Δεῖ γινώσκ(ειν) ὅτι τὰ ζώδια μόνα καὶ ὁ ἥλιος μόνος οὐ ποιοῦσι κοσμικὴν δίαιτα, des. τοῦ ἡλίου μικροτ(έ)ρ(ου) τῆς σελήν(ης) κ(αὶ) διὰ τοῦτο ταχύτ(ε)ρ(ον) γυρίζει αὐτόν. **[70r–v]** **Περὶ οἴκων τ(ῶν) πλανητ(ῶν)**, inc. Εἰσὶ τινὰ ζώδια ἅπερ εἰσὶ κ(α)τ(ὰ) ποιότ(η)τ(α) κ(αὶ) κράσει, des. πέφυκ(εν) εἶναι κ(αὶ) οὕτως ἐστίν. **[70v]** **Περὶ τοῦ διαστήματος τοῦ οὐ(ρα)νοῦ κ(αὶ) τ(ῆς) γ(ῆς)**, inc. Οἱ μ(ὲν) τῶν ῾Ελλήν(ων) διδάσκαλοι τριακοσί(ας) χιλιάδας μίλια, des. παρὰ γὰρ τοῦ τὰ πάντα κ(α)τ(ὰ)σκευαστοῦ 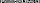. **[70v]** **Περὶ τοῦ θεϊκοῦ θρόνου**, inc. ᾿Επάνω δὲ τοῦ εὐδόμμου οὐ(ρα)νοῦ ἐστὶν ὁ τοῦ Θ(εο)ῦ θρόνος, des. κ(αὶ) οὐ χυλικὰ ποῦ γὰρ χώρεσις ὑλικῶν ἐν τοῖς ἀΰλοις 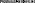. **[70v–71r]** Inc. Πόσα τάγματα εἰσὶ τῶν ἀγγέλων· πρῶτον ἀρχαὶ β^ον^ ἐξουσίαι, des. τὰ θελήμ(α)τα τοῖς ἀν(θρώπ)οις φανεροῦνται 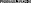. **[71r]** **Πε(ρὶ) τὸ πῶς οἱ ᾿Ιουδαῖοι καλοῦσι τὴν γῆν**, inc. Δεῖ γινώσκ(ειν) ὅτι κ(α)τ(ὰ) ᾿Ιουδαί(ους) ἡ γῆ ἀδάμαν λέγετ(αι), des. καθῶς φησὶν ἡ θεία γραφή 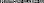. **[71r]** **Περὶ τῆς συλλήψεως τοῦ ἀν(θρώπ)ου κ(αὶ) τῆς κ(α)τ(ὰ)σκευῆς τῆς γυναικὸς κ(αὶ)** **γεννήσ(εως)· καὶ θανάτου καὶ τῆς προσκυνήσεως ἐν τῷ τοῦ Θ(εο)ῦ θρόνῳ**, inc. Συνουσιαζομ(έν)ου τοῦ ἀνδρὸ(ς) μ(ε)τ(ὰ) τῆς γυναικὸς αὐτοῦ, des. διὰ τοῦτο καὶ ἐν τῆ τρίτ(η) κ(αὶ) θ΄ κ(αὶ) μ΄ ποιούμ(εν) μνημόσυνον τοῦ τεθνηκότος 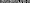. **[71v]** **Συριακὰ ὀνόματα βοτανῶν**, inc. ᾿Ασαφέτιδα: ἀλτήλ, des. λημναίαν σφραγίς: ταμούχημ: τέλος (in four columns) 
Serikoff, 102–4. **[73r–77v]** Table of Contents: **Πίναξ ἐκλογῶν τινῶν**, inc. Εἰς πρίσμα κοιλί(ας) ὅτ(αν) γένηται σκληρή, des. φξς΄ τὸ διὰ κυδωνίων Βλεμίδους. **[78r–124v]** no title, inc. Εἰς πρίσμα κοιλίας ὅταν γενηται σκληρή: Λινόκουκον, des. ὅτε ἄδιψος ἔχεις ὠσὶ καρύου ποντικοῦ τὸ μέγεθος: τέλος. **[125r–126v]** Table of Contents: **Πίναξ ἐκλογῶν τινῶν συνέθετο κεφαλαιοδῶς ὁ μακαριώ(τα)τ(ος) ἐκεῖνος σοφώτ(α)τ(ος) λογιώτατος ἀνὴρ ὁ Πεπαγωμένος· καὶ ἐν ἰατροῖς ἀριστότ(α)τ(ος),** **inc.** **Κουκ(ία) ὀφέλιμα εὐστόμαχα καὶ καθαρτικά,** **etc, inc.** **Βισησὰ σπέρμα ἐστὶ τοῦ ἀγρίου πηγάνου,** **etc.** inc. α΄ πρὸ(ς) τὸ γεννῆσαι ταχέ(ως) γυναῖκα, des. ρσε΄ κουκ(ία) εἰς αἱμορροίδας 
Capone Ciollaro, 43–48.196. **[126v–138r]** no title, inc. α΄ Πρὸς τὸ γεννήσαι ταχέ(ως) γυναῖκα, des. μετὰ ὕδατος σκεῦος καὶ πέσειται ὁ σκώληξ 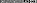. **[138r–v]** **Περὶ τῶν κράσεων**, inc. Τῶν κράσεων τὸ ποσον ὅτι ἐγγέα, des. καὶ τὰ μ(ὲν) ὑγιεινὰ τὰ δὲ νοσερὰ· τὰ δὲ οὐδέτερα. **[138v–142v]** **Στεφάνου φιλοσόφου· περὶ διαφορᾶς πυρετῶν**, inc. Σκοπὸν ἔχομεν ἐν τῷ παρόντι συγγράμματι περὶ διαφορᾶς πυρετῶν, des. ξηροτέρου τοῦ σώματος· οἷα τῶν ταρηχευομ(έν)ων σωμάτων: τέλος, τοῦ περὶ τέχν(ης) Στεφάνου 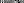. **[142v–162v]** **Τοῦ φιλοσώφου Συμεὼν τοῦ Σῆθ**, inc. ᾿Αρνῶν κρέα σύμμετρα τῇ θερμότ(η)τ(ᾳ), des. ὠτίδων σὰρξ μ(ε)τ(α)ξὺ χηνῶν καὶ γεράνων: 
Langkavel, 20.7–22.24, 23.9–29.28, 30.11–33.20, 34.1–60.26, 61.8–67.19, 71.14–6, 68.8–71.13, 73.12–80.23, 84.3–85.22, 87.5–88.12, 85.22–86.20, 83.15–84.2, 81.12–83.14, 88.13–99.14, 100.5–12, 99.15–100.4, 100.13–103.7, 104.12–106.8, 107.11–109.10, 111.7–23, 109.11–111.6, 112.1–123.15, 18.4–20.6, 123.16–125.13. **[163r–164r]** **Περὶ τοῦ δεῖ ποτίζειν τὰ βοηθήματα**, inc. ᾿Εὰν ἡ ξανθὴ χολὴ περιττεῦει, des. ὡσαύτως κ(αὶ) ῥοδέλαιον θερὸν ἄληφε τὸ ὑπογάστριον. **[164r–v]** **Περὶ ὀροῦ καὶ τῆς ἐνεργείας αὐτοῦ**, inc. ῾Ρίπτει ὑπαγει γαστέρα, des. καὶ πάλιν καθαίρουσι μ(ὲν) οὖν ἰσχυρῶς. **[164v–165v]** **Περὶ τοῦ τίνας διδόναι φάρμακον**, inc. Παιδία· γέροντας τοὺς φύσει ξηρούς, des. τὴν τε χολὴν κ(αὶ) τὸ φλέγμα. **[165v–166r]** **Περὶ τοῦ τίνας δεῖ ἐμεῖν**, inc. Τὸν ἔχοντα στεν(ὸν) κ(αὶ) βαθῆ στῆθος, des. καὶ ληφθὲν ἔμετον κινεῖ. **[166r–169r]** **Διάγνωσις περὶ φλεβοτομί(ας)**, inc. ῾Η φλεβοτομία καθολικὴ ἐστὶν κένωσις, des. τὸ αἷμα τῶν ἀρτηριῶν (δὲ) λεπτότερον κ(αὶ) ξανθότερ(ον). **[169r]** **Περὶ φλεβοτομίας ἐκ ποίων τόπων φλεβοτομεῖ χρῆ**, inc. Τέμνην χρὴ φλέβας κ΄, des. κ(αὶ) κ(α)τ(ὰ) τ(ὴν) τοῦ τόπου ευκρασί(ας). **[169v]** **Περὶ τοῦ τὶ δύναται ἡ φλεβοτομία**, inc. Δύναται τὰ πεπληρωμένα, des κ(αὶ) περιόδους ἀνακαλέσασθαι. **[170r–171r]** **Περὶ οὖρων Γαληνοῦ διαίρεσις**, inc. Οὖρον λευκὸν μὴ ἔχον ὑπόστασιν, des. τὸ χλωρὸν οὖρον, δηλοῖ θερμασί(αν) πλείστην· καὶ κακοήθειαν τοῦ σώματος 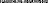. **[171r–177r]** **Περὶ οὔρων Μάγνου ἀπὸ φωνῆς Θεοφίλου**, inc. Τὴν περὶ τ(ῆς) τῶν οὔρων διαφορᾶς πραγματεί(αν), πολλοὶ τῶν ἀρχαί(ων) ἰατρῶν, des. ἐπιθυμούντ(ων) ἐκ πάσης προαιρέσεως: τέλος 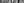. **[177r–184r]** **Περὶ τῶν πέψεων τοῦ οὔρου**, inc. Πέψεις εἰσὶ Γ΄· πρῶτη ἐν στομάχῳ, des. εἴδου βενετίζουσα τέλειον εἶναι σημεί(ον). **[184r–185v]** **Περὶ διαγνώσεως διαχωρημάτ(ων) κεφάλαια ζ΄: περὶ κόπρου πολῆς καὶ ὀλίγης**, inc. ῾Η πολὴ κόπρος αἴτια β΄ ἔχει, des. αἱ δὲ παχεῖαι ὀλίγον αἷμα γενῶσι κόπρον (δὲ) πολ(ήν). **[185v–186r]** **Τοῦ σοφωτ(ά)τ(ου) καὶ λογιωτ(ά)τ(ου) ὀκταρίου κυροῦ ᾿Ιωάννου, πραγματεία περὶ οὔρων: τάδε ἐνεστιν ἐν τῷ περὶ διαφορᾶς οὔρων λόγῳ**, inc. ῞Οτι χρήσιμος ἐκ τῶν οὔρων ἐπίσκεψις, des. περὶ διαγνώσεως φαιῶν πελιδνῶν καὶ μελάνων οὔρων 
Leone, xv–xvi; Georgiou, 398–400; Leone 59.8–60; Mathys, I.161–162.4. **[186r]** no title, inc. Τοῦ διαστήματος τοῦ χύματος, des. παρυφιστάμενα λέγεται 
Ideler, II.19.21–34. **[186r–187v]** **Τάδε ἔνεστιν ἐν τῷ β**^**ῳ΄**^ **περὶ διαγνώσεως οὔρων λόγῳ**, inc. Περὶ τῆς ἐκ τῶν κ(α)τ(ὰ) τὰ οὖρα συστάσεων διαγνώσεως, des. ἐπίλογος· ἐν ᾧ καί τις ἀξίωσις πρὸς τοὺς ἐντυγχάνοντας τῇ πραγματείᾳ ταύτης: τέλος 
Leone, 105–6, 161–2, 229.8–230, 298–9, 354; Mathys, 193–194.16, 232.8–233.11, 280.5–281.18, 329.4–330.9, 367.20–368. **[187v–189r]** **Διάγνωσις τοῦ σοφωτ(ά)τ(ου) καὶ λογιωτ(ά)τ(ου) κυροῦ Βλεμίδους δια στιχερ(ῶν) καὶ κανῶνος ἰατρικ(ὸν) περιέχοντα ὑέλια τῶν ἀρρωστούντ(ων) κ(αὶ) ὅσαι τούτ(ων) θεραπείαι κ(αὶ) οἷαι πεφύκασιν: ἦχος α΄ τῶν οὐ(ρα)νίων**, inc. Τῶν ἀσθενῶν ὑέλια μάθε τρὶς καὶ δέκα, des. τὸ βορβορῶδες καὶ ὄζον σημεῖον ἴσθι θάνατον 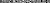. **[189r]** **Εἰς τοὺς αἴνους στιχ(η)ρ(ά) ἦχος β**^**ος**^ **:** **῞Οτε ἐκ τοῦ ξύλου σε νεκρόν**, inc. Μάθε καὶ τὰ αἷματα λοιπὸν τ(ῆς) φλεβοτομίας, des. μιμνήσκου τούτων καὶ ἐμοῦ τοῦ ὑπομνήσαντος: τέλος τοῦ κανῶνος 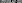. **[189v–191r]** **῾Ερμηνεία τῶν ὑελίων ἐν συνόψει καθ᾿ ῾Ιπποκράτην**, inc. Τὸ πρῶτον ὑέλιον ἐστὶν ἄσπρον· τὸ δεύτερ(ον) ξανθόν, des. καὶ σπλὴν οὐδ᾿ ὅλως εὑρέθη, ὅτι ἄλλο οὐκ ἔβοσκον, εἰ μὴ ἁλμυροριχί(αν). **[191r]** **῾Ερμηνεία τοῦ Γαληνοῦ περὶ κλοκείου**, inc. ᾿Επάρε τὸ κλοκίον, καὶ θὲς αύτὸ εἰς ἀσφάλιαν διὰ μιᾶς ὥρας τῆς νυκτός, des. ἔνι γὰρ τὸ ἧπαρ αὐτοῦ βεβλαμμ(έν)ον. **[191r–v]** **Σύνοψις ἀκριβεστάτη περὶ οὔρων, ἐρμηνευθεῖσα ἐκ τῶν ἰατρικῶν τεχν(ῶν) τῶν Περσῶν**, inc. Δεῖ γιγνώσκειν ὅτι ὅταν ἔστι τὸ οὖρον, des. εἰ δ᾿ οὐ βενετίζει, τέλειον εἶναι ση(μ)εῖ(ον) 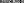. **[191v]** **Περὶ χροίας αἵματος**, inc. Τὸ κατάμαυρον αἷμα, ὅτι ἰχῶρα οὐ ποιήσει, des. φοινικοῦν ἔχει χρῶμα, μακρονοσί(αν) δηλοῖ 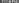. **[192r–v]** **Περὶ τῶν** **δ΄^**(ων)**^** **στοιχεί(ων) τοῦ σώματος**, inc. ᾿Ιστέον ὅτι τὰ τέσσαρα στοιχεῖα τοῦ σώματος, des. φλέγμασι διὰ τὴν τῶν σιτίων δριμύτητα. **[192v]** **Περὶ τῶν πέντε αἰσθήσεων**, inc. Πέντε μὲν εἰσὶν αἱ αἰσθήσεις, des. ἔχει περὶ αὐτὴν τ(ὴν) μνήμην. **[193r–198r]** **Γαληνοῦ περὶ σφυγμῶν**, inc. ῾Ο σφυγμὸς κίνησις (δὲ) ἀρτηριῶν ἀπὸ καρδί(ας) ἀρχομένης, des. εἰς τὸ βάθος τοῦ σώματος· ὕστερον δε. **[198r–v]** **Περὶ σφυγμῶν**, inc. Πόσαι ποιότ(η)τ(ες) θεωροῦνται ἐν τῇ διαστολῇ τῶν σφυγμ(ῶν), des. ση(μ)εί(α) κ(α)τ(ὰ) ψύξιν. **[198v–199r]** **Γαληνοῦ**, inc. ῾Ο σφυγμὸς (ἐστὶ) διαστολὴ καὶ συστολὴ καρδίας, des. τοῦτο ἡ συστολὴ ἐστίν; 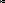
Kühn, XIX.375.16–376.13, 402.18–403.8, 365.16–366.6, 376.15–378.2. **[199r–205v]** **Θεοφίλου περὶ σφυγμῶν**, inc. ῞Οτι μ(ὲν) β΄ κοιλίαι τῆς καρδί(ας) εἰσίν, des. τὰ δὲ ἄλλα φυλάττουσι κ(α)τ(ὰ) φύσιν 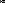
Ermerins, 3–7.5, 11.14–21.11, 23.21–2, 21.12–23.20, 33.14–73.7, 61.17–8, 57.21–3. **[206r–220r]** **Γαληνοῦ εὐπορίστων**, inc. Τὴν ἰατρικ(ὴν) τέχν(ην), des. θαυμάσ(εις) δὲ πάνυ δεξάμ(εν)ο(ς): τ(ῶν) εὐπορίστ(ων) ἰαμ(ά)τ(ων) δὴ πέρας 
Kühn, XIV.311.3–389. **[220r–222v]** **᾿Αντίδοτο(ς) πρὸ(ς) ποδαλγικούς· πάνυ πεπειρεμένη: αἰγυπτιακὴ τρισμέγιστο(ς)**, inc. Φοβερὰ δόκιμος γεναμένη εἰς ὑγεί(αν) πολλ(ῶν) ἀν(θρώπ)ων, end lost.^48^

**Annotations** (scribal)**:** (1r) ‘᾿Ι(ησο)ῦ παρ[άσχου]’ – (72r) ‘Χ(ριστ)ὲ προηγοῦ τῶν ἐμ(ῶν) πονημάτ(ων)’ – (1v, 7r, 11v, 15r, 36v, 50v, 68r, 70r, 81r, 110v, 131r, 156v, 170v, 173v, 174r, 178r, 181r, 181v, 182v, 183v, 193v, 194v, 209v, 219r) additions to the main text, variant readings, indications of contents, eg. (1v) ‘Γρ(άφεται) ἔτεος’, (11v) ‘ἐνέστι ταῦτα’.

**Illustrations** (scribal)**:** (2v) diagram with numbers α through κ corresponding to the Hippocratic *Aphorism* 24 (ch. 2) – (61r) paschal table with concentric circles – (61v) lunar table with two double columns in the sides and a palm with letters inscribed in the phalanxes – (62r) paschal table in 10 columns – (63r) small diagram representing the earth as circular and labelled ‘ἡ γῆ τὸ σχήμα αὐτῆς στρογγυλὸ ειδὸς κρέμμαται’ – (186r) diagram of a urine vial corresponding to the main text and labelled ‘ἀμίς’, ‘νεφέλη / ἐναιώρημα / ὑπόστασις’, ‘ὅλον τοῦτο λέγετ(αι) χῦμα’.

**Handwriting:** Georgi Parpulov identified the scribe with John (RGK I 204).

**Texts added** by later hands**:** (19v) inc. Τῶρα πλανήται ταῖς ἐμπανταῖς, des. κ(αὶ) οὐτος ποίι πάντα κ(αὶ) ποτ(ε) να μην σφάλη – (34r) beside a recipe: ταῦτη σκευασίαν εἶναι δια τον στόμαχον καλο – (58v) inc. ῎Ενας ἄν(θρωπ)ος, ποχι ζωτικὸν τίποτες, des. ἀπὸ πανου κ(αὶ) ἀπο κάτ(ου). Inc. ῞Ετερον κολοκινθί(ας) ρύζα, des. κίμηνον. Inc. Περη νανε βασι κρί(ας), des. βαζης απανου – (59r) inc. Εἰς σκο[-]ην ὁπου ἔχη πόν(ων), des. να την πείνει. Inc. Εἰς δυσεντερίαν, des. δυο χουληαριους. Inc. Οταν πό(νον) του ἀν(θρώπ)ου, des. του απο το βηζυν. Inc. Εις το να εγνωρύσας, des. αλήθιαν. Inc. Εἰς γωνατα, des. βράδυ κ(αὶ) τὸ μεσημέρι – (59v) inc. Μηνὶ δ(ε)υτέρω ὁ ἐστὶν Μαρτίω, des. πληάδες εσπέριοι κρύπτονται 
Wachsmuth, 289.10–6; Olivieri, 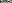
–5 ^49^ – (35r–v) inc. Κρόκου στΓ β΄, des. μέλιτος τὸ ἀρκοῦν – (72r) excerpt from a recipe for theriac: **Σύνοψις ἐν επιτόμῳ περὶ τ(ῶν) βοηθημάτ(ων)· καὶ τοῦ τρόπου τ(ῆς) δόσεως αὐτ(ῶν) ἰδί(ων) προπομ(ά)τ(ων)· ὁμοί(ως) (καὶ) περὶ ἐλιγμάτ(ων)· (καὶ) τροχίσκων· πρὸ(ς) τούτοις (δὲ) (καὶ) περὶ ἐλαί(ων)· (καὶ) ἐμπλάστρων· (καὶ) λοιπῶν τῶν εἰς θεραπείαν νοσημάτων διαφόρ(ων) συντεινόντ(ων)**, inc. ῾Ο περὶ τ(ῶν) ἀντιδότων· (καὶ) ἐμπλάστρων κ(αὶ) ἐλαίων λόγος (...) ἀντίδοτος ἡ θηριακή, des. μ(ε)τ(ὰ) συμφύτου ῥίζης – (72v) Ηδωρ με γενα και φοσφωρος με τρεφι βασιλ(εις) κ(αι) αρχ(ον)ταις με αγαπουτο αδικο την μιτερα μου γευγομε του θανατου. Μην Μαγιος εις τ(ὴν) λ΄.

**Annotations** (non-scribal)**:** (1r, 2v, 4r, 19r, 19v, 20r, 34r, 37v, 45r, 58v, 59r, 59v, 60v, 61v, 62r, 62v, 63r, 66r, 67v, 68r, 69v, 70r, 72r, 72v, 80r, 80v, 82r, 82v, 83r, 83v, 84v, 85r, 85v, 86r, 88r, 89r, 89v, 90r, 90v, 91r, 91v, 92r, 94v, 95r, 99v, 100r, 101r, 104v, 105r, 106r, 107r, 108r, 109r, 93v, 113v, 114v, 115r, 117v, 118r, 121v, 122r, 122v, 123r, 123v, 125r, 126v, 127r, 129r, 129v, 130r, 132r, 133r, 137r, 137v, 138r, 142v, 143r, 144v, 145r, 145v, 146r, 146v, 147v, 149r, 149v, 150r, 152r, 153v, 155v, 156r, 162r, 163r, 165r, 168v, 169r, 169v, 198v, 205v, 209v, 210r, 211v, 212r, 212v, 213r, 213v, 214r, 214v, 215v, 216r, 216v, 217r, 217v, 218v, 221v), eg. (2r) ‘εγενε επασε ημνον την ταφην σου των μονο βασιλεα’, (19r) ‘τέλος τοῦ προγνωστικοῦ: ῾Ιπποκράτους το κιτρωβάλσαμον· λέγετε το μελεις ωφείλ(ων)’, (79r) ‘τὸ οξιφί(νι)κον τὸ λεγη καὶ τουρκικά μηρχέντι’, (129v) long comment on the effectivenes on a certain recipe of the text, inc. Τὸ εμπλάστρι ὀπου γένεται εἰς τὸν πόνων τῶν ποδαρίων, des. κ(αὶ) θετις το εἰς τὸν πόνον, (149r) ‘τα κρομίδια να τα βραξης να τα τρογεις ἢνε καλα’, (171r) ‘Rhazis lib(er) xx’ – (78r) title added: **Τοῦ σοφωτάτου καὶ λογιωτάτου Γαληνοῦ (καὶ) ῾Ιπποκράτους· Παύλ(ου) (καὶ) ᾿Αετίου καὶ ἑτέρ(ων) πλήστ(ων) ἰατρ(ῶν) παλαιῶν**.

**Drawings** by later hands**:** (1r, 1v, 73v, 106v, 160v) obscure figures – (19v) drawing of two hands accompanied by a Paschal calculation, inc. τῶ ἀπω κτίσεως κόσμου ἔτ(ει) ζη[?]α καὶ απω τ(ῆς) τοῦ Ι(ησο)υ Χ(ριστο)υ οἰκονομί(ας) 178[?], des. καὶ ἔγινεν μεγάλει ἀπηλή – (59r) concentric circles.

**Paper:** folded in 4°; chain distance 33 mm; watermark very similar to Piccard 123360 (arrows), attested in 1455 AD.

**Quire signatures:** (scribal) Greek numerals *Im3* α΄ (27v), *Im1* β΄ (28r) and γ΄ (36r), *Ie1* γ΄ (70r, 73r) through στ^ον^ (97r), *Ie3* δ΄ (88v), *Ii3* ε΄ (96v), *Ie1* α΄ (206r).

**Quires:** 8 (f. 8), 2 (f. 10), 8 (f. 18), 1 (f. 19), 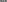 (f. 43), 2 (f. 45), 6 (f. 51), 8 (f. 59), 12 (f. 71), 1 (f. 72), 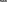 (f. 112), 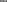 (f. 120), 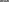 (f. 201), 4 (f. 205), 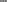 (f. 221).

**Binding:** *corio russico*, same tooling as Wellcome MS.MSL.1. – Flyleaves: chain distance 25 mm, no watermarks.

**Marks of ownership:** (72v) ‘᾿Απο ἐμένα τον Θεοδορω των Καλόγερακη εις σεσενα Κηρηζη Λασκαρακη’ – (72v) Records of loans: ‘Θέλη με κ(αὶ) ἠ Μάρθα ασπρ(ες) νε΄. Θέλη με ὁ θείος μου ὁ Μακρόπουλος ασπρ(ες) ρ΄.’ Etc. – (217v) unidentified stamp – (72v) monokondylion: ‘Ελευθεριος Δουκαινος ῥήτωρ’ – (205v) monokondylion: ‘῎Ιμβρου ᾿Ιωακεῖμ’^50^ – (205v) ‘Τὸ παρ(ὸν) ίατροσόφιον ὑπάρχη κτῆμα τῆς ἱερᾶς μον(ῆς) τοῦ ἁγίου Διονυσίου κ(αὶ) ἀγωράστη παρ᾿ ἐμοῦ Εὐθυμίου ἱερομονάχου κ(αὶ) προηγουμένου τῆς αὐτῆς μον(ῆς) τὸ ͵αχκη΄ ἡ ἐν τῇ Καλιούπολῃ τῆς Θράκης’ – (37v) ‘Το παρὸν ιατροσόφιον υπάρχη Συμεὸν ἱερομόναχου καὶ προηγουμένου του ἁγίου Διονισίου μονῆς τοῦ τημίου Προδρόμου’ – (59r) monokondylion: ‘Μητροφάνης ᾿Ιερωμόναχος (καὶ) πρωτοσύγγελος’^51^ – (front flyleaf V) ‘H Hi 17’.

**Provenance:** bought in Gallipoli by Euthymius, former abbot of the Dionysiou Monastery, Mount Athos, in 1628 – Symeon, former abbot of the Dionysiou Monastery, Mount Athos – Anthony Askew (1722–74), London; [his sale, G. Leigh and J. Sotheby, London, 15 March 1785, lot 577]; purchased by James Sims (1741–1820), London, in 1785; purchased by the London Medical Society in 1802; purchased by the Wellcome Library in 1984.

Bibliography: *Bibl. Askev.*, 35 (no. 577); *Cat. Med. Soc*. 1803, 96; *Cat. Med. Soc*. 1829, 155, 286; Daremberg, 159–162; Nias, liii–liv; Diels, I.5, 13, 48, 100, 115, 125, 128, 131, 133, II.7, 79, 80, 98, 101–102, 109; Dawson, 68–72; Weinstock, *op. cit*. (note 48), 33–38 (no. 56); Alexanderson, *op. cit*. (note 48), 76; Tartaglia, *op. cit*. (note 39), 548; Capone Ciollaro, *op. cit*. (note 48), 12–20; Nutton and Zipser, 261; Touwaide, 538–9; Georgiou, *op. cit*. (note 34), 253; Jouanna, *op. cit*. (note 48), lxxxv, xci; Serikoff, *op. cit*. (note 48), 97–121.
Alain Touwaide, ‘Lexica medico-botanica byzantina: Prolégomènes à une étude’, in Lois Pérez Castro *et al.* (eds), *Τῆς φιλίης τάδε δῶρα*: *Miscelánea léxica en memoria de Conchita Serrano* (Madrid: Consejo Superior de Investigaciones Científicas, 1999), 211–228: 215, 227.
*idem*, ‘On uroscopy in Byzantium’, in Thanasis A. Diamandopoulos (ed.), *Ιστορία της Ελληνικής Νεφρολογίας* (Athens: Παπαζήσης, 2000), 218–20: 219.
*idem*, ‘Arabic urology in Byzantium’, *Journal of Nephrology*, 17 (2004), 583–9: 585.
Maria Capone Ciollaro, ‘Per l’edizione delle *Eclogai* di Demetrio Pepagomeno’, in Antonio Garzya and Jacques Jouanna (eds), *Trasmissione e ecdotica dei testi medici greci: Atti del IV Convegno Internazionale, Parigi 17–19 maggio 2001* (Naples: D’Auria, 2003), 40–52: 40.

## 7. MS.MSL.62 (*olim* HH i 23 / We 32a)

Italy, ca. 1520–40 AD (from watermarks).

Paper, 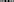, 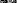 (foliated 1–55, 56–88, 89–155)

**[1r–31r]** Aretaeus of Cappadocia, *On the Causes and Symptoms of Acute Diseases* (TLG 0719.001). **[31v–78v]** *Idem*, *On the Causes and Symptoms of Chronic Diseases* (TLG 0719.002). **[78v–122v]** *Idem*, *On the Cure of Acute Diseases* (TLG 0719.003).**[127r–139v]** Rufus of Ephesus, *On the Nomenclature of the Human Body* (TLG 0564.003). **[139v–143r]** Ps.-Rufus of Ephesus, *On the Parts of the Human Body* (TLG 0721.001). **[143r–150r]** Rufus of Ephesus, *On the Parts of the Human Body* (TLG 0564.004).**[150r–152v]** *Idem*, *On Bones* (TLG 0564.005).

**Note:** This MS consists of two distinct parts, described below separately as **A** and **B**.

**A.**

Linn. 20 [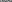], 20D1.

**Text: [1r–2v, 5r–7v]** **᾿Αρεταίου Καππαδόκου ὀξέων νούσων, βιβλίον α΄**^**ον**^ **:** **Περὶ διαβήτου** **:** **Γ΄**^**ον**^, inc. ῞ϒδρωπος ἰδέη τὸ διαβήτεω, des. ἐπὶ τῶνδε φοίνιξις· περίπατοι· ῥαθϋ 
Hude, 162.1–167.2. **[3r–4v]** no title, inc. ῟Ησσον πάσχουσι· μᾶλλον δὲ θνήσκουσιν, des. τῆς ἀναπνοῆς τὴν τροπὴν οὐκ  *id.*, 6.4–8.18. **[8r–v]** **Περὶ τετάνου**, inc. Σπασμοὶ οἱ τέτανοι, des. νέοι δὲ τουτέων  *id.*, 5.14–6.4. **[9r–13v]** no title, inc. ᾿Αδύνατ(ον) γίγνεσθαι ἔνδοθ(εν), des. εὐτυχίη, ἐν τῷδε τῷ πάθει  *id.*, 8.18–14.6. **[14r]** Table of Contents: **᾿Αρεταίου Καππαδόκου ὀξέων παθῶν αἴτια καὶ σημεῖα· βιβλί(ον) β΄** **:** **Τάδε ἔνεστιν ἐν τῷ δευτέρῳ βιβλίῳ**, inc. Περὶ περιπνευμονίης: α΄, des. Περὶ σατυριάσε(ως): ιβ΄ 
Ermerins, 22. **[14r–31r]** **Περὶ περιπνευμονίης**, inc. Δυοῖσι τοῖσι, des. οὐκ ἴσχει ὑστέρην ἀνήρ 
Hude, 15.1–35.12. **[31v]** Table of Contents: **᾿Αρεταίου Καππαδόκου περὶ χρονί(ων) παθῶν αἰτίων καὶ σημείων βιβλί(ον) α΄**^**ον**^ **:** **τάδε ἔνεστιν ἐν τῷδε τῷ βιβλίῳ**, inc. Προοίμιον: α΄, des. περὶ καχεξί(ας): ιζ΄  Ermerins, 60. **[32r–54r]** **Προοίμι(ον)**, inc. Χρονίων νούσων, πόνος μ(ὲν), des. κορυφ(ὴν) τελεσφορεῖ· θέρος δὲ κτείνει 
Hude, 36.1–61.23. **[54v]** Table of Contents: **᾿Αρεταίου Καππαδόκου χρονίων παθῶν σημειωτικὸν, βιβλίον β΄**^**ον**^ **: τάδε ἔνεστιν ἐν τῷδε τῷ βιβλίῳ**, inc. Περὶ ὕδρωπος: α΄, des. περὶ ἐλεφαντιάσε(ως): ΙΓ΄ 
Ermerins, 108.**[54v–78v]** **Περὶ ὕδρωπος**, inc. ῞ϒδρωψ, ἀτερπὲς μ(ὲν) εἰσιδεῖν πάθος, des. ἐς τέρατος ἰδέην 
Hude, 62.1–90.22. **[78v–79r]** Table of Contents: **᾿Αρεταίου** **Καππαδόκου θεραπευτικὸν ὀξέ(ων) νοσημάτων: βιβλί(ον) α΄: τάδε ἔνεστιν ἐν** **τῷδε τῷ βιβλίῳ**, inc. Προοίμιον: α΄, des. θεραπεία πλευρίτιδος: Ι΄ 
Ermerins, 158. **[79r–102v]** **Προοίμι(ον)**, inc. ᾿Οξέων νούσων ἄκεα ξυναψέα, des. ἢ ἐς ἐμπύησιν τρέπεται 
Hude, 91.1–118.24. **[102v–103r]** Table of Contents: **᾿Αρεταίου** **Καππαδόκου ὀξέων παθῶν θεραπευτικὸν βιβλί(ον) δεύτερ(ον): τάδε ἔνεστιν ἐν** **τῷδε τῷ βιβλίῳ**, inc. Θεραπεία περιπνευμονίης: α΄, des. θεραπεία σατυριάσε(ως): Ια΄ 
Ermerins, 200. **[103r–122r]** **Θεραπεία περιπνευμονίης**, inc. Κάτοξυ καὶ ἐπίκαιρ(ον) κακὸν φλεγμασίη, des. κ(αὶ) μούνοισιν ἢ ἅπασι χρέεσθ(αι): τέλος 
Hude, 119.1–143.12. **[122r]** Scribal Colophon: Γήθει μ(ὲν) λιμένα πλωτὴρ πολυβενθέα μάρπτων, / γήθει δ᾿ αὖ γραφεύς, στίχ(ον) ὕστατ(ον) ἐκτολυπεύων 
cf. Vassis, 118.^52^

**Blank pages:** 122v–126v.

**Annotations** (scribal)**:** (61r, 66r, 73r, 79v, 83r, 83v, 84v, 87r, 88r, 88v, 90r, 91r, 99v, 101v, 107r, 108r, 109r, 109v, 110r, 110v, 111r, 111v, 115r, 115v, 117r, 118r, 120r, 121v) additions to the text, variant readings or short explanations in the form of synonyms, eg. (61r) ἴσ(ως) ὀσχέου, (84v) ἤγου(ν) ποτίζειν, (91r) ὁ αὐχ(ὴν) τοῦ στομάχου.

**Handwriting:** unidentified Humanist hand.

**Paper:** folded in 4°; chain distance 30 mm; watermark similar to Piccard 122768 (ladder), attested in 1528.

**Catchwords** (scribal)**:** vertical, *Ii3*.

**Quire signatures** (scribal)**:** Greek numerals *Ie1* α΄ (1r) through ιστ΄ (119r).

**B.**

Linn. 22 [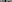], unclear ruling.

**Text: [127r–139v]** **῾Ρούφου ᾿Εφεσίου ὀνομασίας τῶν τοῦ ἀνθρώπου μορίων**, inc. Τι πρῶτον ἔμαθες ἐν κιθαριστικῇ, des. διά τινα ὀλίγα παροφθέντα 
Daremberg, 133–67; Kowalski, 20–106. **[139v–143r]** **Τοῦ αὐτοῦ ὀνομασίῶν τῶν κατ(ὰ) ἄνθρωπον α΄**^**ον**^, inc. Εἴ τις τὴν κιθαριστικὴν μέλλοι, des. δάκτυλοι δὲ ὁμοί(ως) τοῖς τῆς χειρὸς ὀνομάζονται 
Daremberg, 233–36. **[143r–150r]** **Τοῦ αὐτοῦ περὶ ὀνομασιῶν β΄**^**ον**^, inc. Παραδόντες τὴν τῶν ἔξωθεν, des. διαπαντὸς ὑπὸ ὀστέων περιεχόμενος  *id.*, 168–85. **[150r–152v]** **Τοῦ αὐτοῦ περὶ ὀστέων γ΄**, inc. ᾿Επειδὴ τὴν ἐντόσθιον θεωρίαν, des. κ(αὶ) ἡ τῶν ὀστέων θέσις: τέλος  *id.*, 186–94.^53^

**Blank pages:** 153r–155v.

**Annotations** (scribal)**:** (128r, 128v, 129r, 129v, 131r, 133r, 133v, 135r, 138v, 139v, 150r) additions to the text or variant readings, eg. (133r) ‘χειρῶν’, (150r) ‘Γρ(άφετ)αι κατ(ά)’.

**Handwriting:** unidentified Humanist hand.

**Paper:** folded in 4°; chain distance 32 mm; watermark very similar to Piccard 122766 (ladder), attested in 1533.

**Catchwords** (scribal)**:** vertical, *Ii3*.

**Quire signatures:** (probably scribal) partly lost in trimming: Greek numbers *Ie1,* eg. δ΄ (150r).

**A. and B.**

**Quires:** 1 (i), 2 (ii–iii), 1 (iv), 8 (ff. 3 and 4 misplaced; original order 1, 2, 5, 6, 7, 8, 3, 4), 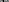 (f. 126), 7 (f. 133, no text lost), 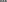 (f. 149), 6 (f. 155), 1 (i).

**Binding:** *corio russico*. – Flyleaves: (i and i) machine-made paper; (ii–iii, front pastedown) chain distance 24 mm, no watermark – (iv) chain distance 40 mm, watermark generally similar to Briquet 5252 (croissant), attested in 1530–2 – (back pastedown) chain distance 30 mm, no watermark.

**Annotations** (non-scribal)**:** (back pastedown) ‘βληστρίζεσθαι ὀλισθαίνειν lib. p° 15 cap. / deest [?] cap. 2’.

**Marks of ownership:** (front flyleaf II) ‘Ex Bibliotheca Askeviana / P. ii. Art. 551. / J. Sims’ – (1r) ‘A. Askew M. D.’ – (1r) ‘3.28’.

**Provenance:** Anthony Askew (1722–74), London; [his sale, G. Leigh and J. Sotheby, London, 15 March 1785, lot 551]; purchased by James Sims (1741–1820), London, in 1785; purchased by the London Medical Society in 1802; purchased by the Wellcome Library in 1984.

Bibliography: *Bibl. Askev.,* 34 (no. 551); *Cat. Med. Soc*. 1803, 10; *Cat. Med. Soc*. 1829, 13; Daremberg, 162–3; Nias, liv; Diels, II.17–18, 89; Dawson, 74; Nutton, 23–5; Nutton and Zipser, 261; Touwaide, 538–9.
Francis Adams (ed.), *The Extant Works of Aretaeus, the Cappadocian* (London: Sydenham Society, 1856), xvii.
Carmelo Cutolo, ‘Sulla tradizione manoscritta di Areteo di Cappadocia’, *Galenos*, 6 (2012), 25–47: 26.

## 8. MS.MSL.109 (*olim* MM c 7 / Wf 7)

Italy, ca. 1510 AD (from watermarks).

Paper, 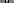, 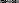 (paginated 1 [f. 1v]–163, then foliated 82–318), linn. 30 [ca. 235 x 125], unclear ruling.

Aetios of Amida, *Tetrabiblon*, Books 9^54^ (TLG 0718.009), 10^55^, 11^56^ (TLG 0718.011), 12^57^ (TLG 0718.012), 13^58^ (TLG 0718.013), 14^59^, 15^60^ (TLG 0718.013).

**Text: [1r–v]** Table of Contents: **Τάδε ἔνεστιν ἐν τῷ ἐνάτῳ λόγῳ**, inc. Περὶ τῶν κατὰ τὸ στόμα τῆς κοιλί(ας) παθῶν, des. Περὶ λειεντερί(ας) 
Zervos 1911, 272. **[1v–52v]** **᾿Αετίου ᾿Αμηδινοῦ λόγος ἔνατος. περὶ καρδιακῶν,** inc. ῞Οπως μὲν χρὴ θεραπεύειν τοὺς καρδιακ(οὺς) λεγομένους, des. ἀνάπλασσε μεθ᾿ ὕδατος ὀροβιαῖα μεγέθη κ(αὶ) δίδου ε΄ ἢ ζ΄ πρὸς δύναμιν: τέλος τοῦ ἐνάτου λόγου  *id.,* 273–390. **[52v–53r]** Table of Contents: **Τάδε ἔνεστιν ἐν τῷ ι΄ λόγῳ**, inc. Περὶ ἀτονί(ας) ἥπατος κ(αὶ) ἐφ᾿ ὧν αἷμα διὰ γαστρὸς φέρεται, des. κοινὴ δίαιτα πάντων τῶν ὑδρωπικῶν ᾿Αρχιγένους  
Da Monte, II.198; Cornarius, 619–20. **[53r–81v]** **᾿Αετίου ᾿Αμιδηνοῦ λόγος δέκατος. Περὶ ἀτονί(ας) ἥπατος κ(αὶ) ἐφ᾿ ὧν αἷμα διὰ γαστρὸς φέρεται**, inc. Δυσκρασίαι μὲν αἰτίας τῆς τοῦ ἥπατος ἀτονί(ας) εἰσί, des. εὔκρατος (δὲ) πρὸς τ(ὴν) ὤραν τοῦ ἔτους κ(αὶ) ὁ οἶνος: τέλος τοῦ θ΄ λόγου  
Da Monte, II.199–248; Cornarius, 619–64.**[163–82]** Table of Contents: **Τάδε ἔνεστιν ἐν τῷ ια΄ λόγῳ**, inc. Περὶ διαβήτου, des. περὶ τῶν ἐπαφροδισίοις χρῆσθαι μὴ δυναμένων  
Da Monte, II.249–250.12; Cornarius, 663–4. **[82r–110v]** **᾿Αετίου ᾿Αμηδινοῦ βιβλίον ἑνδέκατον. περὶ διαβήτου**, inc. ῾Ο διαβήτης χρόνιον ἐστι πάθος, des. κ(αὶ) διαίτῃ πάσῃ κεχρῆσθαι θερμῇ κ(αὶ) ξηρᾷ  
Daremberg, 85.1–96.17, 569.7–576.8, 96.18–107.3, 576.9–577.8, 108.1–109.8, 577.9–40, 109.9–117.5, 578.2–580.18, 117.6–119.22, 107.4–26, 119.23–126.6, 580.19–581.37, 126.7–24. **[110v–112r]** Table of Contents: **Πίναξ τοῦ ιβ΄ βιβλίου ᾿Αετίου**, inc. Περὶ ἰσχιάδος, des. ξηρίον ἐπιπασσόμενον τῷ ποδαγρικῷ φιλώθρῷ 
Costomiris, 1–6.4. **[112r–149r]** **᾿Αετίου ᾿Αμηδινοῦ βιβλίον ιβ΄. περὶ ἰσχιάδος**, inc. ᾿Ισχιαδικοὶ κυρίως καλοῦνται, des. ἐγὼ ἴσα βαλὼν κ(αὶ) κισσήρεως μᾶλλον ὠφέλησα   *id.,* 7–129.18. **[149r–151r]** Table of Contents: **Πίναξ τοῦ ΙΓ΄ λόγου ᾿Αετίου**, inc. Περὶ ἀνθρωποδήκτων, des. περὶ λέπρας  
Da Monte, II.356–8; Cornarius, 757–8. **[151r–208v]** **᾿Αετίου ᾿Αμηδινοῦ βιβλίον ΙΓ**^**ον**^ **. περὶ τῶν δακνόντων ζώων κ(αὶ) τ(ῶν) ἰοβόλων θηρίων κ(αὶ) περὶ τ(ῶν) πολυχρήστων ἀντιδότων κ(αὶ) περὶ ἐλεφαντιάσε(ως) κ(αὶ) κνησμοῦ κ(αὶ) ψωριάσε(ως). περὶ ἀνθρωποδήκτων**, inc. ᾿Αρχόμενοι περὶ τῶν δακνόντων ζώων γράφειν, des. κ(αὶ) ἐὰν αποβρέχεσθαι τεσσαράκοντα ἡμέρας ἐν κῶ[?]  
Zervos 1906, 264–6; 
Da Monte, II.362.18–22; Cornarius, 762.31–7; 
Zervos 1906,; 
Da Monte, II.362.32–364.14; Cornarius, 762.51–764.7; 
Zervos 1906, 267.14–270.18; 
Da Monte, II.366.18–23; Cornarius, 766.1–8; 
Zervos 1906, 270.19–286.3; 
Da Monte, II.374–376; Cornarius, 772.61–775.42; 
Zervos 1906, 286.4–14; 
Da Monte, II.377.11–25; Cornarius, 775.56–776.12; 
Zervos 1906, 286.15–289.10; 
Da Monte, II.379.3–384.19; Cornarius, 777.33–782.7; 
Zervos 1906, 289.11–291.12; 
Da Monte, II.385.1–19; Cornarius, 782.51–783.13; 
Zervos 1906, 291.13–292; 
Da Monte, II.386–438; Cornarius, 784.45–832.8. **[208v–210r]** Table of Contents: **Πίναξ τοῦ ιδ** ^**ου**^ **λόγου τοῦ ᾿Αετίου**, inc. Περὶ τῶν ἐν ἕδρᾳ παθῶν, des. περὶ τῶν ἐν σκελέσιν ἢ ἄλλῳ τόπῳ τοῦ σώματος κιρσῶν  
Da Monte, III.3–4; Cornarius, 831–2. **[210r–276r]** **᾿Αετίου ᾿Αμιδηνοῦ βιβλίον ιδ**^**ον**^ **. περὶ τῶν ἐν ἕδρᾳ παθῶν**, inc. Τὰ κατὰ τὴν ἕδραν πάθη, des. χρὴ (δὲ) ἐπὶ ἡμέρας τεσσαράκοντα ἡλιάζεσθαι κ(αὶ) τρίβεσθαι  
Da Monte, III.5–69.30; Cornarius, 833–904. **[276r–277r]** Table of Contents: **Πίναξ τοῦ ιε** ^**ου**^ **λόγου ᾿Αετίου**, inc. Περὶ οἰδημάτ(ων), des. ἡ διὰ πομφόλυγος ἥρα καππάδοκος 
Zervos 1909, 139–141. **[277r–316r]** **᾿Αετίου ᾿Αμηδινοῦ λόγος ιε** ^**ος**^, inc. ῞Ωσπερ ἐπὶ χολώδους ῥεύματος ὁ ἐρυσίπελας, des. ταῦτα ἐπίχεε· κ(αὶ) ἀναμαλάξας καλῶς χρῶ  *id.*, 7–138.^61^

**Blank pages:** 316v–317v.

**Annotations** (scribal)**:** (32) text erased and corrected – (65) variant reading: ‘Γρ(άφεται) βῆχα κ(αὶ) πνιγμόν, λύγγα τε κ(αὶ) κατάρουν’ – (243v, 314r) additions to the text in the outer margin.

**Handwriting:** Humanist hand similar to those in Oxford, Corpus Christi College, MSS 97–99.^62^

**Annotations** (non-scribal)**:** (flyleaf Ιν) ‘τα δε ἐνεστι εν τῶ ενατω λογω’.

**Paper:** folded in 2°; chain distance 31 mm; watermarks very similar to Piccard 119080, 119149, 119155 (anchor), attested in 1509–1511 and to Piccard 123855–8 (crossbow), attested in 1549–55.

**Catchwords** (scribal)**:** *Ii3*, vertical.

**Quire signatures** (non-scribal)**:** Latin letters *Ie1* ‘pp’ (1r) through ‘zz’ (p. 144), then ‘aaa’ (f. 82r) through ‘zzz’ (f. 300r), then ‘Aaaa’ (f. 308r).

**Quires:** 1 (i), 2 (iii), 1 (iv), 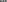 (p. 63), 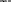 (f. 251), 8 (f. 259), 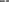 (f. 299), 8 (f. 307), 10 (f. 317).

**Binding:** *corio russico. –* Flyleaves: (i–iii) chain distance 26 mm, no watermarks. – (iv and 317) same paper as in the MS.

**Marks of ownership:** (flyleaf I) ‘Ex Bibliotheca Askeviana / P ii Art 546 / J. Sims’.

**Provenance:** Anthony Askew (1722–74), London; [his sale, G. Leigh and J. Sotheby, London, 15 March 1785, lot 546]; purchased by James Sims (1741–1820), London, in 1785; purchased by the London Medical Society in 1802; purchased by the Wellcome Library in 1984.

Bibliography: *Bibl. Askev.*, 34 (no. 546); *Cat. Med. Soc*. 1803, 4; *Cat. Med. Soc*. 1829, 5; Daremberg, 163; Dawson, 112; Nias, liv; Diels, II.6; Nutton, 24; Nutton and Zipser, 261; Touwaide, 539.
Georgios A. Costomiris, ‘Études sur les écrits inédits des anciens médecins grecs. Deuxième série: L’Anonyme de Daremberg, Métrodora, Aétius’, *Revue des Études Grecques*, 3 (1890), 145–79: 172.

## 9. MS.MSL.112 (*olim* NN a 11 / Wf 8)

London, ca. 1732–63 AD (from watermarks).

Paper, 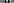, 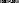 (foliated 1–217), linn. 30 [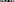]

John Zacharias Aktouarios, *Medical Epitome* ^63^ 3, first part of 6, 4, 5.

**Text: [1v–32v]** **Περὶ θεραπευτικ(ῶν) μεθόδ(ων) βιβλίον πρῶτον**, inc. ᾿Επειδὴ πᾶσα διδασκαλία καὶ μέθοδος, des. τὸ πᾶν τοῦ λόγου μέτρον συμπεραντέον  
Mathys, II.153.7–213.15. **[32v–78v]** **Τοῦ αὐτοῦ περὶ θεραπείας παθῶν καὶ τῶν ἔξωθεν φαρμάκων**, inc. ᾿Εδόκει διὰ βράχεων πάντων ἐπιμνησθῆναι βεβουλημένῳ, des. ὡς ἂν ἐν πᾶσι Θεοῦ διδόντος, ἄρτιος ὁ λόγος τελοίη. ΒΙΒΛΙΟϒ ΠΡΩΤΟϒ ΤΕΛΟΣ   *id.*, II.433–526.10. **[79v–139v]** **Τοῦ αὐτοῦ περὶ θεραπευτικῆς μεθόδου τῶν κατὰ μέρος παθῶν βιβλίον δεύτερον**, inc. ῾Η μὲν παροιμία φησί, χελώνης κρέα, ἢ φαγεῖν, ἢ μὴ φαγεῖν, des. προσῆκον ὠδὶ καὶ τοῦτον ἐνταῦθά πη συγκαταπαύειν. ΒΙΒΛΙΟϒ ΔΕϒΤΕΡΟϒ ΤΕΛΟΣ   *id.*, II.213.16–316. **[140v–217v]** **Τοῦ αὐτοῦ περὶ συνθέσεως φαρμάκων λόγος Α΄**, inc. ῎Ηδη σοι καὶ τὸν ἐπὶ τοῖς τέσσαρσι βιβλίοις ἐπιτίθεμεν λόγον, des. τῶ μετὰ τοῦτον δὲ προσθήσομεν ὅσα δοκεῖ λείπειν πρὸς τὸ τῆς ὑποσχέσεως ἄρτιον   *id.*, II.317–432.^64^

**Note:** This MS was copied directly from Wellcome MS.MSL.52 (203r–332v). Several labels bound among the pages of MS.MSL.112 contain handwritten notes in which the scribe corrects errors he had made while copying from MS.MSL.52 and refers directly to his exemplar. For example, the label for f. 166v in MS.MSL.112 reads ‘βιβλίου – φύλλα  – 309 – φύλλου ἀριστερὸν – εἰς τὸ τέλος τοῦ φύλλου – (ζαδούαρ) δέν το ἔγραψα ἄκρυ ἀριστερά’. Indeed, the last line on f. 308v in MS.MSL.52 reads ‘ζιγγιβέρεως· ζαδούαρ· ἀνίσου· γεντιανῆς’; cf. MS.MSL.112, 166v: ‘ζιγγιβέρεως· ἀνίσου· γεντιανῆς’.

**Paper:** folded in 2°; chaindistance 25 mm; pro patria watermarks with gr countermarks similar to Gaudriault 314.1–2.^65^

**Marks of ownership:** (flyleaf II) ‘Ex Bibliotheca Askeviana / Part ii. Art. 541 / J. Sims’.

**Provenance:** Anthony Askew (1722–74), London; [his sale, G. Leigh and J. Sotheby, London, 15 March 1785, lot 541]; purchased by James Sims (1741–1820), London, in 1785; purchased by the London Medical Society in 1802; purchased by the Wellcome Library in 1984.

Bibliography: *Bibl. Askev.*, 33 (no. 541); *Cat. Med. Soc*. 1803, 3; *Cat. Med. Soc*. 1829, 4; Dawson, 114; Nias, liv; Diels, II.110; Nutton, 24; Nutton and Zipser, 261; Touwaide, 538; Bouras-Vallianatos, *op. cit.* (MS.MSL.52) 370–1, 400.

## 10. MS.MSL.114 (*olim* AA c 2 / Wf 15)

Byzantine Empire, ca. 1335–45 AD (from watermarks).

Paper, 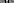, 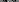 (foliated 1–128, 129–201), linn. 36 [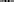], unclear ruling.

Paul of Aegina, *Medical Epitome* (TLG 0715.001).

**Text: [1r–v]** **᾿Εν** […], inc. Τοῦνομά μοι Παύλος πατρὶ Αἴγινα πολλὰ μογίσας <π>άσ[α]ν ἀκεστορίην βίβλον ἔτευξα μί(αν) οὐχ᾿ ὡς τῶν παλαιὁτέρων ἐν τοῖς κατὰ τ(ὴν) τέχνην, des. ἐλάχιστα μόν(ον) ἐκ τ(ῶν) ἐκεῖνου τέτακται 
Heiberg, I.3–6.8. **[1v]** Table of Contents: no title, inc. α΄ Περὶ τῶν συμβαινόντων παθῶν, des. λε΄ περὶ ἀφροδισιῶν:  *id.*, I.6.9–6.47; **[2r]** Inc. λς΄ πρὸς τοὺς μὴ δυναμένους συνουσιάζειν, des. ρ΄ διοκλέους ἐπιστολὴ προφυλακτική  *id.*, I.6.48–7.77. **[2v–19v]** **Περὶ τῶν συμβαινόντων ταῖς κυούσαις. καὶ πῶς αὐτὰς διαιτητέον**, inc. Τῶν συμπιπτόντων ταῖς κυούσαις, des. εἰς τροπὰς χειμερινὰς ἡμέραι με΄  *id.,* I.8.1–72.12. **[19v–20r]** Table of Contents: **᾿Αρχὴ τοῦ δευτέρου βιβλίου**, inc. ᾿Εν τούτῳ τῷ βιβλίῳ δευτέρῳ τῆς ὅλης ὑπάρχοντι πραγματείας, des. ἑλκώσεως  *id.,* I.73.1–74.36. **[20r–32v]** **᾿Εκ τῶν Γαληνοῦ**, inc. Ποίαν ἀρχὴν, des. μετὰ σιδίων: τέλος τοῦ δευτέρου βιβλίου  *id.,* I.74.1–126.29. **[36r]** Table of Contents: **Κεφάλαια τοῦ τρίτου βιβλίου**, inc. ᾿Εν τούτῳ τῷ βιβλίῳ τρίτῳ τῆς πραγματεί(ας), des. παρονυχίας  *id.,* I.127.1–128.55. **[36v–88r]** **Περὶ τῶν κατὰ τὰς τρίχας παθῶν**, inc. Καθάπερ τῶν φυτῶν, des. φαρμάκου: τέλος τοῦ τρίτου βιβλίου *id.,* I.129.1–314.25. **[88v]** Table of Contents: **᾿Αρχὴ τοῦ τετάρτου βιβλίου**, inc. ᾿Εν τούτω τῷ βιβλίῳ τετάρτῳ ὑπάρχοντι τῆς ὅλης πραγματεί(ας), des. νη΄ περὶ δρακοντίων  *id.,* I.315.1–316.13. **[88v–108r]** **Περὶ ἐλέφαντος**, inc. ᾿Ορθῶς οἶμαι τὸν Καππαδόκην ᾿Αέτιον εἰπεῖν, des. ἐπιμελεία δοκιμαζέσθω  *id.,* I.317.1–388.24. **[108v]** Table of Contents: **᾿Αρχὴ τοῦ ἕκτου βιβλίου**, inc. ᾿Εν τούτῳ τῷ βιβλίῳ πέμπτῳ ὑπάρχοντι τῆς ὅλης πραγματεί(ας), des. (καὶ) ψυχροῦ ὕδατος  *id.,* II.3.1–4.31. **[108v–118r]** **Προφυλακτικὰ πάντων κοινῇ τῶν ἰοβόλων**, inc. Τὴν περὶ τῶν ἰοβόλων ζώων, des. τὸν ἐπηρτημένον κίνδυνον: τέλος τοῦ πέμπτου βιβλίου  *id.,* II. 5.1–41.21. **[118r–v]** Table of Contents: **᾿Αρχὴ τοῦ ἕκτου βιβλίου**, inc. ᾿Εν τούτῳ τῷ βιβλίῳ ἔκτῳ τῆς ὅλης πραγματεί(ας) ὑπάρχοντι, des. κατάγμασι γινομένης ἐξαρθρήσε(ως)  *id.,* II.42.1–44.44. **[118v–151r]** **᾿Αρχὴ προοιμίου τῶν χειρουργουμένων**, inc. Τὸν περὶ τῶν χειρουργουμένων λόγον, des. ἁρμόζοντα δεῖ ποιεῖσθαι χειρισμὸν: τέλος τοῦ ἕκτου βιβλίου  *id.,* II.45.1–183.10. **[151r]** Table of Contents: **᾿Αρχὴ τοῦ ἑβδόμου βιβλίου**, inc. ᾿Εν τούτῳ τῷ βιβλίῳ ἑβδόμῳ καὶ τελευταίῳ τῆς ὅλης ὑπάρχοντι πραγματεί(ας), des. περὶ σταθμῶν καὶ μέτρων  *id.,* II.184.1–46. **[151r–197r]** **Περὶ τῶν ἀπὸ τῶν γευστῶν ποιοτὴτων δηλουμένων κράσεων**, inc. Οὔτε ἐξ ὀσμῆς, des. ἀντὶ ὠκιμοειδοῦς, ἡδύοσμον ἄγριον: ἀντὶ ὤχρας, ἀντὶ τοῦ ὠοῦ τὸ πυρόν, μέλι ἢ ἕψημα: ἀντὶ ὠοῦ τὸ λευκόν, γάλα γυναικεῖον  *id.,* II.185.1–408.10. **[197r–v]** **Περὶ μέτρων**, inc. Πολλῶν καὶ σχεδὸν ἀπείρων ὄντων τῶν μέτρων, des. χοῦς ἀττικός, ξε΄ στ΄: ψὼρ ὁ ἐστι κόρος, μοδ΄ λ΄   *id.,* II.408.11–411.20.^66^

**Annotations** (scribal)**:** (36r, 68r, 77r, 97r, 122r, 140v, 146r, 184v, 187r) additions to the text in the outer margins – (78r) variant reading: ‘Γρ(άφεται) μετὰ τόνου φέρεται’.

**Handwriting:** attributed by Nigel Wilson to Georgios Chrysokokkes (RGK III 126).

**Text added** by several different hands**: [1r–v]** Lost text replaced. **[2r, lower margin]** Inc. Περὶ μαλαφράτζας θεραπεία μίνετε τοῦτο τὸ πάθος ἀπὸ φλέγματος, des. κ(αὶ) σπέρμα ραφάνου. **[59v, outer margin]** Inc. Τω αίμα εκ πολλῶν τόπων ἀναγεται, des. και ἀνατρίβειν ἐλαιω παλεώ. **[178r, lower margin]** Inc. ᾿Εκ τοῦ φίλονος ἀντίδοτος ποιοῦσα προ κουλικοὺς, end lost. **[195r, outer margin]** Inc. ῎Εδοκα τον ἀρ^χ^ δουκα τζουλέπει κεπαρξεν, des. γάστρι ἄφοβον μερί. **[195r, lower margin]** Inc. Εἰς και [rasura] τιμ(ὸν) καὶ εἰς πόνον, end lost. **[195v, outer margin]** Inc. ῎Οντα ο στωμαχὸς του αν(θρωπ)ου, des. καὶ πλάκοσε τον στωμαχω. Inc. ΄Αλον να παρης κηδόνι, des. ροδοσάχαρι κ(αι) πεινε το νήστης καργα. Inc. [΄Α]λον· να παρης ασβεστην ζοτανὸν, des. εις το στωμαχω. Inc. Αλον επαρε σκειλοσκατα ξερᾶ, des τα σκειλοσκατα. Inc. Εις πονον στομαχου να παρης χαλβανη, des. εις τον [στο]μαχον. **[195v, lower margin]** Inc. Οντα κριώσοι ο στομαχος, des. βάλε άλον. Inc. Οντα το φάγι ο σκήλος, des. αστου να παρι. **[196r, outer margin]** Inc. Οντα τινὰ σφαζυ, des. εἰς τὸν πόνον. Inc. ῞Οντα κατάβυ, des. χηλον κ(αὶ) διάχριε. Inc. Καθαρτικὸν στομαχιακον, des. εξαφρισμεν[(ον)] καλα. **[196r, lower margin]** Inc. ῞Οντα σκοτοδί ὁ ἄν(θρωπ)ος, des. και κοψε το και πασυζέ το. **[196v, upper margin]** Inc. Οντα, des. κατακρειμα. **[196v, outer margin]** Inc. ᾿Αρχή να παρης, des. να τὸ πεῖ ἀν(θρωπ)ος. Inc. Εις τζέρμα, des. αλιφε κ(αὶ) τρίβε τα. Inc. Εἰς στόμαχον οντα εχοι, des. το στόμαχον του. **[196v, lower margin]** Inc. Εἰς λιγωρικον, des. ἐφόρβιον. **[197r, upper margin]** Inc. Ελέβορος λέγετε, des. ροφανιδον. **[197r, outer margin]** Inc. Περὶ ρέματος μεν, des. ζεστα. Inc. Εἰς καρδιακο να παρης βοτανη, des. το κειδώνιο νιστης. Inc. Επαρον πιτερα, des. νηστην. **[197r, lower margin]** Inc. ῾Ο κόσμος κεχαραγμένος, des. ψυχρᾶ κ(αί) ὑγρᾶ. **[197v, upper margin]** Inc. Σέσελε πλατικειμενο, des. δια τα μερμίκεια. **[197v, outer margin]** Inc. Περὶ πόνον, des. διαλίνου. **[197v, lower margin]** Inc. Λαβὼν χρυσὸν καλόν, ρίνισον ρινίω ψιλῶ, des. τὸ αὐτὸ ποιεῖ (καὶ) εἰς τ(ὴν) ἀργυρογραφίαν εστωδε. Inc. Ρἴνισμα χρυσὸν ἐξάγ΄ α΄, des. τράργακανθον τὸ ἀρκοῦν. γράφε. Inc. Χρυσον ρἴνισμα, des. κόμίδι λευκου, οὐγγ α΄. Inc. Εἰς τὰ μερμίκεια, des. και βράση τα κ(αὶ) δὸς ποιεί. **[198r]** Inc. Περι ωνιχας, des. κατ τα φυλ καταπλάσμεν. Inc. Εσωθεν τον οφθαλμον διπλα τρίχας, des. και ἀφάνιζον. Inc. Λιβάνου αφονήτρου, des. χρω. Inc. Περι παρωτίδον, des. επειτιθέμενος. Inc. ΄Αλον επάρε χιμονικον, des. επειτα ας το φαγ[?] αστεν(ειν) να κηνισοι. Inc. Περὶ στομάχου επαρε χαλβαν(ην), des. εις τον στομαχο. **[198v]** A number of recipes by the same hand, eg. inc. Περι λευκόματα οφθαλμου, des. θαμαυσ(ειν) ὀφθαλμούς. Inc. Εἰς βύχα, des. μετὰ ἀμιγδαλεος. Inc. ῎Αριστον προς οφθαλμους, des. χρίε. Inc. ᾿Αριστον πρὸς οφθαλμον, des. χρίε. Inc. ῞Ετερον εἰς οφθαλμους, des. καταπλατε κυκλος πιή. Inc. Εις πονον οματίου, des. κ(αὶ) θές. Inc. Οφθαλμικα, des. καλιτερον. Inc. Εις ψωραν αλογου ει αλου ζωου, des. αλιφε τοῦτο. Inc. Περι σταφηλὶς, des. οφακειον χυλ(ὸν). **[197v]** Addition to the main text, inc. ῾Η λίτρα εχοι ηΓο΄ ςΓΓ΄ ς΄  ο ςΓΓ κερατα κδ΄, des. κοτιλίου ςΓΓ ς΄.

**Annotations** (non-scribal)**:** (16r, 18v, 20r, 20v, 22v, 26r, 29v, 32r, 36r, 37v, 39v, 40r, 43v, 44v, 48r, 48v, 49r, 49v, 50r, 51r, 52r, 52v, 53r, 53v, 54v, 55r, 55v, 56r, 57r, 58r, 58v, 62r, 62v, 63v, 65r, 67v, 68r, 68v, 69v, 73r, 74r, 75v, 76r, 76v, 91r, 92r, 93r, 94r, 94v, 97v, 100r, 101r, 101v, 102v, 104r, 106v, 109v, 151v, 152r, 152v, 153r, 153v, 154r, 154v, 155v, 156r, 156v, 157r, 157v, 158r, 158v, 159r, 159v, 160r, 160v, 161r, 161v, 162r, 162v, 163r, 163v, 165r, 165v, 166r, 166v, 167r, 167v, 168r, 168v, 169r, 169v, 170r, 170v, 171r, 171v, 172r, 172v, 173r, 173v, 174r, 174v, 175r, 176r, 177r, 177v, 178v, 179r, 179v, 180r, 180v, 181r, 182r, 183r, 183v, 185r, 185v, 186r, 189v, 190v, 193r, 193v) mostly short phrases on single words, of four kinds: efficacy phrases, indication of contents or, less frequently, short explanatory phrases and variant readings, eg. (18v) ‘ἀριστων ἐπειστωλὴν ἀντιγώνω βασιλή περὶ καιφαλής᾿, (22v) ‘πρὸ τῆς συστολῆς. ἑτέραν δὲ τ(ὴν) μετὰ τ(ὴν) συστολ(ήν)’, (26r) ‘ἄριστον᾿, (36r) ‘κ(ε)φαλ(α)ιω με΄. ήρεσε να το κο^ν^[?] κ(αὶ) ου εμπουρου να κατουρισου’, (39v) ‘χρίσημον κεφαλαλγια’, (49v) ‘τω λεγομενον δικεντιον και το καλουμενον στρατιωτικον’.

**Paper:** folded in 2°; chain distance 43 mm; watermarks (1) very similar to Mošin and Traljić 6947, 6949, 6956 (*saucisson*), attested in 1338–50 and (2) identical with Mošin and Traljić 5791 (*licorne*), attested in 1339–44.^67^

**Added leaves:** (ff. 1, 198) paper folded in 2°; chain distance 35 mm; watermarks similar to Piccard 122415 (scissors), attested in 1457.

**Quires:** 1 (flyleaf i), 2 (ii–iii), 1 (f. 1), 4 (f. 5), 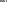 (f. 7), 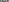 (f. 190), 1 (f. 191), 6 (f. 197), 1 (f. 198), 2 (i–ii), 1 (iii).

**Binding:** *corio russico*. – Flyleaves: chain distance 25 mm, no watermarks.

**Marks of ownership:** (5v, 20r, 36v, 88v, 117v) ‘Γεράκης’, (151v) ‘λεξικὸν των βοτάνον’ – (flyleaf II) ‘Ex Bibliotheca Askeviana P.ii.Art. 404 / J. Sims / A A Ac c 2 / WE15’ – (flyleaf III) ‘W. H. 18’ – (back pastedown) label inscribed ‘O.g.24’.

**Provenance:** (Manuel Kantakouzenos?) Gerakes (cf. Wellcome MS.MSL.52) – Anthony Askew (1722–74), London; [his sale, G. Leigh and J. Sotheby, London, 10 March 1785, lot 404]; purchased by James Sims (1741–1820), London, in 1785; purchased by the London Medical Society in 1802; purchased by the Wellcome Library in 1984.

Bibliography: *Bibl. Askev.*, 23 (no. 404); *Cat. Med. Soc*. 1803, 3; *Cat. Med. Soc*. 1829, 4; Daremberg, 164; Nias, lvi–lvii; Diels, II.77; Dawson, 115–6; Nutton, 24–5; Nutton and Zipser, 261; Touwaide, 538–9.
Brigitte Mondrain, ‘La lecture et la copie de textes scientifiques à Byzance pendant l’époque paléologue’, in Giuseppe De Gregorio and Maria Galante (eds), *La produzione scritta tecnica e scientifica nel Medioevo: libro e documento tra scuole e professioni* (Spoleto: Centro italiano di studi sull’alto medioevo, 2012), 607–32: 632.
Gabrielle Lherminier, ‘Edition critique et traduction annotée du livre V des “Epitomae Medicae” de Paul d’Egine’ (unpublished PhD thesis: Paris, École Pratique des Hautes Études, 2013), 249–50.

## 11. MS.MSL.124 (*olim* AA d 6 / Wf 16)

England, c. 1650 AD (from watermarks).

Paper, 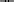, 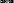 (foliated 1–77), linn. 45 [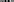].

John Zacharias Aktouarios, *On Urines* (TLG 3188.002).

**Text: [1r]** Table of Contents: **ΑΚΤΟϒΑΡΙΟϒ**, inc. Περὶ διαφορᾶς οὔρων, des. περὶ προγνώσεως ἐκ οὔρων τὸ Β. **[1v]** Table of Contents: **ΑΚΤΟϒΑΡΙΟϒ Περὶ διαφορᾶς οὔρων**, inc. Τάδε ἔνεστιν ἐν τῷ περὶ διαφορᾶς οὔρων λόγῳ, des. περὶ ἀνωμαλίας τῶν παρυφισταμένων περὶ τάξεως κ(αὶ) ἀταξίας ἐκάστου τούτων. κεφ΄ κα 
Leone, xv–xvi; Georgiou, 398–400. **[2r–13r]** **᾿Ακτουαρίου περὶ διαφορᾶς οὔρων**, inc. Πάλαι μὲν ἴσως φιλοτιμίας ἔργον τιθέμενος, des. ἀρχῆς τῶν μετὰ τοῦτον λόγων ἀψώμεθα. τοῦ περὶ διαφορᾶς οὔρων βιβλίου τέλος 
Ideler, II.3–31.19; Georgiou, 401–56. **[13v]** ΑΚΤΟϒΑΡΙΟϒ ΠΕΡΙ ΔΙΑΓΝΩΣΕΩΣ ΟϒΡΩΝ ΤΟ Α᾿ **[14r]** Table of Contents: **Ακτουαρίου περὶ διαγνώσεως οὔρων**, inc. Τάδε ἐνεστὶν ἐν τῷ α περὶ διαγνώσεως οὔρων λόγῳ, des. περὶ διαγνώσεως φαιῶν πελιδνῶν καὶ μελανῶν οὔρων. κεφ΄ κ 
Leone, 59.8–60; Mathys, I.161–162.4. **[14v–23r]** **᾿Ακτουαρίου περὶ διαγνώσεως οὔρων τὸ α**, inc. ᾿Επειδὴ τὸ μανθάνειν ἐθέλειν τὰς ἐκ τῆς τῶν οὔρων σημειώσεως, des. ἣν πάντα τρόπον ἀκριβῶς ἀσκεῖν προσήκει πειρᾶσθαι. τοῦ βιβλίου περὶ προγνώσεως οὔρων τὸ α, τέλος 
Ideler, II.31.20–52. **[23v]** ΑΚΤΟϒΑΡΙΟϒ ΠΕΡΙ ΔΙΑΓΝΩΣΕΩΣ ΟϒΡΩΝ ΤΟ Β. **[24r]** Table of Contents: **Ακτουαρίου περὶ οὔρων διαγνώσεως τὸ Β**, inc. Τάδ᾿ ἔνεστι ἐν τῷ Β περὶ διαγνώσεως οὔρων, des. ὅτι δεῖ τὸν ἀκριβῶς ἐπισκέπτεσθαι βουλόμενον καὶ τὴν περὶ σφυγμῶν πραγματεῖαν ἀκριβῶς ἐπιστάσθαι. κεφ΄ ις  Leone, 105–6; Mathys, I.193–194.16. **[24v–34v]** **᾿Ακτουαρίου περὶ διαγνώσεως οὔρων τὸ Β**, inc. Τὸ διαγινώσκειν τῶν παθῶν τὰ κατέχοντα πεφυκὸς, des. συμπεράναι λόγῳ ἀληθίας πιστούμενα. ἀκτουαρίου περὶ διαγνώσεως οὔρων τὸ Β. τέλος. 
Ideler, II.53–78. **[35r]** ΑΚΤΟϒΑΡΙΟϒ ΠΕΡΙ ΑΙΤΙΩΝ ΟϒΡΩΝ ΤΟ Α. **[35v]** Table of Contents: **Ακτουαρίου περὶ αἰτίων οὔρων τὸ Α**, inc. Τὰδ᾿ ἐνεστὶν ἐν τῷ Α περὶ αἰτίων οὔρων, des. περὶ τῆς συμμεμιγμένων, ἤτοι ἀνομοιομερῶν παρυφισταμένων αἰτίας. κεφ΄ κα 
Leone, 161–2; Mathys, I.232.8–233.11. **[36r–47v]** **᾿Ακτουαρίου περὶ αἰτίων οὔρων τὸ Α**, inc. ᾿Επειδὴ τῷ περὶ τινὸς αἰρουμένω γραφεῖν ζητήματα, des. τοῖς δὲ σπουδάζουσιν οὐκ ἀγεννὴς ἔσται μέθοδος. τοῦ βιβλίου περὶ αἰτίων οὔρων το Α, τέλος 
Ideler, II.79–111.7. **[48r]** ΑΚΤΟϒΑΡΙΟϒ ΠΕΡΙ ΟϒΡΩΝ ΑΙΤΙΩΝ ΤΟ Β. **[48v]** Table of Contents: **Ακτουαρίου περὶ αἰτίων οὔρων τὸ Β**, inc. Τὰδ᾿ ἐνεστὶ ἐν τῷ Β περὶ αἰτίων οὔρων, des. περὶ αἰτίας οὔρων τῶν κατὰ ψυχρὰν διάθεσιν καιρίων πεπονθότων μορίων. κεφ΄ κ 
Leone, 229.8–230; Mathys, I.280.5–281.18. **[49r–60r]** **᾿Ακτουαρίου περὶ αἰτίων οὔρων τὸ Β**, inc. Οἷόν τι τοῖς φιλοθεάμοσιν συμβαίνειν εἴωθεν, des. ἤδη καὶ τοῦ περὶ προγνώσεως οὔρων. ακτουαρίου περὶ αἰτίας οὔρων Β. τέλος 
Ideler, II.111.8–144. **[60v]** ΑΚΤΟϒΑΡΙΟϒ περὶ προγνώσεως ἐξ οὔρων το Α. **[61r]** Table of Contents: **Ακτουαρίου περὶ προγνώσεως ἐκ τῶν οὔρων τὸ Α**, inc. Τὰ δὲ ἔνεστιν ἐν τῷ περὶ προγνώσεως ἐξ οὔρων, des. περὶ προγνώσεως τῆς ἐκ τῶν κατὰ τὴν στεφάνην ἐτεροειδῶν χρωμάτων 
Leone, 298–9; Mathys, I.329.4–330.9. **[61v–69v]** **᾿Ακτουαρίου περὶ προγνώσεως ἐξ οὔρων τὸ Α**, inc. ᾿Εδόκει τισιν τῶν τὰς προγνώσεις διαβάλλειν προχείρων, des. ὑγιαίνουσι τῆς πραγματείας σκοπήσας χρήσιμον 
Ideler, II.145–171.10. **[70r]** ΑΚΤΟϒΑΡΙΟϒ περὶ προγνώσεως ἐξ οὔρων το Β. **[70v]** Table of Contents: **Ακτουαρίου περὶ προγνώσεως ἐξ τῶν οὔρων τὸ β΄**, inc. Τὰδ᾿ ἔνεστιν ἐν τῷ περὶ προγνώσεως ἐξ οὔρων τῷ β΄ λόγῳ, des. πῶς εἰς τοῦτο τῆς πραγματείας προήχθημεν  Leone, 354; Mathys, I.367.20–368. **[71r–77v]** **᾿Ακτουαρίου περὶ προγνώσεως ἐξ οὔρων τὸ β΄**, inc. Τὸ τὴν πρόγνωσιν ἀσκεῖν τε καὶ ἐπιτηδεύειν, des. αὐτῶν δὲ τῶν λόγων ἀκριβής τις ἐπίσκεψις. ΤΕΛΟΣ 
Ideler, II.171.11–192.^68^

**Note:** Stavroula Georgiou has demonstrated that this MS was copied directly from Cambridge, Gonville and Caius College, MS 76/43.

**Paper:** folded in 2°; chain distance 27 mm; watermark very similar to Nostitz 760 (three hats with countermark NZ), attested in 1650.^69^

**Provenance:** Anthony Askew (1722–74), London; [his sale, G. Leigh and J. Sotheby, London, 15 March 1785, lot 542 (?)]; probably purchased by James Sims (1741–1820), London, in 1785; probably purchased by the London Medical Society in 1802; purchased by the Wellcome Library in 1984.

Bibliography: *Bibl. Askev*., 33 (no. 542); *Cat. Med. Soc*. 1803, 3; *Cat. Med. Soc*. 1829, 3; Daremberg, 158; Costomiris 10, 441; Nias, liv; Diels, II.109; Dawson, 120–1; Nutton, 24; Nutton and Zipser, 261; Touwaide, 539; Georgiou, *op. cit*. (note 34), 254–5, 332–4.
John Symons (ed.), *Books from the Library of the Medical Society of London: An Exhibition, 14 January to 3 April* (London: Wellcome Institute, 1985), 9.

## 12. MS.MSL.126 (*olim* AA c 4 / Wf 6)

Cambridge, 1648 AD (from note on f. 1r).

Paper, 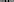, 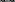 (foliated 1–161, 162–245, 245–302, 303–307, 308–314, 315–386, 387–409, 410, 411–474).

Oribasios, *Medical Collections* (TLG 0722.001), Books 1–10 and 14.

**Text: [2r–13r]** Table of Contents: **Α δένεστιν εν τῷδε τῷ βιβλίῳ. κεφάλαια τοῦ α΄ βιβλίου τῶν ᾿Οριβασίων συναγωγῶν**, inc. ᾿Εκ τῶν Γαληνοῦ περὶ ἀρετῆς κ(αὶ) κακίας τῶν δημητριακῶν σπερμάτων. α΄, des. ὅσα ξηραίνει τῆς τετάρτης ἀποστάσεως. κη΄   Raeder, I.1.3, 28–9, 66, 92, 110, 154, 193, 246, I.2.3, 39, 181.45. **[14r–406r]** **᾿Οριβασίου ἰατρικῶν συναγωγῶν βιβλίον α΄. πρὸς ᾿Ιουλιανόν**, inc. Τὰς προσταχθείσας ἐπιτομὰς παρὰ τῆς σῆς θειότητος ἀυτοκράτορ ᾿Ιουλιανέ, πρότερον, ἕνεκα διετρίβομεν ἐν Γαλατίᾳ, des. κ(αὶ) κοιλιακῶν διαθέσεων κ(αὶ) ποδαλγικῶν κ(αὶ) ἀρθριτίδων, ὅταν γε μήπω σύστασις ἢ πῶρος. τέλος τοῦ βιβλίου ι΄ͺ  *id.*, I.1.4–300, I.2.4–79. **[407r–474r]** **ΒΙΒ ΙΔ. ᾿Οριβασίου συναγωγῶν ἰατρικῶν**, inc. Οὔτε τὰς ἰδέας ἁπλῶν φαρμάκων πρόκειται νῦν ἡμῖν γράφειν Διοσκουρίδου καλῶς αὐτὰς διδάξαντος, des. ἅπαντα γὰρ ταῦτα λεπτυντικῆς ἐστι δυνάμεως, ὥσπερ αὖ τἀνάντια παχυντικῆς. τέλος τοῦ βιβλίου ιδ΄  *id.*, I.2.183–237.^70^

**Note:** This manuscript was copied directly from Cambidge, Saint John’s College, MS A.6.

**Marks of ownership:** (1r) ‘Vita Ovidii Oribasii describitur a Eunapium graecae, qui Eunapius cum Dionysio Laertio in 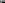 συνδεον έεται’ – (1r) ‘Hoc Manuscriptum Transcribebatur ex Copiae Bibliothecae St Johannuis Collegii: Acad. Cantab. Atque Reuisum fuit secundum Copiam Anno Domini 1648. Robertus Waideson, Medicinae Doctor’ – (1r) ‘Ex Bibliotheca Askeviana / P. ii. Art. 588 / J. Sims’.

**Provenance:** probably commissioned by Robert Wadeson, Cambridge – Anthony Askew (1722–74), London; [his sale, G. Leigh and J. Sotheby, London, 16 March 1785, lot 588]; purchased by James Sims (1741–1820), London, in 1785; purchased by the London Medical Society in 1802; purchased by the Wellcome Library in 1984.

Bibliography: *Bibl. Askev*., 37 (no. 588); *Cat. Med. Soc*. 1803, 149; *Cat. Med. Soc*. 1829, 232; Daremberg, 158; Nias, lv–lvi; Diels, II.71; Raeder, *op. cit*. (note 70), Vol. I.1, v; Dawson, 122–3; Nutton, 24; Nutton and Zipser, 261; Touwaide, 539.

## 13. MS.MSL.135 (*olim* H Hi 1)

Ottoman Empire, ca. 1525–65 AD (from watermarks and handwriting style).

Paper, 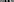, 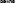 (foliated 1–162),^71^ linn. 22 [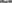], 20D1.

**[1r–86r]** Theophanes Chrysobalantes (Nonnos), *Medical Epitome.* ^72^ **[86r–96r]** *Idem*, *On Diet* (TLG 0721.010, 0721.017).^73^ **[96r–110r]** *Idem*, *Synopsis on Composite Drugs*.^74^ **[110v–154v]** Symeon Seth, *On the Capacities of Foodstuffs* (TLG 3113.002).^75^

**Text: [1r–3v]** Table of Contents: no title, inc. α [᾿Α]πὸ πτουσῶν τριχῶν, des. σν Περὶ κεραυνῶν  
Bernard, I.xxiii–xxxi.7. **[4r–86r]** **Περὶ πιπτουσῶν τριχῶν**, inc. ᾿Απὸπίπτουσιν αἱ τρίχες τῆς κεφαλῆς· καὶ διὰ ρεότητα τοῦ δέρματος, καὶ διὰ στέρησιν τῆς ποιούσης αὐτὰς ὑγροτητος· ἄλειφε γοῦν ἀλόην μετὰ οἴνου μέλανος αὐστηροῦ τὴν κεφαλήν· ἢ σμύρναν κ(αὶ) ὀπιανὸν μετὰ οἴνου καὶ μυρσίνου ἐλεου, des. γίνεται κεραυνός· ὅταν γένηται ρήξις καί σχίμα τῶν νεφρῶν, des. ἐν δὲ τοῖς διαἰτωμένοις ἐν ὕδασι φώκη· ταῦτα ἀποτρέπουσι καὶ ἀπόδιοὥκουσι κερανούς  
Bernard, I.8.3–462, II.2–288. **[86r–87r]** **Τοῦ αὐτοῦ περὶ εὐχύμων κ(αὶ) κακοχύμ(ων) κ(αὶ) τῶν λοιπῶν**, inc. Καὶ τοῦτο ἐπίταγμα Κωνσταντίνε θειότατε καὶ μέγιστε αὐτοκράτορ· τῆς σῆς προνοίας καὶ τῆς μεγαλοφυοὺς ἐπινοίας καὶ φρονήσεως, des. καὶ τελευτέαν πασῶν, τὴν λεπτύνουσαν καὶ παχύνουσαν  
Felici, 67–8. **[87r–96r]** **Περὶ εὐχύμων**, inc. Εὐχυμώτατον εστι· σχεδὸν παρα τὰ ἄλλα πάντα, τὸ ἄριστον δὲ ἐστι· τὸ τῶν ὑγιαινόντων ζώων, des. ἄρτοι πλυτοί· ἄμυλον· καὶ τὰ κρασερὰ σταφύλια  
Ermerins, 237.23–275; Ideler, II.257–268.29. **[96r–110r]** **Σύνοψις ἐν ἐπιτόμῳ περὶ τῶν βοηθημάτων καὶ τοῦ τρόπου τῆς δόσεως αὐτῶν· μετὰ τῶν ἰδί(ων) προπορημάτων· ἣ ως ἄπερ δεῖ πρότερον διδόναι πίνειν καὶ προκαθαίρειν δι᾿ αὐτῶν· εἶτα καὶ τὰ βοηθήματα διδόναι· ὁμοίως καὶ περὶ τροχίσκων καὶ ἐλιγμάτων· πρὸς τοῦτοις δὲ· καὶ περὶ ἐλαίων καὶ εμπλάστρων· καὶ λοιπῶν τῶν εἰς θεραπίαν διἀφόρων νοσημάτων σὺν τεινόντων**, inc. ῾Ο περὶ τῶν ἀντιδότων· καὶ ἐλαίων· καὶ ἐμπλάστρων λόγος, des. **Τὸ δι ἀσάντυγος**, ᾿Επὶ βοθρί(ων) καὶ βαθέων τραυμάτων ἐστὶ θρεπτικόν etc, ἀξουγγίου χηνὸς· ἀνὰ οὐγγιῶν δύο· ἀξουγκίου χοιρινοῦ λίτρας πέντες. **[110v–154v]** **Τοῦ Μαίστορος περὶ τρόφῶν δυνάμεων**, inc. Τὰ αμήγδαλα· σύμμετρα εἰσὶ τη θερμότ(η)τ(ι)· καὶ τρόφημα ἱκανός, des. καὶ τὰ περὶ ταῦτης ἐκ τοῦ περὶ ἐκείνων διἀγνοσθήσεται  
Langkavel, 20.16–21.20, 22.10–21, 20.7–15, 23.9–21, 24.3–25.2, 23.23–24.2, 26.1–28.6, 29.23–8, 30.11–31.6, 34.1–39.23, 40.9–23, 42.23–43.11, 90.11–92.13, 43.19–45.12, 48.4–55.4, 57.14–25, 67.9–12, 58.1–16, 56.19–57.18, 58.19–59.14, 60.6–13, 61.8–62.24, 63.9–67.19, 71.14–6, 68.8–71.6, 71.17–73.3, 73.12–74.3, 73.4–11, 74.4–80.3, 81.5–11, 86.21–87.4, 80.24–81.4, 80.4–23, 84.3–85.22, 87.5–88.12, 85.23–86.20, 83.15–84.2, 81.12–83.14, 88.13–93.25, 100.5–12, 94.1–100.4, 100.13–103.7, 103.25–104.3, 103.8–103.24, 104.12–107.10, 108.1–22, 107.11–24, 108.23–109.10, 111.10–26, 109.11–111.9, 112.1–123.15, 18.4–20.6, 123.16–125. **[154v]** Scribal colophon: Δόξα σοι ὁ Θ(εὸ)ς ἡμῶν δόξα σοι.^76^

**Note:** According to Barbara Zipser, MS.MSL.135 is a sibling of Florence, Biblioteca Medicea Laurenziana, MS Plut. 75.6.

**Blank pages:** 156r–161v, 162v.

**Handwriting:** unidentified post-Byzantine hand.

**Recipes added** by several different hands**:** **[154v]** Τὴν δρακοντήαν· ἔπαρε τὴν ρήζα της· κ(αὶ) ἐνε καλή· σε παρανὴ i δες, Τὸ εὐπατόριον fistula lachrymata sanauit epotii in decoctum. **[154v]** Inc. ᾿Ενα βοτάνη που το λέγουνε το παπατα [?], des. ἔμπλαστρο κ(αὶ) πὶ εἴς [?] αυτου μετα [end lost in trimming]. **[155r]** Inc. ῎Ενα βωτάνη· βρήσκετε· στανπέληα μέσα, des. να πίνη απο δησουρήαν. **[155r]** Κ(αι)νον παλεοῦ· την σελήνην· καὶ δος το να στεστεν [?]. **[162r]** **Κατασκευή τής αγριωκρομίδης**, inc. Πρότον την κόφτης εις λεπτά κ(αὶ) τήν βάζις σε νερον κ(αὶ) πάλιν έως πεντάξι νερά, des. καὶ πάρνις όσα ποταρίδη βράδη κ(αὶ) τάχιστα βύχα οπ[ου] ερχετε απο κρίο. Inc. ᾿Οφελούν δε κ(αὶ) τὰ κεδροκούκουτζα, des. κ(αὶ) ρίχτης το αυτόν νερόν κ(αὶ) απ αυτώ άς πήνει ὁ πάσχον αποτετόν βύχαν.

**Annotations** (non-scribal)**:** marginal notes by various hands, usually in the vernacular and mainly of three kinds: short explanations in the form of synonyms, indications of contents, details on the administration of certain drugs, eg. (33r) ‘ὅταν (…) τὸ βότανον (…) ἐπαρα το φίλον (…) καὶ βάλε το (…)’, (40v) ‘ἀμάλα, λέγετε, ὁ ἀγρηοπίγανος’, (43v) ‘ἀνθρακί(ας)· λέγουν τὰ κάρβοα’, (46v) ‘Ση(μείωσαι)’, (55r) ‘ηγουν αχονεψία στομαχοῦ’, (105v) ‘ἀνακαρδῶν’ – (82v) drawn hand pointing to chapter ‘σλη΄ Περὶ κνησμονῆς’ – (87r) ‘Τὸ γάλα’ added as a title – (105r) ‘μυρολαμβάνου ’ crossed out and corrected to ‘μυροβαλάνου’ – (151v, 152v) ‘εως της ημερας της ζοες επεθυμηθυον’, ‘υδορ με γενα η γη θρεφη με φοσφορος βασιλης γηαρχοτες ωλη με αγαπουσι κι οταν ασεβο ες την μητερα μου γευγομε του θανατο μου’, ‘ετος ετουτο’, ‘ο κ(υρι)ε μου κ(αι) Θεε μου’ – (155v) ‘Amico mio paga volto ecca[?] choritio[?]. Ama i dio non fallire far pur bene e lasca dire’.^77^

**Paper:** folded in 4°, chain distance 32 mm, watermarks very similar to Mošin 647, 727, 733, 748 and 801, attested respectively in 1495, 1528, 1530, 1535–45 and 1560–5.^78^

**Binding:** of blind-tooled brown leather over cardboard; four ridges on the spine. – Pastedowns and flyleaves: replaced, chaindistance ca. 26 mm, partly preserved watermark very similar to Heawood 1743 (fleur-de-lis), attested in 1767. – F. 156: chain distance 23 mm, watermark similar to Heawood 302 (circles), attested in 1727–51. – Ff. 157–162: chain distance 28 mm, watermark *three crescents*.

**Marks of ownership:** (155r) ‘῎Εν ετοι ͵αεζβ΄ ἐν μηνη δεκεμβρίου βγ΄ ἡμέρα τρίτη· ἡς δόξαν Θ(εο)ῦ· ἐγενήθη ἡ δοῦλη τοῦ Θ(εο)ῦ· Κατακουζηνή· ὅρα ἐνάτοι, ἡς τὴν Θεσαλονίκι. ἔν ετος ͵αεηδ΄ μηνὶ ᾿Ιανουαρίου αβ΄ ἡμέρα Τρίτη ξημερόνοντας ἔδοκεν το κινῶ χρέος· η θειγατέρα μου η ἄνωθεν δούλ(η) τοῦ Θ(εο)ῦ Κατακουζηνή’ – (155r) ‘Ετος ͵αεηδ΄ μηνὶ αὐγούστου ε΄ εγενήθην ης δόξαν Θ(εο)ῦ ο δουλος τοῦ Θ(εο)ῦ ὀνόματη Γιακοῦμος’ – (155v) ‘Ετησιν παρον βεβλιον περι γιατρικις του Μαριανου’ – (155v) ‘Andreas Charagas’.

**Provenance:** London Medical Society (acquired most probably between 1803 and 1829); purchased by the Wellcome Library in 1984.

Bibliography: *Cat. Med. Soc*. 1829, 201; Nias, lvi; Dawson, 130–1; Touwaide, 539.

## 14. MS.289

Italy, ca. 1535 AD (from watermarks).

Paper, 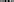, 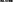 (foliated 1–30), linn. 25 [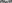], 02D1.

Ps.-Galen, *Medical Definitions* (TLG 0530.041).^79^

**Text: [1r–30r]** **Γαληνοῦ ὅροι ἰατρικοί**, inc. Τὴν τῶν ὅρων πραγματεῖαν, πολυωφελεστάτην ὑπάρχουσαν πᾶσι τοῖς ἰατροῖς, des. ἐνθουσιασμὸς (δὲ) ἐστι, καθάπερ ἐξιστανταί τινες ἐπὶ τῶν ὑποθυμιωμένων ἐν τοῖς ἱεροῖς ὀρῶντες, ἢ τυμπάνων, ἢ αὐλῶν, ἢ συμβόλων ἀκούσαντες. τῷ Θεῷ δόξα 
Kühn, XIX.346–349.5, 349.9–11, 349.18–351.7, 352.5–364.15, 365.8–376.4, 378.4–416.6, 419.12–4, 420.11–421.3, 419.8–11, 421.4–12, 423.4–6, 423.14–7, 416.7–419.7, 419.15–420.10, 423.7–13, 423.18–424.6, 421.13–423.3, 424.7–428.8, 351.8–352.4, 428.9–462.^80^

**Blank page:** 30v.

**Annotations** (scribal)**:** (10r, 11v, 12v, 16v, 17v, 20r, 26r, 28v, 29v) additions to the main texts or variant readings, eg. (10r) ‘φρόνησις’, (29v) ‘Γρ(άφεται) κειμένου’.

**Handwriting:** unidentified Renaissance hand.

**Paper:** folded in 4°; chain distance 35 mm; watermark similar to Sosower *balance* 2, attested in 1534.^81^

**Quire signatures** (scribal)**:** Greek numerals *Im3* (β΄ on f. 8v) and *Im1* (γ΄ on f. 17r, δ΄ on f. 25r). – ic xc written, in the scribe’s hand, in the middle of each page’s upper margin.

**Quires:** 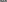 (f. 24), 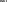 (f. 26), 4 (f. 30) [no text lost].

**Binding:** of cardboard, modern. – Flyleaves and pastedowns: machine-made paper.

**Marks of ownership:** (1r) ‘F. L. 2711’ – (1r) ‘43753A’ – (4r) erased and illegible note.

**Provenance:** [R. Lier & Co, Milan]; purchased on the behalf of Wellcome Library in 1925 (accession number 43753A).

**Bibliography:** Moorat, I.189; Touwaide, 539.

## 15. MS.354

Paper, 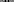, 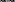 (foliated 1–108).

**[1r–18v]** Damascius, *Commentary on the Aphorisms of Hippocrates* (TLG 0728.001).^82^ **[18v–21v]** [Hippocrates], *Prognosticon* (TLG 0627.003).^83^ **[22r–107v]** Stephen,*Commentary on the Prognosticon of Hippocrates* (TLG 0728.001).^84^

**Note:** This MS consists of two distinct parts, described below separately as **A** and **B**.

**A.**

Byzantine Empire, ca. 1400–30 AD (from watermarks).

Linn. 40 [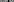], D 32D; (scribe B) linn. 41–45, unruled.

**Text: [1r–18v]** **῾Αφορισμῶν ῾Ιπποκράτους· τμήμα πρῶτον**, inc. ῾Ο βίος βραχύς· ἡ δὲ τέχνη μακρή· ὁ δὲ καιρὸς ὁξύς· ἡ δὲ πεῖρα σφαλερή· ἡ δὲ κρίσις χαλεπή· δεῖ δὲ οὐ μόνον ἑωυτὸν παρέχειν τὰ δέοντα ποιέοντα, ἀλλὰ καὶ τὸν νοσέοντα· καὶ τοὺς παρέοντας· καὶ τὰ ἔξωθεν: ἑρμ(η)ν(εία)· ἤ(γουν) εἰ βούλετ(αι) ὁ ἰατρὸς· (καὶ) τὸν ἀσθενοῦντα θεραπεῦσαι, des. ἐπὶ λευκῷ φλέγματι, ὕδρωψ ἐπιγίνετ(αι): τέλος τῶν ῾Ιπποκράτους τοῦ Κώου ἀφορισμῶν 
Littré, IV.458.1–4; Jones 98.1–5; Dietz, II.250.22–251.21; *id.,* IV.458.5–10; 98.6–13, II.256.8–258.2; *id.,* IV.458.11–460.6; 98.14–100.8, II.260.3–261.8; and so forth up to *id.,* IV.596.8–20; 208.4–7, II.543.14–6; then *id.,* IV.596.9–598.3; 208.8–14; IV.598.5–604.4; 210.1–214.4.**[18v–21v]** **Προγνωστικ(ὸν) τοῦ αὐτοῦ**, inc. Τὸν ἰητρὸν δοκέει μοι ἄριστον εἶναι, πρόνοιαν ἐπιτηδεύειν, des. πάντα γὰρ ὁοκόσα ἐν τοῖσι χρονίοισι, τοῖσι προειρημένοισι κρίνετ(αι)· γνώση δέ, τοῖσιν αὐτέοισι σημείοισιν: γνώση δέ, τοῖσιν αὐτέοισι σημείοισιν: τέλος τοῦ προγνωστικοῦ 
Littré, II.110–190; Alexanderson, 193–231; Jouanna, 1–80.^85^

**Annotation** (scribal)**:** (18r) ‘δι΄ ἐμέτων’ added in the margin.

**Handwriting:** two scribes A (1r–18v *supra*) and B (18v *infra*–21v).

**Paper:** folded in 2°; chain distance 35 mm; watermark very similar to Piccard 150435, 150483, 150564, 150755 (three hills), attested in 1408–27.

**Quires:** 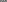 (f. 16), 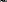 (f. 21) [no text lost].

**B.**

Venice, Germany or Spain, ca. 1582–7 AD (from watermarks).

Linn. 20 [ca. 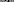], unclear ruling.

**[22r–107v]** **Δαμασκίου φιλοσόφου ἐξήγησις εἰς τὸ προγνωστικὸν τοῦ ῾Ιπποκράτους: τμῆμα α** ^**ον**^, inc. Τὰ προλεγόμενα ἤως εἰωθότα ἐπὶ ἑκάστου συγγράμματος, des. τὸν ὕδερον ἐπὶ ψύξει κατὰ πρῶτον λόγον γίνεσθαι 
Duffy, 26–146.2.^86^

**Annotations** (scribal)**:** (64v) ‘γρ(άφεται) φαίνονται’ – (83v) ‘γρ(άφεται) γεννᾶσθαι’ – (85r) ‘γρ(άφεται) ψυχήν’ – (105v) ‘περὶ ἡπατηρᾶς δυσεντερίας’ – (106r) addition to the text: ‘μεταξύ’ – (107v) ‘ἐλλιπὲς ἦν τὸ τέλος ὑπὸ τῆς ἀρχαιότητος’.

**Handwriting:** attributed by Otto Kresten to Andreas Darmarios (RGK I 13, II 21, III 22).

**Paper:** folded in 2°; chain distance 32 mm; watermark identical with Sosower *croix latine* 45–46.

**Catchwords** (scribal)**:** horizontal, *Ii3*.

**Quires:** 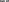 (f. 105), 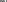 (f. 107).

**A and B.**

**Binding:** of white parchment over cardboard; flyleaves and pastedowns conjoint, unidentified watermark with escutcheon and the motto ‘J ROIG FA DIA SERRA’ [?], countermark ‘F - S - MERCADE’ [?].

**Marks of ownership:** (front pastedown) ‘24902’, ‘59. F. 19 / 1246’, ‘Rosenthal 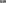
’ – (front flyaleaf I) ‘Hippocrates Aphorismi / Lez. XXIII 440335 / M ONJ’, ‘Aphorismi Hipocratis’.

**Provenance:** purchased by the Wellcome library in 1910 (accession number 24902).

Bibliography: Moorat, I.225–6; Duffy, *op. cit*. (note 84), 13, 18, 68–70; Touwaide, 539; Jouanna, *op. cit*. (note 48), lxxxv, xci.
Caroline Magdelaine, ‘Le commentaire de Damascius aux Aphorismes d’Hippocrate’, in Antonio Garzya and Jacques Jouanna (eds), *Storia e ecdotica dei testi medici greci: Atti del II Convegno Internazionale, Parigi 24–26 maggio 1994* (Napoli: D’Auria, 1996), 289–306: 291, 293.
Sibylle Ihm, *Clavis commentariorum der antiken medizinischen Texte* (Leiden: Brill, 2001), 79–80, 203–4, 290.
Mark Sosower, *Signa officinarum chartariarum in codicibus Graecis saeculo sexto decimo fabricatis in bibliothecis Hispaniae* (Amsterdam: Haakert, 2004), 36, 276–7, 488.

## 16. MS.413

Ottoman Empire, ca. 1800 (from watermarks).

Paper, 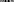 mm; 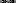 (foliated 1–14), linn. var., unruled.

**[1r–8v]** Ps.-Leo VI the Wise, *Oracles*. **[10r–v]** Arsenios Markellos, *Oracles*. **[9r–v, 11r–13v]** Anonymous collection of oracles.

**Text: [1r]** **᾿Αντίγραφον**  **Λέοντος τοῦ σοφοτάτου βασιλέως τῆς Κωνσταντινουπόλεως**, inc. Βυζάντιος αὐλή ἐστί ἡ Κωνσταντίνου ῾Ρώμην Βαβηλών, des. τοῖς ἴχνεσι σου προσπεσόντων τῶν πάλαι 
Lambeck, 1149–50. **[1v]** **Μετάνιας**, inc. Αἰτοῦ (sic.) τὸ τρίτον καὶ γὰρ ὄρνις, des. ἀρχὴν ἔχων τε τὴν μονάδα κ(αὶ) τέλος 
Lambeck, 1129.29–1132.10; Brokkaar *et al.*, 60.1–3.7–8. 10–3.4–5. **[2r]** no title, inc. Καὶ θαρσὺς ὡς μάλιστα καὶ παχὴς πέλεις, des. κ(αὶ) χρηματίζεις ἡνίων φλιᾶς ἄπερ 
Lambeck, 1132.12– 8; Brokkaar *et al.*, 62.2–3, 6–8, 4–5. **[2v]** **῎Επαρσις**, inc. ῞Ορα δὲ πάλιν ξένον δηθὲν τρόπον, des. διέστησαν δὴ τὰς πάλας συνεγραμμένας 
Lambeck, 1132.19–1133.2; Brokkaar *et al.* 68.1–3.6–10.4–5. **[2v]** Καὶ δ᾿ ἐπιτηρεῖν τῶν ὀκτὼ μερῶν μῆνες τουτέστιν εἱς ιδ΄ ἡμέρας τῆς σελήνης· καὶ ἐξ ἐκείνης ἀριθμή ἡμέρας ρπ΄ καὶ εὑρήσεις τὴν ἡμέραν τοῦ πότε μέλλει γενέσθαι τὸ Πάσχα τὸ μέγα. **[3r]** no title, inc. Οὗτος πέλων τέταρτος ἐξ ἄρτο τρέχων, des. ἡ χεὶρ καὶ δρέπανον αὐτὸ γε 
Lambeck, 1133.4–15; Brokkaar *et al.*, 70.2–3.7–13.4–6. **[3v]** no title, inc. Η θοὺς (sic) δὲ πέμπων, καὶ τέλος ταρκτοτρόφον, des. πρῶτας γὰς ἰσχεῖς ἀρετῶν ἄλλων πλέον 
Lambeck, 1133.17–25; Brokkaar *et al.*, 64.2–3.6–9.4–5. **[4r]** **Μελησμός**, inc. ῎Αλλη τις ἄρκτος δευτέρα σεμνοτρόφος, des. εἰς ἐσχάτων γὰρ γράφεται τῆς ἐσχάτης 
Lambeck, 1133.26–1136.3; Brokkaar *et al.*, 66.1–3.6–7.4–5. **[4v]** **Αἰμα**, inc. Αί αι τάλαινα πολυπαθεστάτην πόλις, des. δράκοντα συῤῥίζουσι τρυλοκτόνον 
Lambeck, 1136.4–17; Brokkaar *et al.*, 72.1–5.10–5.6–8. **[5r]** **Εὐχαρηστία**, inc. ᾿Αλώπεκην δὲ ὑπεκρίθη φιλίαν, des. ἐν τῷ τέλει δὲ ἤλειφας βραβεῖον συνήπτρον 
Lambeck, 1136.19–27; Brokkaar *et al.*, 74.1–10. **[5v]** no title, inc. Οὐαί σοι πόλις ἑπτάλοφος ὅταν τὸ εἰκοστὸν, des καὶ ἐν τῷ ὑψίστῳ βλασφημήσει 
Lambeck, 1136.30–6; Brokkaar *et al.*, 76.2–8. **[6r]** no title, inc. ᾿Ισαάκιος συγκοπῆ φόνου αἱμάτων, des. καὶ ἀποκαληφθήσεται οἰλημένος ἐπώνυμος Μεναχὴμ τουτέστι παραμυθία 
Lambeck, 1136.38–1137.7; Brokkaar *et al.*, 78.2–10. **[6v]** no title, inc. Τὴν πέτραν οἰκῶν ἄγε δεύρο μοι ξένε, des. γυμνὸς πάλιν εὔδευσον εἰς γῆς πυθμένα 
Lambeck, 1137.9–15; Brokkaar *et al.*, 80.2–8. **[7r]** **Εὐσέβεια**, inc. ῾Ο νεκρὸς ἤδη καὶ θέα λελημένος, des. ἄξατε τοῦτον εἰς βασιλείους δόμους 
Lambeck, 1137.16–25; Brokkaar *et al.*, 82.1–5, 11–3, 7–10. **[7v]** **Προτήρησις**, inc. ᾿Ιδοὺ πάλιν ἄνθρωπος ἐκ πρώτου γένους, des. διπλουμένων ὕπησει νεκρῷ τὴν πέτραν 
Lambeck, 1140.1–8; Brokkaar *et al.*, 84.1–10. **[8r]** **Προχείρισις**, inc. Δέξαι τὸ δῶρον μὴ κάτω κνείμη γέρων, des. ἐν σοὶ γὰρ ἀρχῆ τῶν ἀγαθῶν καὶ τέλος 
Lambeck, 1140.9–24; Brokkaar *et al.*, 86.1–16. **[8v]** no title, inc. Καλοῦ βίου τέτηχας ἐξ ἀδωξίας, des οὐκ αστοχήσεις τῆς ἄνω κληρουχίας 
Lambeck, 1140.26–30; Brokkaar *et al.*, 88.2–6. **[9r]** **Εἰς τὸ εὔδομον καὶ πέμπτον ᾿Ισμαὴλ τὸ τέλος ἀποκαληφθήσεται**, inc. Πέντε δένδρη βασιλεύον τὴν ἑπτάλοφον τὴν πόλιν, des. καὶ τὴν ρίζα καὶ τὰ φύλα καὶ νὰ ξερανθὴ τελείως 
Bouboulidis, 212. **[9r–v]** no title, inc. ῎Ιδα λύκον ἀγριομένον μέγα καὶ παχύν ὡς χοίρον, des. καὶ φάγε τὸν μυαλόν του  *id.*, 213. **[10r]** **Κύρου ᾿Αρσενίου τοῦ Λα** ^**λ**^ **καὶ προτονότου πατριάρχου**, inc. Μονοκέφαλον θηρίον ἐφανίσθη ἐν τῷ κόσμῳ des. καὶ εἰς βόσκημα ἐμπήκαν 
Bouboulidis, 213; Lampros, 113–4; Kyriakou, 187. **[10v]** no title, inc. Εἰς ἐνέα πέντε καὶξη, des. ἐκ τὴν ἔπαρσιν τὴν ἔχει 
Bouboulidis, 214; Lampros, 114; Kyriakou, 187–8. **[11r]** no title, inc. Βασιλεύοντος τοῦ ζ΄ εἰς τὴν πόλιν Βυζαντίδα, des. καὶ νὰ τὸν διαμοιραστῶσι 
Bouboulidis, 213. **[11v]** no title, inc. ᾿Εκ τὰ μέρη τῆς ἑσπέρας μαύρες στρογγηλες, des. ἐκ τὰ ἄλλα μέρη καὶφαγαν τὴν κεφαλήν  *id.*, 214. **[11v]** no title, inc. Τοῦ τετάρτοου γὰρ τὰ ἐξ δήδομας, des. γένη πρῶτος  *id.*, 214. **[12r]** no title, inc. Τούτον ὁ νοῶν νόητο, des. καὶ τὰ μάτά του εὐγάλαν  *id.*, 215. **[12r–v]** no title, inc. Μέσα περιβόλι ἐμπῆκα, des. ἔχασε τὴν βασιλείαν τοῦ μημηκρὴ ἡ ἀλωποῦς τα τὴν πάρει  *id.*, 215. **[13r]** no title, inc. ᾿Αναμέσον πέντε ἄστρων, des. καὶ οἱ ἄλλοι ἐμαλώναν  *id.*, 216. **[13v]** no title, inc. ᾿Από κάτω τοῦ πλατάνου, des. εἰς τὰ ὕψη τῶν ὀρέων  *id.*, 216. **[14r]** **ΤΕΛΟΣ**. inc. Τούτου ὁ νοῶν νός το μόνον, des. τὸ κακὸν θηρίον στοὺς δαλμάτας (same text as on f. 12r, ll.1–5).^87^

**Handwriting:** unidentified late post-Byzantine hand.

**Illustrations:** (1v–11v, 12v–13v) twenty-two figural drawings in ink and wash.

**Text added** by a later hand**:** (14v) Inc. Εἰς τὸ θέατρον τοῦ κόσμου με τα μ[[- -]]ετρα τοῦ νοὸς μου σαν τὸν χορισμὸν πληγῆν, des. πὸς τοῦ χορισμοῦ ἡ ζάλη μέφερε σε τέτιο χάλη δὲν εἶναι ἕνας να γρικά / ἰατρὸς δεν χρησιμέβη, γιατρεὶς δεν θεραπεβη ἀλὰ οὐδε ὁφελή  
Karatzas and Psalidas, 102.^88^

**Annotations** (non-scribal)**:** (1r) ‘Λέοντος τοῦ σοφοτάτου βασιλέως τῆς  Κωνσταντινουπόλεως ’. – Labels for some of the pictures: (3r) ‘τὸ τραπανι της εξκουσιυς του βασιλέος’, (3v) ‘λαφος’, (4r) ‘λικος’, (6r) ‘λάφι’, (9r) ‘θιριον ης τον δεντρο’, (10r) ‘μονοκεφαλος θιρ’ – (front flyleaf I) ‘Αἱ παραδόσεις τῶν κειμένων αὐτῶν δὲν ἐξηρευνήθησαν ἔτι ἱκανῶς. Χρησμοὶ Λέοντος τοῦ Σοφοῦ μετὰ 22 εἰκόνων ἐγχρώμων’.

**Snippets of text** on reused paper**:** (front flyleaf I) inc. Θεὸς μαιτάνοιαν νὰ γνοροίζουσοι  [γ]νοροίσουσοι τοίν ἀλοίθοιαν καὶ νά εξοιπνοίσοσοιν ἀπό τοιν παγοί[δ]α τού διαβόλου, des. καὶ ἐδόκε ἐκοί οἰς τοίν βασοιλοίαν τού Θεού διά τό σεσοσμαίνον  σεσοσμέ[νο]ν σας – (back flyleaf I) inc. (first complete line) οἰς τό τροίτον [ευρι]ισκομαι τό τροίτον τοίς ἀγοίος τριάδ[ος] πρόσοπον τό ὀποίον οἴναι θαιός τό πνευ[μα] τό ἄγοιον καὶ τόν ἀγοιασμόν ημόν, des. ὀ θαιός ἐφαναίροσας τόν ἐαυτόν.

**Paper:** folded in 4°; chain distance 20 mm; watermark generally similar to Eineder 315 (coat of arms with lion), attested in 1804; countermark with the letters FL under a decorated arch.^89^

**Quires:** 12 (ff. 1–11, 14)  2 (ff. 12–13).

**Binding:** of marbled paper over cardboard; cloth pastedowns; the pastedowns of an older binding have been retained as front and back flyleaves.

**Marks of ownership:** (front pastedown) label inscribed ‘LH 8’ – (1r) circular stamp ‘george p. beglery constantinople’ with ‘᾿Αριθ. 79’ handwritten in the centre.

**Provenance:** Georgios P. Vegleris (1850–1923), Constantinople (his no. 79) – Lionel Hauser (1868–1958), Paris (his no. 8); [his sale, Sotheby’s, London, 17 April 1934, lot 331]; purchased on the behalf of Wellcome Library (accession number 66627).

Bibliography: Moorat, I.280–1.
*Catalogue of  the Very Extensive and  Important Library of  Early Books and  Manuscripts*: *Relating to Alchemy & the Occult and Physical Sciences, the Property of M. Lionel Hauser* (London: Sotheby & Co, 1934), 36–7.
Lydie Hadermann-Misguich and Jeannine Vereecken, *Les oracles de Léon le Sage illustrés par Georges Klontzas: la version Barozzi dans le Codex Bute* (Venice: Institut Hellénique de Venise, 2000), 51.

## 17. MS.498 (*olim* Nikolsburgensis II.241)

Probably Constantinople,^90^ 1492 AD (from paschal tables on ff. 66v–67r).

Paper, 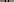, 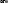 (foliated 1–86).^91^

**[23r–24r]** Verses by Manuel Korinthios. **[25r–27v]** Anonymus, *Epitome III* of Hephaestion of Thebes’ *Apotelesmatika* or *Astrological Effects* (TLG 2043.002). **[31r]** Ps.-George Chrysokokkes, *List of Equivalent Ancient and Modern Toponyms*. **[31v]** Anonymous verses. **[32r–68r]** Michael Chrysokokkes, *Hexapterygon*. **[68v]** Michael Psellos, *Concise Answers to Various Questions*, excerpt (TLG 2702.028).

**Ruling** (dry-point)**:** (ff. 1–22, 28–30, 70–86) none; (f. 24) rectangular frame with squares inside [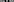]; (ff. 25–27) linn. 25–27, ca. [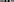], unclear ruling; (ff. 31–41) rectangular frames with lines inside, linn. 30–31 [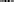]; (ff. 42–69) ruling for tables, size varies from [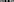] to [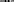].

**Text: [23r]** Οἶκος πέφυκας τῆς ὅλης θεαρχίας / ῥόδον τεκοῦσα μυστικῆς εὐωδίας· / ἡ γὰρ ἑπισκίασις ὑψίστου κόρη τῆς φύς  / τῆς φύσεως ἐξῆρε μειρόπων ἄνω· / ὦ παντάνασσα τοίνυν εὐλογημένη / ῥύου με δεινῶν κ(αὶ) λύπης σὸν οἰκέτην: – μεγαλόδωρε χαῖρε χαρμάτ(ων) πίδαξ / ἄνασσα κόσμου ὑπὲρεὐλογημένη· / νέμοις χαριτόβρυτον ὕδωρ μοι λόγου / ὄφρα λιγαίνω ἐν χαρᾷ τὴν σὴν χάριν· / ὕπὲρ λόγον γὰρ σὺ τεκοῦσα τὸν λόγον, / ἥγγισας ἁγνὴ τὴν βροτῶν φύτλην ξένως· / λαμπρὸν χαρίτων χαῖρε ταμεῖον κόρη. – ῾Ο λαμπρὸς αἰγλήες τε κυρίου θρόνος· / ῥάβδος βασιλείας τὲ τῆς οὐρανίου· / ἡ δεξιὰ χεὶρ τοῦ Θ(εο)ῦ Παναγία / τὴν μικρὰν αἴτησίν μου εὖ δεξαμένη· / ὡς ἀγαθὴ πλήρωσον ἐν τάχει κόρη / ῥοὴν γὰρ οἶδας τῶν ψυχικ(ῶν) δακρύ(ων).^92^ **[24r]** **Στίχοι ἰαμβικοὶ εἰς τ(ὴν) κυρί(αν) ἡμῶν Θεοτόκ(ον) τριχῶς ἀκροστιχιζόμενοι**, inc. Μεγαλύνω σε θεῖε ναέ κυρίου, des. λαμπρῶς βοῶσα εὐμενοῦς χαῖρεθρονε 
Stephanidis, 470; Hörandner, 42; cf. Vassis 449. **[24r]** no title, inc. Ματαιοτήτων ἅπαντα τυγχανει ματαιότης, des. συνάξωμ(εν) τοίνυν τ(ὸν) νοῦν πρὸς μόνον τ(ὸν) δεσπότ(ην) 
Treu, 539. **[24v]** ῾Ο κυριεύων τῶν ὅλων παντοκράτωρ / ῥώμῃ κραταιᾷ κ(αὶ) φύσει ἀκαμάτῳ, / ἠμπέσχετο βρότειον ἀρρήτως φύτλην / τὸ βασὶλειον μὲν κράτος φυᾶ ἔχων· / ὡς ἱερεὺς δὲ τὸν ποδήρη ἐκ νόμου· / ῥευστὴ βοάτω κυρίῳ δόξα φύσις.^93^ **[24v]** Μέγιστον ὄντως θαῦμα θείων ἀγγέλων· / ἀνεκλάλητον καὶ βροτῶν γλώσσαις ὅλων· / νύμφη ἄνυμφε μῆτερ ἀγνῆ τοῦ λόγου· / ὅς γὰρ τὸ πλάτος ἡψίδωσε τοῦ πόλου· / ὑπέσχε καὶ γῆς τὸν βρυθισμὸν ἀσχέτως· / ἡλίου ἀπήστραψε τ᾿ ἐν κόσμῳ φάος· / λαμπράν δ᾿ ἀνέσχε τῆς σελήνης ἀκτίνα, / οὗτος σοι εἰν ᾤκησεν εἰς σωτηρίαν· / ῥοώδεος φύσιος ἀνθρώπων κόρη· / ἥνπερ σέσωκε καὶ ἐδόξασε ξένως· / τῷ τοι χάριν σοι ἐκβοῶμεν εἰδότες· / ὦ χαῖρ᾿ ἀΰλων οὐσιῶν ὑπερτέρα· / ῥεῖθρον τε χαῖρε πρόξενον θείου βίου.^94^ **[25r–27v]** **῾Ηφαιστίωνος Θηβαίου ἀποτελέσματα συνοπτικὰ τῶν ἐκλείψεων τῶν φωστήρων ἐν μόνων τῶν ζωδίων**, inc. Σεληνιακῆς ἐκλείψεως γινομένης ἐν τῷ (Κριῷ) αφορί(αν) πάντων ἔσεσθαι, des. ἵνα δὲ μὴ μακρὸν τὸν ὑπομνηματισμ(ὸν) ποιήσωμ(εν), ἀφείσθω ταῦτα τοῖς εὐεπηβόλοις ἀφ᾿ ἐαυτ(ῶν) ἐπιγνῶναι 
Pingree, II.126–34. **[31r]** **῞Οσας τ(ῶν) πόλεων μετωνομάσθησαν ὕστερον**, inc. ᾿Επίδαμνος, τὸ νῦν Δηρράχιον, des. Μέμφη πόλις ἐν Αἰγύπτῳ, ἡ νῦν Ταμιάφιν· Πέργη τὸ νῦν Περγίν· Αὕδειρα, τὸ νῦν Πολύστειλον  
Lampsides, 320–2. **[31v]** **Εἱς τρισαιγλήεις εὑρυμέδων Θ(εό)ς**. ῎Αναξ γόνε παμφαής / αὐτοκρατόρων τῆς γῆς / ὧν κράτος δεύτερον ἦν / Θ(εο)ῦ τῶν ὅλων ἀρχῆς· / δέδεξο νῦν μερισμ(όν), / τῶν ζωδί(ων) μερικόν. – Τῶν ζωδί(ων) οὐρανοῦ, / τὰ μ(ὲν) ἄρσενα ἐστί / τὰ δὲ θήλεα φασί, / καὶ ἃ ἰσημερινά, / ἃ δὲ πάλιν τροπικά / κ(αὶ) τὰ μέν γε στερεά, / δίσωμα δὲ τὰ λοιπά· / εἰσὶν οὖν ἀρσενικά· / ὁ Κριὸς οἱ Δίδυμοι, / Λέων ὁμοῦ κ(αὶ) Ζυγός· / Τοξότης ἐπισπερχής / ῾ϒδροχόος τὲ εὐθύς· / ἕξ τοίνυν ἀρσενικά, / τὰ λοιπὰ δὲ θηλυκά· / Ταῦρος ἰσχυρογενής / κ(αὶ) Καρκῖνος δυσκλεής· / ἡ Παρθένος ἡ αἰδώς / κ(αὶ) Σκορπῖος ὁ λυγρός· / ὁ Αἰγόκερως ὁμοῦ / κ(αὶ) ᾿Ιχθύες οἱ ψυχροί· / ἰσημερινὰ δ᾿ εἰσίν / ὁ Κριὸς κ(αὶ) ὁ Ζυγός· / ὁ Καρκῖνος δὲ ἐστί / τροπικός γε θερινός· / καὶ Αἰγόκερως ἐστί / τροπικὸς χειμερινός· / ἀλλὰ δὴ κ(αὶ) στερεά, / Ταῦρος καὶ Λέων εἰσίν· / κ(αὶ) Σκορπῖος ὁ λυγρός / ῾ϒδροχόος θ᾿ ὁ ὑγρός· / τὰ τέτταρα δὴ ταυτὶ / στερεὰ σοφοὶ φασί· / δίσωμα δὲ Δίδυμοι, / κ(αὶ) Παρθένος ἡ κεδνή· / ὁ Τοξότης ὁ οξύς, / κ(αὶ) ᾿Ιχθύες οἱ ψυχροί· / οὕτως ἔχει ὡς εἰπεῖν, / τῶν ζωδί(ων) ἡ σκηνή· / ἣν ζωδιακὸν φαμ(έν)· / κύκλον τρέχοντα αἰέν. **[32r–41r]** **Μιχαὴλ Νοταρίου τῆς μεγάλ(ης) ἐκκλησίας τοῦ Χρυσοκόκκη. ἔκδοσις γεγονυῖα εἰς τὸ ᾿Ιουδαϊκ(ὸν) ἑξαπτέρυγον, κατὰ τὸ ͵ςϡμγ΄ ἔτος ἀπὸ τῆς ἀρχῆς τοῦ παντός**, inc. Τῆς τῶν πλανωμέν(ων) ἀστέρων φορᾶς, ποικίλλης γε οὕσης κ(αὶ) πολυειδοῦς, des. οὕτω γὰρ ὑγιαίνει πάντα τὰ μεταξὺ εἰ δὲ μὴ ἐξισάζοι, ἔπται σταίπη, κ(αὶ) δεῖ διορθώσ(ει)ς, λογίζονται δὲ τῇ ὥρᾳ, στιγμαὶ ͵απ΄ · ἤτοι τῶν ἑνὶ λε(πτ)ῷ, στιγμαὶ ιη΄ 
Solon, 15–61, 117.45–133.365. **[42r–66r]** **Πτερὸν πρῶτον**, inc. ᾿Εννεακαιδεκατηρίδων κίνησις συνοδικ(ῶν), etc. 
Solon, 285–93, 299–330, 336–7, 345, 348, 350–3, 355, 357–71, 375–6; colophon: Τέλος σὺν Θ(ε)ῷ ἁγίῳ τῷ κ(υρί)ῳ ἡμ(ῶν) ᾿Ι(ησο)ῦ Χ(ριστ)ῷ. **[66v–67r]** Index of the solar and lunar cycles and the moveable feast dates for the years ͵ζα΄ through ͵ζκη΄  
Solon, 381–2. **[67v]** Index of the dominant zodiacal sign for each day of the twelve months  
Solon, 383–4. **[68r]** **Κανό(νες) τοῦ μήκ(ους) καὶ πλάτ(ους) τῶν ἐπισήμων πόλεων: ἀπὸ τῆς Ταραγκιν(ῶν) πόλ(εως) ἀριθμούμ(εν)αι** 
Solon, 386. **[68v]** **Περὶ μήκ(ους) (ἡλίου) κ(αὶ) (σελήνης) κ(αὶ) γῆς**, inc. ῾Ο ἤλιος πρὸς τὴν γῆν κατὰ τὸν ἀστρονομικότατον ᾿Αρίσταρχον μείζονα λόγον ἔχει, des. ἀλλὰ μετὰ γεωμετρικῆς ἀποδείξεως κ(αὶ) ἀν ἀντιρρήτου περὶ ἧς οὐ καιρὸς νῦν λέγειν 
Westerink, section 127, 67–68).^95^

**Blank pages:** flyleaf I, ff. 1r–22v, 23v, 28r–30v, 41v, 69r–86v.

**Illustrations:** (59v–65r) ink drawings of the zodiacal figures.

**Handwriting:** A (ff. 24r *infra*, 24v *infra*, 31r, 32r–41r), B (ff. 23r, 24r *supra*, 24v *supra*, 25r–27v, 43r–68v). Rudolf Stefec identified scribe B with Manuel Korinthios.^96^

**Annotations** (non-scribal)**:** (flyleaf I) ‘Titulus libri mathematici’ – (flyleaf I) ‘Ηφαιστίωνος Θηβαίου ἀποτελέσματα συνοπτικά τῶν ἐκλείψεων τῶν φωστήρων ἐκ μόνων τῶν ζωδίων. Ebestionis Thebai opera consummata, compendiosa, de ecclipsibus luminarium coelestium, ex solis zodiaci circulis. (Secun)dus tractatus ῞Οσας τῶν πόλεων μετωνομάσθησαν ὕστερον. Cuiae Urbes diversimode nomina sortutae sint. In fine adiuncta sunt tabula Ecclipsium luminarium coelestium, iuxta doctrinam antecedentem.’ – (back of side flap) arithmetical calculations.

**Paper:** folded in 2°; chain distance 36 mm; watermarks very similar to Heawood 2467 (hand), attested in 1503, and to Piccard 155891 (hand), attested in 1522.^97^

**Quires:** 1 (flyleaf i), 4 (f. 4), 1 (f. 5, conjoint with front pastedown), 10 (f. 15), 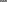 (f. 31), 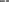 (f. 71), 8 (f. 79), 1 (f. 80, conjoint with pastedown), 6 (f. 86).

**Binding** original to the MS**:** Ottoman style, with a side flap; blind-tooled and gilt-tooled brown leather over cardboard. – Flyleaf i: chain distance 25 mm; no watermark. – Pastedowns: same paper as the rest of the MS.

**Marks of ownership:** (front pastedown) engraved ex-libris inscribed: ‘Ex Bibliotheca Illustris ac Generosi Domini D(omi)ni Ferdinandi Hoffman liberi baronis in Grunpühel et Strecau, D(omi)ni in Grevenstein et Ianowitz, supreme haereditarii curiae magistri ducatus Styriae et supremi marsalci archiducatus Austriae, sacratissmae: caesae: et regiae maiestatis consiliarii et camerae aulicae praefecti. etc.’^98^

**Provenance:** Ferdinand Hoffmann (1540–1607), Freiherr von Grünbühel and Strechau; by descent;^99^ inherited by his great-granddaughters Maria Elisabeth Hoffmann (1663–1705) and Johanna Maria Xaveria Hoffmann (d. 1706); presented to their guardian Ferdinand Joseph von Dietrichstein (1636–98) in 1679;^100^ by descent; inherited by Hugo von Mensdorff-Pouilly (1858–1920), Fürst von Dietrichstein zu Nickolsburg (his no. II.241); inherited by Alexander Albert Olivier Anton von Mensdorff-Pouilly-Dietrichstein (1899–1964), Nickolsburg/Mikulov; [his sale, H. Gilhofer and H. Ranschburg, Lucerne, 25–26 June 1934, lot 352]; purchased on the behalf of Wellcome Library (accession number 66711).

Bibliography: Moorat, I.329–30; Pingree, *op. cit*. (note 95), Vol. 2, xix.
Eduard Gollob, ‘Verzeichnis der griechischen Handshriften in Österreich ausserhalb Wiens’, *Sitzungsberichte der Kaiserlichen Akademie der Wissenschaften in Wien, Philosophisch-historische Classe*, 146 (1903), 1–173: 89–90.
*Bibliothek Fürst Dietrichstein*: (Luzern: Gilhofer & Ranschburg, 1933–4), II.42 (no. 352).
Peter Solon, ‘The *Six Wings* of Immanuel Bonfils and Michael Chrysokokkes’, *Centaurus*, 15(1970), 1–20: 17.

## 18. MS.4103

Northern Greece,^101^ 1697 AD (from the lunar tables on pp. 142–143).

Paper, 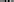, 125 leaves (partially pagined: 55–76, 81–86, 89–100, 105–116, 119–164, 187–196, 209–212, 271–280, 277[]–324, 329–342, 379–387, 378 [ie. 388], [409]–412; then foliated: 1–24),^102^ linn. 20–21 [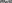]–[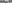], 00D1.

Anonymous collection of post-Byzantine *iatrosophia*.^103^

**Likeliest correct order of folia:** 1, 3, 5, 23, 6–8, 15–20, 22, 5 (reversed), 24 (reversed), 91–4, 21

**Text: [55–127]** no title, inc. Καὶ τοῦ πράσου να τον ψίσις, κ(αὶ) να τον στίψις, κ(αὶ) τον ανακατονις ὀμοῦ κ(αὶ) τὸν πάζις μέσα, εἰς τον αυτή οπου μαζη, κ(αὶ) πάβοι, des. γράψε οὕτος α· β· γ· δ· ε· ζ· η· θ· ι· κ· λ· μ· ν· ξ· ο· π· ρ· σ· τ· υ· φ· χ· ψ· ω· ους κ(αὶ) ας πίναι του παιδή, κ(αὶ) μαθένι ογλίγορα. **[128–129]** **Περι τω δωδηκαμινῶν, ταις ημέρις ταις τις εναντίαις κακές, κ(αὶ) καλές, ταις εφανέρωσεν ὁ Θεῶς, του προφίτου εις δράνα ταις ομουλογούν του κόσμου**, inc. Εαν γενιθή πεδή· δεν προυκάβι, εις ατυχη ημέρα, ἄναγοράσι, des. τέλος ὄλ[αις] εναντιαις ημέρις, κ(αὶ) κακες: τον δόδεκαμινον. **[130–136]** **῎Ενθίμισι πρὸς πάντας, ἄν(θρωπ)ος του τι τους καμι χρία, τον καθε μήναν, δια την υγίαν τους, τον μὲν πρότα ἀρχομεστε απου το μαρτιων μίναν: μιν Μάρτιον**, inc. Των μίναν, τω μάρτιων, χρύι καθε ἄνθρουπους, να τόγι πραγματα γλικά, des. κ(αὶ) νόσος πουλής, κ(αὶ) γερόντου θάνατος. **[136–159]** **Μέθοδως τοῖς σελίνοις, παρα τοις φλεβοτομίας τοις σελίνοις**, inc. Εις την –ι– εἴναι κακῶν, οτι την ομορφάδα, φέρνι εις κητερνάδα του σόματος, des. να του βάλοι εις του κάθισμά του αρόστου, κ(αὶ) κοιμάτοι. **[160–378]** **Γαληνοῦ διαθίκοι αρμόζοιτ(ων)**, inc. Τ(ων) ἰατρῶν, εις ασθενία, του ανθρόπου, εκ του ταισάρων στιχοίων, των δόδικαμοινῶν, πος γίνανται ι ασθένια εις του ἄνθροπου, des. της μελανοῖς χολίς τα σιμάδια, είναι αυτά, ξοιρῶς βοίχας, πόνως εις τον αριστη. **[7r–9r]** Inc. […]ουμενοι καλός, τοις πλιροὶ το ἔργον, εντροποικοις αραβῶνα δοῦνε, καλῶν ναυτες, κ(αὶ) εμπόροις, κ(αὶ) ὄσι περοι τα ζῶα πραγματέβωνται, des. τελιώνον, τα δώδεκα ζόδια εως του τελους τοις σελίνοις. **[3r–6r]** **Περοι ὦταν ευγένοι του κάθισμα του ανθρωπου**, inc. Βάλε κρῶκων τριμένον, κ(αὶ) ροδόσταγμον, κ(αὶ) κρόκον αυγοῦ, des. μετα αφεντοίας κυνοίσι, κ(αὶ) μι διαφορο. [6r–*116v] **᾿Ερμηνοιόν κύρου Μανουῆλ, τοῦ σωφοῦ περοι ὀτοις ιδεί ω ἄνθροπος ὁνοιρων, είς τας ιμέρας τοις σελοίνοις· τοι δηλοί πωνοιρ(ῶν) ει καλ(ῶν)**, inc. Εις –ι– ημέρα τ(ῆς) σελοίνοις. σαν οιδοίς ὠνοιρων, des. η δε σελοίνοις οὑσις εν το λέωντας, ἐὰν βρον[τοίσι]. [*117r] Inc. Ζοῦμοι  – 13 – βοῦτοιρων νωπῶν, des. μπεἇσωσαμ δράμια στ΄. [*118v–13r] Inc. Εὰν η πρῶτοι του σεπτεμβρίου μοινῶς, ειναι κυριακοί, γίνεται καρπῶς πολοίς, des. η δε νοικα τοις νήμφοις οὔ σιμφέροι κ(αὶ) λίποι. [*123v–21r] **Περι να κάμοις ἔλεων**, inc. ῎Ελεων, νάρδινων, του μέζουναι, ωφελεί, εις πάθι ψυχρά, κ(αὶ) εις τους ανέμους, του στομάχου, des. μέλιτος λήτρις – ι΄ – ποιησων περούλοις ος κεχρει[?].

**Annotations** (scribal)**:** (*passim*) occasional deletions/corrections to the text; (*31v, *46v) text in the outer margin – (pp. 280–281) additions to the main text.

**Illustrations** (scribal)**:** (pp. 142–143) lunar tables for the years from 1697 to 1715 – (p. 144, outer margin) drawings of a snake and a moon – (pp. 164, 290) apotropaic signs – (*102r–v, *107r, *118r) zodiacal tables – (*117v) table of the winds.

**Handwriting:** unidentified post-Byzantine hand.

**Annotations** (non-scribal)**:** (pp. 96, 98, 135, 138, 139) marginal notes – (*125v) poorly legible recipe.

**Old pagination** (possibly scribal)**:** Greek numerals *Se*, λγ΄ (p. 55) through νδ΄ (p. 76), νθ΄ (p. 81) through ξδ΄ (p. 86), ξζ΄ (p. 89) through οη΄ (p. 100), πγ΄ (p. 105) through ϟδ΄ (116).

**Paper:** folded in 4°; chain distance 28 mm; watermark very similar to Andreev 40.218 (attested in 1664 AD).^104^

**Quire signatures** (possibly scribal)**:** Arabic numerals *Ii1*, 3 (pp. 55, 57), 4 (p. 71), 7 (p. 119), 8 (p. 135), 9 (p. 151), 17 (p. 279[bis]), 18 (p. 293), 19 (p. 311)

**Quires:** 8 (p. 72), 6 (p. 86; innermost bifolium lost), 6 (p. 100; outermost bifolium lost), 6 (p. 116; outermost bifolium lost), 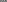 (p. 150), 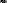 (p. 164; last leaf lost), 6 (p. 196; outermost bifolium lost), 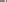 (pp. 209–212), 6 (p. 280; outermost bifolium lost), 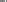 (p. 282[bis]), 6 (p. 292), 8 (p. 310), 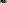 (p. 324; last leaf lost), 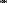 (p. 342; first leaf lost), 1, 2, 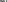 (p. 3[8]8), 1 (p. 410), 2 (p. 411–f. 1); ff. 3, 5, 23, 6, 22, 5, 24 and 21 are singletons; 2 (ff. 7–8); 6 (ff. 15–20), 6 (ff. 9–14).

**Binding:** none.

**Provenance:** [sale, Sotheby’s, London, 17 February 1936, lot 97]; purchased on the behalf of Wellcome Library (accession number 69261).

Bibliography: Moorat, II.877–8.
*Catalogue of valuable printed books, illuminated and other manuscripts, autograph*: *letters, topographical drawings of American interest, etc.: comprising, the library of Sir Algernon Methuen* (…) *on Monday, the 17th of February, 1936, and two following days at one o’clock precisely* (London: Sotheby & Co, 1936), 19.

## Footnotes

1. In addition to the Greek manuscripts, the library holds a few Greek papyri fragments (MSS 5753, 5754 and 5755); see, Vivian Nutton, ‘Wellcome papiro Oxyrhynchus 2547 e papiro Johnson’, *Kos*, 1, 6 (1984), 7–9, and Richard Palmer, *Catalogue of Western manuscripts in the Library of the Wellcome Institute for the History and Understanding of Medicine: western manuscripts 5120–6244* (London: The Wellcome Trust, 2000), 142–4. For a history of the Wellcome Library, see John Symons, *Wellcome Institute for the History of Medicine. A Short History* (London: The Wellcome Trust, 1993).

2. On the library of the Medical Society of London, see Penelope Hunting, *The Medical Society of London, 1773–2003* (London: Medical Society of London, 2003), 21–6, 291–305.

3. British Library, shelfmark 195a20: *British Library Database of Bookbindings* (http://www.bl.uk/catalogues/bookbindings, accessed 18 December 2014). Cf. Wellcome MS.MSL.62.

4. British Library, shelfmarks c46g7 and 269i10: *ibid.* Cf. Wellcome MS.MSL.52A and B. On John Brindley: George Smith, *The Oldest London Bookshop: A History of Two Hundred Years* (London: Ellis, 1928), 3–20.

5. Anthony Askew studied at Cambridge and Leiden, and was appointed physician to St Bartholomew’s Hospital in 1754. His surviving correspondence with Richard Mead and the German philologist Johann Jakob Reiske (1716–74) reflects his great love of Greek literature, including a project for a critical edition of Aeschylus’ works, which was never realised. On Askew and his library, see William Younger Fletcher, *English Book Collectors* (London: Kegan Paul, Trench, Trübner & Co., 1902), 219–21; and Seymour de Ricci, *English Collectors of Books & Manuscripts (1530–1930) and Their Marks of Ownership* (Cambridge: Cambridge University Press, 1930), 47, 52–3.

6. Eg. British Library, Add. MSS 5108 and 5119 (http://www.bl.uk/manuscripts, accessed 18 December 2014). The latter binding is identical to that of Wellcome MS.MSL.14.

7. *Bibliotheca Askeviana manu scripta, sive catalogus librorum manuscriptorum Antonii Askew* (London: Leigh & Sotheby, 1785), *passim*.

8. [William Macmichael], *The Gold-Headed Cane*, 4th edn (New York: Hoever, 1915), 151–2. Richard Mead studied at Utrecht, Leiden and Padua, and was elected physician to St Thomas’ Hospital in Southwark in 1703. He wrote several works on medical topics, including an influential monograph on the practice of inoculation for smallpox. He knew Greek and Latin, and his vast collection of books and manuscripts numbered some 10 000 volumes.

9. James Sims studied at Leiden and served as a physician to the General Dispensary in Aldersgate Street and to the Surrey Dispensary. He was President of the Medical Society of London for twenty-two years (1786–1808).

10. On Byzantine *iatrosophia*, see Anna Maria Ieraci Bio, ‘Testi medici di uso strumentale’, *Jahrbuch der Österreichischen Byzantinistik*, 32 (1982), 33–43. For an overview of post-Byzantine collections of recipes, see Agamemnon Tselikas, ‘Τὰ ἑλληνικὰ γιατροσόφια: μιὰ περιφρονημένη κατηγορία χειρογράφων’, in Thanasis Diamantopoulos (ed.), *᾿Ιατρικὰ Βυζαντινὰ Χειρόγραφα* (Athens: Δόμος, 1995), 57–69.

11. Joseph Sonderkamp, *Untersuchungen zur Überlieferung der Schriften des Theophanes Chrysobalantes sog. Theophanes Nonnos* (Bonn: Habelt, 1987). A critical edition of the text is currently being prepared by Barbara Zipser.

12. On *mathemataria*, see Angeliki Skarveli-Nikolopoulou, *Μαθηματάρια τῶν ῾Ελληνικῶν σχολείων κατὰ τὴν Τουρκοκρατία* (Athens: Σύλλογος πρὸς Διάδοσιν ᾿Ωφελίμων Βιβλίων, 1993).

13. Occasionally the text presents similarities with Porphyry’s commentary on Aristotle’s *Categories*; cf. Adolf Busse, *Porphyrii Isagoge et in Aristotelis Categorias commentarium* (Berlin: Reimer, 1887). [*Commentaria in Aristotelem Graeca*, Vol. 4.1], 55–142.

14. Asparukh Velkov, *Les filigranes dans les documents ottomans: divers types d’images* (Sofia: Trayanov, 2005); Edward Heawood, *Watermarks, Mainly of the 17th and 18th Centuries* (Hilversum: Paper Publications Society, 1950).

15. The first few phrases of the text coincide with the last paragraph (VI) of the treatise *On Human Birth* attributed to Alexander the Sophist; cf. Jacques Jouanna, ‘La postérité de l’embryologie d’Hippocrate dans deux traités pseudo-hippocratiques de la médecine tardive: “Sur la formation de l’homme et Sur la génération de l’homme et la semence”’, in Luc Brisson *et al.* (eds), *L’embryon: formation et animation* (Paris: Vrin, 2008), 15–41: 41.

16. The introduction coincides with fragment 93 attributed to Libanius; cf. Richard Foerster, *Libanii opera* (Lepzig: Teubner, 1922), Vol. 11, 3–5; cf. Diels II.57.

17. The text contains details on plebotomy and various simple drugs, recipes of composite drugs, and therapeutic recommendations on bites of venomous animals and poisons.

18. The text mainly contains remedies consisting of spells.

19. Cf. Diels II.37, III.50; and Stefan Weinstock, *Catalogus codicum astrologorum Graecorum* V. *Codicum Romanorum*, pt. 4 (Brussels: Regia Academia Belgica, 1940), 156–63.

20. The text contains details on various simple drugs and recipes of composite drugs in vernacular.

21. The following chapters, according to the numbering by Barbara Zipser, *John the Physician’s Therapeutics: A Medical Handbook in Vernacular Greek* (Leiden: Brill, 2009), are missing: 16–21, 55–66, 68–73, 134–7, 143–6, 242–53.

22. It contains texts on diagnosis of pulse and urines, and details on phlebotomy; there are also recipes of composite drugs and remedies with superstitious connotations including spells. Various excerpts have been edited by Barbara Zipser, ‘Magic, infidelity, and secret annotations in a Cypriot manuscripts of the early fourteenth century (Wellcome MSL.14)’, in Steven M. Oberhelman (ed.), *Dreams, Healing, and Medicine in Greece from Antiquity to the Present* (Farnham: Ashgate, 2013), 251–68: 257–8, 261.

23. Franz Zacharias Ermerins, *Anecdota medica Graeca e codicibus MSS. expromsit* (Leiden: S. et J. Luchtmans, 1840); Jouanna, *op. cit.* (note 15); Julius Ludwig Ideler, *Physici et medici Graeci minores*, 2 vols (Berlin: G. Reimer, 1841–42); Karl Sudhoff, ‘Die pseudohippokratische Krankheitsprognostik nach dem Auftreten von Hautausschlägen, “Secreta Hippocratis” oder “Capsula eburnea” benannt’, *Archiv für Geschichte der Medizin*, 9 (1915), 79–116; Zipser, *op. cit.* (note 21).

24. Vladimir Mošin and Seid Traljić, *Filigranes des XIIIe et XIVe siècles*, 2 vols (Zagreb: Académie yougoslave des sciences et des beaux-arts, 1957).

25. The text is lacunose and not clearly segmented. I have subdivided it in correspondence with Zipser’s partial edition.

26. Barbara Zipser, ‘Die uneinheitliche Überlieferung eines Fragmentes über den Puls und ein Iatrosophion’, *Galenos*, 2 (2008), 129–34.

27. Barbara Zipser, *op. cit.* (note 21), 16. Cf. Costas Constantinides and Robert Browning, *Dated Manuscripts from Cyprus to the Year 1570* (Washington DC: Dumbarton Oaks, 1993), pl. 44 (no. 30).

28. Edited by Zipser, see Nutton and Zipser, 264 (no. 21).

29. Some of them partly edited by Zipser, *op. cit.* (note 22), 264.

30. Mošin and Traljić, *op. cit.* (note 24).

31. Thomas L. Gravell and George Miller, *A Catalogue of Foreign Watermarks Found on Paper Used in America, 1700–1835* (New York: Garland, 1983).

32. Books 3–6 have no Greek edition yet and have only been published in Latin translation. The MS belongs to a family where the first part of Book 6 forms the second part of Book 3 and the second part of Book 6 comes after Book 5. The second part of Book 6 is missing in this MS.

33. For example, 340v ‘Τίς ὁ δορκαδίζων: λέγεται σφυγμὸς δορκαδίζων, ὅταν μέγας (/article/back/fn-group/fn/p/mixed-citation/inline-formula )’. Some parts of the text on the first folia represent a synopsis of Galen’s *On the Pulse for Beginners*, Karl Gottlob Kühn, *Claudii Galeni Opera omnia*, 20 vols (Leipzig: Carl Cnobloch, 1821–33), Vol. 8, 453–92. From folio 338r, the text is similar to the anonymous synopsis on pulses published by Charles Victor Daremberg and Charles-Émile Ruelle, *Oeuvres de Rufus d’Éphèse* (Paris: J.-B. Baillière, 1879), 219–32: (338r.l5–341r.l12) inc. ἠγνοήσαν ὅ δη τίς ὑπάρχει διαφορὰ σφυγμοῦ τὲ καὶ παλμοῦ, des. τοῦ πνεύματ(ος) ἐν τ(αῖς) ἀρτηρίαις ἀποτελεῖται /article/back/fn-group/fn/p/mixed-citation/inline-formula .

34. Jacques Goupyl*, Actuarii De actionibus & affectibus spiritus animalis, eisque victu, libri II* (Paris: Martinus Juvenus, 1557); Ideler, *op. cit.* (note 23); Ambrogio Leone (trans.), *De Urinis Actuarii Ioannis, Zachariae filii, medici praestantissimi, libri VII* (Basel: Andreas Cratandrus, 1529); Stavroula Georgiou, ‘Edition critique, traduction et commentaire critique du livre 1 “De Urinis” de Jean Zacharias Actouarios’ (unpublished PhD thesis: Paris, École Pratique des Hautes Études, 2013); Corneille Henri Mathys (trans.), *Actuarii Ioannis filii Zachariae Opera: De actionibus et spiritus animalis affectibus eiusque nutritione lib. II, De urinis lib. VII, Methodi medendi lib. V* (Paris: Bernardus Turrisanus, 1556); J. Enoch Powell, ‘Die letzten Tage der Grosskomnenen’, *Byzantinische Zeitschrift*, 37 (1937), 359–60; Ernst Gamillscheg, ‘Der Kopist des Par. gr. 428 und das Ende der Grosskomnenen’, *Jahrbuch der Österreichischen Byzantinistik*, 36 (1986), 287–300; repr. in *idem*, *Manuscripta Graeca: Studien zur Geschichte des griechischen Buches in Mittelalter und Renaissance* (Purkersdorf, 2010) [*Codices manuscripti*, Supplementum 3], 190–202; Paul Géhin and Matoula Kouroupou, *Catalogue des manuscrits conservés dans la Bibliothèque du Patriarcat Oecuménique: les manuscrits du monastère de la Panaghia de Chalki*, 2 vols (Turnhout: Brepols, 2008).

35. Demetrius Sicurus, *Theophili et Stephani Atheniensis De febrium differentia ex Hippocrate et Galeno* (Florence: Bencini, 1862); Johan Ludvig Heiberg, *Paulus Aegineta*, 2 vols (Leipzig: Teubner, 1921–24), [*CMG*, IX/1–2]; Alexander Olivieri, *Aetii Amideni libri medicinales*, 2 vols (Leipzig: Teubner, 1935–50), [*CMG*, VIII/1–2]; Kühn, *op. cit.* (note 33); Ideler, *op. cit.* (note 23); Sudhoff, *op. cit.* (note 23); Ivan Garofalo (ed.), *Anonymi medici De morbis acutis et chroniis* (Leiden: Brill, 1997).

36. The text has no Greek edition and has only been printed in a Latin translation by Leonhart Fuchs: *Nicolai Myrepsi Medicamentorum opus* (Basel: I. Opori, 1549). In contrast to the usual structure of the recipes given in the full version of the *Dynameron* such as in Parisinus gr. 2243 (AD 1339), where indication is followed by the list of substances and the method of preparation, here there is only the title followed by the list of ingredients for each recipe. This epitome comprises three parts: the first has, in no particular order, recipes from various parts of the *Dynameron*, the second focuses on oils, the third is an excerpt on plasters.

37. The first half of the text bears similarities with the Ps.-Galenic *Medical Definitions*; cf. Kühn, *op. cit.* (note 33), Vol. 19, 355.7–11.

38. The text is same as the work printed by Ideler, *op. cit.* (note 23), Vol. 1, 294–6 up to 47v, l.24: ‘(…) (δὲ) ὁ αντίχειρ ἔχει δύο·’ and constitutes a compilation of [Hippocrates’] *On Offspring, On the Nature of Child*, Émile Littré, *Oeuvres complètes d’Hippocrate*, 10 vols (Paris: J.-B. Baillière, 1839–61), Vol. 7, 470ff; here, the text carries on for 33 more lines. A slightly shorter version is also found in Parisinus gr. 2303, 83v–85r, and Parisinus gr. 2320, 1r–5r, and has been edited by Jouanna, *op. cit.* (note 15), 32–8.

39. Ed. Luigi Tartaglia, ‘Il lessico medico del codice Lond. Med. Soc. 60’, in Antonio Garzya and Jacques Jouanna (eds), *I testi medici greci – tradizione e ecdotica: Atti del III Convegno Internazionale, Napoli 15–18 ottobre 1997* (Naples: D’Auria, 1999), 547–57: 552–7; unfortunately, this edition skips the last five lines of text on fol. 50v.

40. The text here contains three more entries in addition to those in Heiberg, *op. cit.* (note 35), Vol. 2, 401–8.

41. Cf. Denis Petau, *Uranologion sive Systema variorum authorum, qui de sphaera, ac sideribus, eorumque motibus Graecè commentati sunt* (Paris: Sebastiani Cramoisy, 1630), 388ff.

42. It is noteworthy that, among others, it contains recipes of clearly oriental origin attested in late Byzantine medical works such as Nicholas Myrepsos’ *Dynameron* and John Zacharias Aktouarios’ *Medical Epitome*, eg. 89v ‘῾Η μικρὰ τρίφυλος· εἰς ἐξοχάδας ὠφελοῦσα: λαβὼν τὸν φλοιὸν τοῦ μυροβαλάνου τοῦ κέπουλε (…)’.

43. The chapters are usually abridged and their order varies largely in contrast to the version edited by Sicurus, *op. cit.* (note 35).

44. The opuscule on phlebotomy, 166r–169r, includes the term ‘βασιλική φλέψ’, found only in fifteenth-century Byzantine medical manuscripts and probably denoting an influence from Islamic medicine; see Oswei Temkin, ‘The Byzantine origin of the names for the basilic and cephalic veins’, in *idem*, *The Double Face of Janus and Other Essays in the History of Medicine* (Baltimore: Johns Hopkins University Press, 1977), 198–201.

45. The short text included between the contents of Books 2 and 3 accompanies the diagram of a urine vial and corresponds with John’s description of the urine vial in chapter 13 of Book 1.

46. The text is written in the form of ecclesiastical canon; some manuscripts attribute it to Maximos Planoudes; cf. Charle du Cange, *Glossarium ad scriptores mediae et infimae Graecitatis* (Paris: Hubertus Welter, 1688) Vol. 2, 1629, s.v. ὑέλιον; Ideler, *op. cit.* (note 23), 318; Aristote P. Kousis, ‘Les oeuvres médicales de Nicéphoros Blémmydès selon les manuscrits existants’, *Πρακτικὰ ᾿Ακαδημίας ᾿Αθηνῶν*, 19 (1944), 56–75: 71–4; Thanasis Diamandopoulos, *Μουσική Ουροσκοπία* (Patra: ᾿Αχαϊκὲς ᾿Εκδόσεις, 1995), 12–3.

47. Some parts of the text are abridged; from 200v the text follows very closely Ermerins’ edition, *op. cit.* (note 23), 33.14ff, with occasional gaps; the text here is sometimes supplemented with material not found in Ermerins’ edition; there is no diagram on pulses.

48. Littré, *op. cit.* (note 38); William Henry Samuel Jones, *Hippocrates, with an English Translation*, Vol. 4 (Cambridge MA: Harvard University Press, 1931); Bengt Alexanderson, *Textkritischer Kommentar zum Hippokratischen Prognostikon und Bemerkungen zu Galens Prognostikonkommentar* (Stockholm: Almqvist and Wiksell, 1968); Jacques Jouanna (ed.), *Hippocrate: Pronostic* (Paris: Les Belles Lettres, 2013); Ideler, *op. cit.* (note 23); Tartaglia, *op. cit.* (note 39); Heiberg, *op. cit.* (note 35); Kühn, *op. cit.* (note 33); Wilhelm Dindorf, *Epiphanii episcopi Constantiae Opera*, 5 vols (Leipzig: Weigel, 1859–62); R. Kunze, ‘Die Anonyme Handschrift (Da 61) der Dresdner königlichen Bibliothek “ΠΕΡΙ ΤΩΝ ΕΠΤΑ ΖΩΝΩΝ”’, *Hermes*, 34 (1899), 345–61; Angelo Maria Bandini, *Catalogus codicum Graecorum Bibliothecae Mediceae Laurentianae,* Vol. 3 (Florence: Typis regiis, 1770); Wilhelm Heinrich Roscher, ‘Die hippokratische Schrift von der Siebenzahl’, *Studien zur Geschichte und Kultur des Altertums*, 6, 3–4 (1913), 1–175; Francis Cumont, *Catalogus codicum astrologorum Graecorum* VIII. *Codices Parisini*, pt. 1 (Brussels: Regia Academia Belgica, 1929); Armand Delatte, ‘Geographica’, *Byzantinische Zeitschrift*, 30 (1929–30), 511–8; Pierre Boudreaux, *Catalogus codicum astrologorum Graecorum* VIII. *Codices Parisini*, pt. 4 (Brussels: Regia Academia Belgica, 1921); Stefan Weinstock, *Catalogus codicum astrologorum Graecorum* IX. *Codices Britannici*, pt. 2 (Brussels: Regia Academia Belgica, 1953); Armand Delatte, *Catalogus codicum astrologorum Graecorum* X. *Codices Athenienses* (Brussels: Regia Academia Belgica, 1924); Francis Cumont, *Catalogus codicum astrologorum Graecorum* VIII. *Codices Parisini*, pt. 4 (Brussels: Regia Academia Belgica, 1921); Karl Krumbacher, ‘Studien zu den Legenden des hl. Theodosius’, *Sitzungsberichte der philosophisch-philologischen und der historischen Classe der K. B. Akademie der Wissenschaften zu München*, (1892), 220–379; Nikolai Serikoff, “‘Syriac” plant names in a fifteenth century Greek glossary’, in Barbara Zipser (ed.), *Medical Books in the Byzantine World* (Bologna: Eikasmos, 2013), 97–121; Maria Capone Ciollaro, *Demetrio Pepagomeno: Prontuario medico* (Naples: Bibliopolis, 2003); Sicurus, *op. cit.* (note 35); August B. Langkavel, *Simeonis Sethi Syntagma de alimentorum facultatibus* (Leipzig: Teubner, 1868); Leone, *op. cit.* (note 34); Georgiou, *op. cit.* (note 34); Mathys, *op. cit.* (note 34); Kousis, *op. cit.* (note 46); Ermerins, *op. cit.* (note 23).

49. Curt Wachsmuth, *Ioannis Laurentii Lydi Liber de ostentis, et calendaria Graeca omnia* (Leipzig: Teubner, 1897); Olivieri, *op. cit.* (note 35).

50. Probably the metropolitan of Imbros attested ca. 1548–80: Martin Crusius, *Turcograecia* (Basel: Leonardus Ostenius, 1584), 287, 318.

51. On Euthymios, Symeon (d. 1652), and Metrophanes (d. 1655): Sotiris K. Kadas, *Τὰ σημειώματα τῶν χειρογράφων τῆς Μονῆς Διονυσίου ῾Αγίου ῎Ορους* (Mount Athos: Μονή Διονυσίου, 1996), 144 (no. 435).

52. Franz Zacharias Ermerins, *Aretaei Cappadocis quae supersunt* (Utrecht: Kemink, 1847); Karl Hude, *Aretaeus*, 2nd edn (Berlin: Academia Scientiarum, 1958) [*CMG*, II]; Ioannis Vassis, *Initia carminum Byzantinorum* (Berlin: De Gruyter, 2005).

53. Daremberg, *op. cit.* (note 33); Georg Kowalski, ‘Rufi Ephesii De corporis humani pertium appellationibus’ (unpublished PhD thesis: University of Göttingen, 1960).

54. The order of chapters is almost the same as in the edition by Skevos G. Zervos, ‘᾿Αετίου ᾿Αμιδηνοῦ λόγος ἔνατος’, *΄Αθηνᾶ*, 23 (1911), 265–392: 272–390.

55. The book has no Greek edition and has only been printed in Latin translations: Giovanni Battista da Monte (trans.), *Aetii Amideni Libri sexdecim nunc primum latinitate donati*, 3 vols (Venice: Lucas Antonius Junta, 1535); Janus Cornarius (trans.), *Aetii medici graeci contractae ex veteribus medicinae tetrabiblos* (Lyons: Godefridus et Marcellus Beringi fratres, 1549). Books 8–13 are identical in these two; here, I provide reference to both. The order of chapters in the manuscript sometimes differs from that of the printed Latin translations.

56. The order of chapters differs considerably from that in Daremberg, *op. cit.* (note 33), 85–126: 568–81.

57. The order of chapters is generally the same as in Georgios A. Costomiris (ed.), *᾿Αετίου λόγος δωδέκατος* (Paris: Klincksieck, 1892); some chapters are supplemented here with further recipes, while the last two chapters printed in Costomiris are missing.

58. The book has been partly edited by Skevos G. Zervos, ‘᾿Αετίου ᾿Αμιδηνοῦ δακνόντων ζῴων καὶ ἰοβόλων’, *᾿Αθηνᾶ*, 18 (1906), 241–302: 264–92. For chapters not found in Zervos’ edition, I provide references to Da Monte and Cornarius, *op. cit* (note 55). Sometimes the text differs considerably from the printed edition and the Latin translations; in one case, an entire chapter is printed neither in Zervos nor in the Latin translations, viz. 169v: **Περὶ ἀσφαλάκων**, inc. Εἰ θέλεις ἀσφαλάκ(ους) διαφθεῖραι, des. κ(αὶ) τοῦτο ποιήσ(ας) πάντας διαφθείρεις.

59. The book has no Greek edition and is only available in Latin translations by Da Monte and Cornarius, *op. cit.* (note 55); here, I provide reference to both. The order of chapters in the manuscript sometimes differs from that in the printed Latin translations.

60. The order of chapters is almost the same as in Skevos G. Zervos (ed.), ‘᾿Αετίου ᾿Αμιδηνοῦ λόγος δέκατος πέμπτος’, *᾿Αθηνᾶ*, 21 (1909), 3–144: 7–141.

61. Zervos, 1911, *op. cit.* (note 54); Da Monte, *op. cit* (note 55); Cornarius, *op. cit.*, (note 55); Daremberg, *op. cit* (note 33); Costomiris, *op. cit.* (note 57); Zervos, 1906, *op. cit.* (note 58); Zervos, 1909, *op. cit.* (note 60).

62. Cf. Nigel G. Wilson, *A Descriptive Catalogue of the Greek Manuscripts of Corpus Christi College Oxford* (Cambridge: D.S. Brewer, 2011), 16–17 with pls. 33, 35–36. This reference was kindly pointed out to me by Rudolf Stefec.

63. Books 3–6 have no Greek edition and have been published only in Latin translation: Mathys, *op. cit.* (note 34).

64. Mathys, *op. cit.* (note 34).

65. Raymond Gaudriault, *Filigranes et autres caractéristiques des papiers fabriqués en France aux XVIIe et XVIIIe siècles* (Paris: CNRS; J. Telford, 1995).

66. Heiberg, *op. cit.* (note 35).

67. Mošin and Traljić, *op. cit.* (note 24).

68. Leone, *op. cit.* (note 34); Georgiou, *op. cit.* (note 34); Ideler, *op. cit.* (note 23); Mathys, *op. cit.* (note 34).

69. *The Nostitz Papers: Notes on Watermarks Found in the German Imperial Archives of the 17th & 18th Centuries, and Essays Showing the Evolution of a Number of Watermarks* (Hilversum: Paper Publications Society, 1958).

70. Johannes Raeder, *Oribasii Collectionum medicarum reliquiae*, 2 vols [*CMG*, VI/1,1–2,2] (Leipzig: Teubner, 1928–33).

71. The current folia 1–16 have an earlier foliation, 1–12.

72. The order of chapters is sometimes the same as that in the edition by Johann Stefan Bernard, *Theophanis Nonni Epitome de curatione morborum*, 2 vols (Gotha: Ettinger, 1794–5). However, there are also lots of variations on the text (which is usually longer) and the headings; the proem and last chapters on venomous animals are not included.

73. The text is close to Ideler’s edition, *op. cit.* (note 34), Vol. 2, 257–81, although shorter, and its end coincides with Ermerins’ edition, *op. cit.* (note 23), 225–75; the proem preceding the text is similar to the one edited by Laura Felici, ‘L’opera medica di Teofane Nonno in manoscritti inediti’, *Acta medicae historiae Patavina*, 28 (1981–2), 59–74: 67–8.11628817

74. The introduction follows the edition of the relevant part printed by Felici, *op. cit.* (note 73), 63, based on Marcianus gr. V 16, ff. 129r–v, 140–146, and Vaticanus gr. 292, ff. 68v–96. First recipe on theriac; last recipe on the plaster called μαστιχηρά.

75. The order of chapters is largely different from Langkavel’s edition, *op. cit.* (note 48); chapters usually appear in an abridged version, eg. the entry Περὶ ῥαφανίδος appears twice: once (135r–136r) in a version close to Langkavel’s edition and one more (114r) in an abridged form.

76. Bernard, *op. cit.* (note 71); Felici, *op. cit.* (note 72); Ermerins, *op. cit.* (note 23); Ideler, *op. cit.* (note 23); Langkavel, *op. cit.* (note 48).

77. The second sentence is a saying attributed to Saint Crispin of Viterbo (1668–1750).

78. Vladimir Mošin, *Anchor Watermarks* (Amsterdam: Paper Publication Society, 1973); and Heawood, *op. cit.* (note 14).

79. The order of paragraphs sometimes does not match Kühn’s edition, *op. cit.* (note 33), Vol. 19, 346–462; some chapters appear in an abridged version.

80. Kühn, *op. cit.* (note 33).

81. Mark Sosower, *Signa officinarum chartariarum in codicibus Graecis saeculo sexto decimo fabricatis in bibliothecis Hispaniae* (Amsterdam: Haakert, 2004).

82. The text follows the usual structure of commentaries on Hippocratic *Aphorisms* providing the aphorism accompanied by commentary; the commentary is preceded by ‘ἑρμ(η)ν(εία)’ or ‘σχόλ(ιον)’. Here the commentary follows the version attributed in other manuscripts to Damascius; Caroline Magdelaine, ‘Le commentaire de Damascius aux Aphorismes d’Hippocrate’, in Antonio Garzya and Jacques Jouanna (eds), *Storia e ecdotica dei testi medici greci: Atti del II Convegno Internazionale, Parigi 24–26 maggio 1994* (Napoli: D’Auria, 1996), 289–306: 292–3. Since the version of the aphorisms in the manuscript is not always identical with the text published by Friedrich Reinhold Dietz, *Scholia in Hippocratem et Galenum*, 2 vols (Königsberg: Borntraeger, 1834), Vol. 2, 250–543, for each aphorism I provide the references to Littré and Jones, and then to Dietz’s edition of Damascius’ commentary. The Wellcome MS contains the full text of the commentary published by Dietz, followed (18r–v) by *Aphorisms* 7.62–3, 65–75 without commentary. Each book has a separate heading (1r, 3v, 6r, 8v, 11r, 14r, 16r).

83. Chapter titles written in the margins of ff. 18v–21v.

84. The text in this MS is incomplete and breaks off at the same point (II.1) as in New Haven, Yale University, Cushing/Whitney Medical History Library, MS 50 (*olim* Saragossa, Cabildo de la Santa Iglesia Mayor del Pilar, MS 1427); John Duffy, *Stephani Philosophi In Hippocratis Prognosticum commentaria**III* (Berlin: Akademie-Verlag, 1983), [*CMG*, XI/1,2], 18, concludes that the Wellcome MS is a direct copy of the Yale one.

85. Littré, *op. cit.* (note 38); Jones, *op. cit.* (note 48); Dietz, *op. cit.* (note 82); Alexanderson, *op. cit* (note 48); Jouanna, *op. cit.* (note 48).

86. Duffy, *op. cit.* (note 84).

87. Peter Lambeck (ed.), ‘Imperatori Leonis cognomine Sapientis Oracula’, in Jacques Paul Migne (ed.), *Patrologiae cursus completus: Seria Graeca* (Paris: Migne, 1863), Vol. 107, 1121–40; Walter G. Brokkaar *et al.*, *The Oracles of the Most Wise Emperor Leo & The Tale of the True Emperor* (Amsterdam: Leerstoelgroep Nieuwgriekse Taal en Letterkunde en Byzantinologie, 2002); Phaidon K. Bouboulidis, ‘῾Ιστορημένα χρησμολόγια τοῦ Μουσείου Διον. Λοβέρδου’, *᾿Επετηρὶς ῾Εταιρείας Βυζαντινῶν Σπουδῶν*, 38 (1971), 208–23; Spyridon Lampros, ‘Τὸ ὑπ’ ἀριθμὸν ΛΘ΄ κατάλοιπον’, *Νέος ῾Ελληνομνήμων*, 19 (1925), 97–124; Katerina Kyriakou, *Οι ιστορημένοι χρησμοί του Λέοντος ΣΤ΄ του Σοφού: χειρόγραφη παράδοση και εκδόσεις κατά τους ΙΕ΄-ΙΘ΄ αιώνες* (Athens: Σύλλογος πρὸς Διάδοσιν ᾿Ωφελίμων Βιβλίων, 1995).

88. [Ioannis Karatzas and Athanasios Psalidas], *῎Ερωτος άποτελέσματα ἤτοι ῾Ιστορίαι ἠθικοερωτικαὶ μὲ διάφορα ἐν τῷ μεταξὺ χαρίη καὶ ἐρωτικά τραγούδια*, 2nd edn (Venice: Andreola, 1836).

89. Georg Eineder, *The Ancient Paper-Mills of the Former Austro-Hungarian Empire and Their Watermarks* (Hilversum: Paper Publications Society, 1960).

90. Based on Rudolf Stefec’s identification of hand B with that of Manuel Korinthios, attested at the Greek Patriarchate of Constantinople in 1481–1507.

91. The current foliation is in the lower left-hand corner of each recto. An older foliation (1–87, incl. front flyleaf) is found in the upper left corners.

92. Acrostics ὁ ῥήτωρ – Μανουήλ – ὁ ῥήτωρ.

93. Acrostics ὁ ῥήτωρ.

94. Acrostic Μανουὴλ ὁ ῥήτωρ.

95. Vasileios K. Stephanidis, ‘Στίχοι Μανουὴλ τοῦ μεγάλου ῥήτορος’, *Byzantiniscshe Zeitischrift*, 17 (1908), 470; Wolfram Hörandner, ‘Visuelle Posie in Byzanz: Versuch einer Bestandsaufnahme’, *Jahrbuch der Österreichischen Byzantinistik*, 40 (1990), 1–42; Vassis, *op. cit.* (note 52); Maximilian Treu, ‘Manuel Holobolos’, *Byzantiniscshe Zeitischrift*, 5 (1896), 538–59; David Pingree, *Hephaestionis Thebani Apotelesmaticorum libri tres*, 2 vols (Leipzig: Teubner, 1974); Odysseas Lampsides, ‘Georges Chrysococcis, le médecin, et son oeuvre’, *Byzantiniscshe Zeitischrift*, 38 (1938), 310–22; Peter Solon, ‘The “Hexapterygon” of Michael Chrysokokkes’ (unpublished PhD thesis: Brown University, 1968); Leendert Gerrit Westerink, *Michael Psellus: De omnifaria doctrina* (Nijmegen: Centrale Drukkerij, 1948).

96. On Manuel Korinthios, see Rudolf Stefec, ‘Zwischen Urkundenpaläographie und Handschriftenforschung: Kopisten am Patriarchat von Konstantinopel im späten 15. und frühen 16. Jahrhundert’, *Rivista di studi bizantini e neoellenici*, ser. II, 51 (2014), in press.

97. Heawood, *op. cit.* (note 14).

98. On this ex-libris, engraved by Lucas Kilian (1579–1637): Sibylle Appuhn-Radtke, ‘Augsburger Buchillustration im 17. Jahrhundert’, in: Helmut Gier and Johannes Janota (eds), *Augsburger Buchdruck und Verlagswesen* (Wiesbaden: Harrassowitz, 1997), 735–90: 758.

99. Klaus Eckart Ehrlicher, ‘Ein steirisches Adelsgeschlecht in Böhmen und Mähren: Hoffmann Freiherren zu Gruenpüchel und Strechau’, *Bohemia: Zeitschrift für Geschichte und Kultur der böhmischen Länder*, 21 (1980), 70–83.

100. Paolo Divizia, ‘Una lettera in lingua italiana di Ferdinand Hoffmann von Gruenpüchel und Strechau’, *Études Romanes de Brno*, 37 (2007), 159–65: 160.

101. Judging from the frequent reduction of ‘ο’ to ‘ου’, eg. ‘του παιδή’ for ‘το παιδή’.

102. Many leaves are lost. There is no continuous modern foliation.

103. It contains instructions for diagnosis and phlebotomy, various recipes of composite drugs (generally structured *a capite ad calcem*), spells, short texts on auspicious days and on dream interpretation, moon-tables, and zodiacal tables. The text is not clearly organised and the original order of leaves remains uncertain.

104. Stefan Andreev, *Les filigranes dans les documents ottomans: couronne* (Sofia: Trayanov, 2007).

